# The unsuitability of implantable Doppler probes for the early detection of renal vascular complications – a porcine model for prevention of renal transplant loss

**DOI:** 10.1371/journal.pone.0178301

**Published:** 2017-05-25

**Authors:** Chris Amdisen, Bente Jespersen, Ulla Møldrup, Anna K. Keller

**Affiliations:** 1 Institute of Clinical Medicine, Aarhus University Hospital, Aarhus, Denmark; 2 Department of Renal Medicine, Aarhus University Hospital, Aarhus, Denmark; 3 Department of Urology, Aarhus University Hospital, Aarhus, Denmark; The University of Manchester, UNITED KINGDOM

## Abstract

**Background:**

Vascular occlusion is a rare, but serious complication after kidney transplantation often resulting in graft loss. We therefore aimed to develop an experimental porcine model for stepwise reduction of the renal venous blood flow and to compare an implantable Doppler probe and microdialysis for fast detection of vascular occlusion.

**Methods:**

In 20 pigs, implantable Doppler probes were placed on the renal artery and vein and a microdialysis catheter was placed in the renal cortex. An arterial flowprobe served as gold standard. Following two-hour baseline measurements, the pigs were randomised to stepwise venous occlusion, complete venous occlusion, complete arterial occlusion or controls.

**Results:**

All parameters were stable through baseline measurements. Glutamate and lactate measured by microdialysis increased significantly (p = 0.02 and p = 0.03 respectively) 30 minutes after a 2/3 (66%) reduction in renal blood flow. The implantable Doppler probe was not able to detect flow changes until there was total venous occlusion. Microdialysis detected changes in local metabolism after both arterial and venous occlusion; the implantable Doppler probe could only detect vascular occlusions on the vessel it was placed.

**Conclusions:**

We developed a new model for stepwise renal venous blood flow occlusion. Furthermore, the first comparison of the implantable Doppler probe and microdialysis for detection of renal vascular occlusions was made. The implantable Doppler probe could only detect flow changes after a complete occlusion, whereas microdialysis detected changes earlier, and could detect both arterial and venous occlusion. Based on these results, the implantable Doppler probe for early detection of vascular occlusions cannot be recommended.

## Introduction

Kidney transplantation improves survival rates in addition to improving health and quality of life in end stage renal failure[1–3]. However, access to kidney grafts is limited and early graft loss is potentially life threatening for the recipient. Protection of the transplanted organs is thus essential and is achieved by continuous advances in immunosuppressive regimens, surgical techniques and perioperative managements. However, although vascular occlusions are rare, they still account for up to 35% of the grafts lost within the first 30 days after transplantation[4–6], and their elimination is crucial to improved outcomes[5,7–9].

Several risk factors for renal graft thrombosis have been identified. The most important are: donor-age (<6 or >60 years of age), per- or postoperative hemodynamic instability, a history of thrombosis or more than 24 hours of cold ischemia time[5,10–14].

Postoperatively, the function of the transplanted kidney is often monitored by two main parameters—the function of the kidney and the renal blood flow. The renal function is measured as p-creatinine and hourly diuresis. If graft function is delayed or deteriorates, blood flow in the vessels can be evaluated by ultrasonography[15,16].

The implantable Doppler probe (iD-probe) has been used for several years by reconstructive surgeons on free flaps[1–3], and a recent report suggested its usability in kidney transplantations[4–6]. However the probe may only detect a full flow stop, which will often be too late for salvage of the graft and furthermore, the technique has, to our knowledge, not been tested in a standardised setup.

Microdialysis provides insight into the local metabolism[5,7–9]. This method has been used in experimental and clinical settings and detects renal ischemia within 20–30 minutes[5,10–14], but it requires continuous sample evaluation and a small catheter in the kidney.

The aim of this study was to establish an experimental animal model for gradual renal vein occlusion and to investigate if the ultrasound probe could detect occlusion before microdialysis detected metabolic changes.

## Materials & methods

### Ethics

The study was approved by the Danish National Animal Ethics Committee (no. 2013-15-2934-00770).

### Design and anaesthesia

Twenty 40 kg female Danish landrace/Yorkshire pigs were used. The animals fasted overnight, with free access to water. Anaesthesia was induced with intravenous Midazolam (0.5 mg/kg) and Ketamine (5 mg/kg) before intubation. Anaesthesia was maintained with sevoflurane (MAC 1.1–1.4) and Fentanyl (12.5 mg/h/kg). Mechanical ventilation was set to 40% oxygen and tidal volume of 10 mL/kg. Expiratory CO_2_ was kept between 4.5 and 5.5 kPa. Cefuroxim 750 mg, was administered intravenously before the surgical procedure as antibiotic prophylaxis.

The carotid artery and jugular vein were catheterised for blood pressure monitoring, collecting blood samples and infusion of drugs. Both ureters were catheterized for selective urine collection.

The animals were kept normohydrated by continuous saline infusion (12.5 mL/kg/h). Mean arterial pressure (MAP) was maintained above 60 mmHg by extra saline infusions as appropriate. At the end of the experiment, the animals were euthanized with a lethal dose of pentobarbital (80 mg/kg) while still anesthetised.

All pigs underwent the same surgical procedure. Through a midline incision both kidneys were exposed retroperitoneally. On the right side, the renal vein, artery and lumbar veins were exposed, and the ureter was catheterized. On the left side, the renal vein was dissected till vena cava, and all side branches, except the lumbar veins were ligated. Upon dissection of the artery, 0.5–1 mL papaverin (3 mg/mL) was injected into the arterial wall to avoid spasms. A flowprobe (MEDISTIM quickfit TTFM Probe, 3 mm) was left on the artery to measure arterial flow, which was used as a reference for vascular interventions.

After the surgical procedure, the pigs were allowed a stabilization period of one hour.

In the left kidney, an iD-probe was placed on the renal artery (iDa-probe) and the renal vein (iDv-probe). A cortical microdialysis catheter and a reference flow probe (Medistim) were also placed on the renal artery. Following two hours of baseline samples, the animals were randomised into four groups: stepwise venous occlusion, (n = 8), complete venous occlusion (n = 4), complete arterial occlusion (n = 4) or controls (n = 4).

Every 15 minutes, microdialysis samples were harvested and the iDa-probe and iDv-probe signals were noted. Every 30 minutes, the diuresis was measured and arterial blood gas analysed.

### Stepwise venous occlusion group

**Fig 1:**
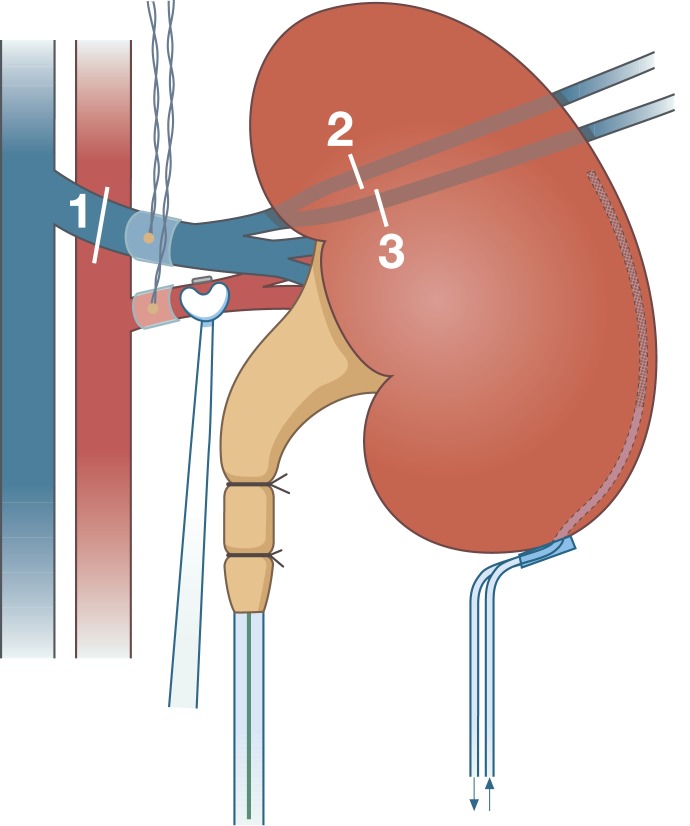
Implantable Doppler probes placed on both the renal vein and artery. A microdialysis catheter placed in the renal cortex and the reference flow probe placed on the renal artery. A catheter is placed in the ureter to collect urine. Lines and numbers indicates the stepwise intervention.

A model for stepwise reduction of renal venous output was developed (Fig 1). By ligation of the renal vein [1], central of the lumbar veins, the venous blood was directed retrograde through the lumbar veins, which functioned as a long stenosis, leading to reduced blood flow. Further reductions in blood flow were achieved by ligation of the two lumbar veins [2,3] four and six hours after baseline respectively. After the last intervention, the pigs were observed for an additional two hours.

### Complete occlusion groups

In the complete venous and arterial occlusion groups, blood flow was reduced by ligation of either the renal vein or artery respectively, after a two-hour baseline period. The pigs were observed for additional three hours after the intervention.

### Implantable Doppler probe

The implantable Doppler (iD) probe (20 MhZ, long cuff)(Cook-Swartz Doppler Flow probe, Cook Medical, Ireland) was placed on both renal vessels, the iDv-probe and the iDa-probe (Fig 1). The iDv-probes were secured around the vein by sutures and the iDa-probes were attached by a small clamp on the silicone cuff. The iD-probe has a continuous binary outcome as sound/no sound. This cannot be quantified due to the design. Every 15 minutes it was noted whether it had become silent or still gave an audio signal.

Throughout this paper, the terms Doppler and Doppler prober strictly refers to the implantable Doppler probe used, and not to any other type of Doppler probes.

### Microdialysis

A microdialysis catheter (membrane length: 30 mm, cut-off: 20.000 Dalton, inlet tube: 100 cm, outlet tube 25 cm)(CMA Microdialysis, Sweden) was inserted into the renal cortex, using a 21G-sized needle for tunnelling. It was perfused with T1 perfusion fluid at a flow rate of 0.5 μL/minute by a CMA400 pump.

At 15 minute intervals, microdialysis samples were collected and frozen immediately. These were later thawed, centrifuged and analysed for glucose, glutamate, glycerol and lactate on a CMA600 microdialysis analyser. Outliers were defined as values, which varied more than 80% from the adjacent values in the series; the conclusion was similar if these specimens were not excluded.

### Statistics

One-way Anova analysis of baseline values was performed for all microdialysis metabolites. Baseline values were merged afterwards into one mean baseline value. A paired t-test was performed on the first two values after the first intervention. We only analysed these, as we wanted a prompt response from the equipment. All data are presented as mean. Error bars represents ± 95 confidence intervals.

Results were considered significant if p<0.05.

All statistics were made using GraphPad Prism version 5.0 for Mac OS X.

## Results

The experiment was completed in all 20 animals.

### Arterial renal blood flow

**Fig 2:**
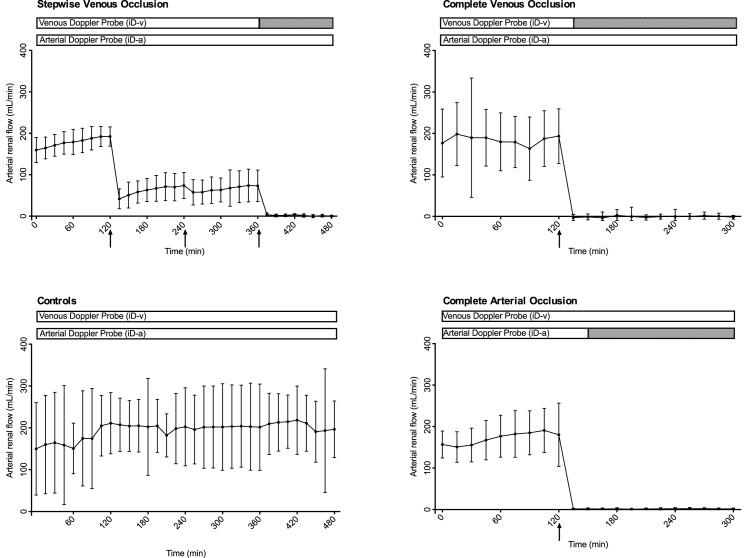
Flow measurements. Flow measured by the reference flow probe and the implantable Doppler probes. White box means flow and black box, no flow. The time-point of no flow is an average between the probes. The interventions are marked with arrows. The 95% confidence intervals are shown.

During baseline, the arterial renal flow profiles, produced by the reference flow probe on the renal artery, were similar in the four experimental groups (Fig 2).

In the stepwise venous occlusion group, there was a significant decrease in arterial renal flow after the first intervention, with a 2/3-flow reduction. At the second intervention, no significant decreases in arterial renal flow were noted. At the third intervention, when the venous flow was completely obstructed, the arterial flow stopped (p<0.001). In the controls, there were no flow changes throughout during the experiments (one-way Anova, p = 0.994). Following complete venous and complete arterial occlusion, the arterial flow was reduced to zero immediately after the intervention.

### Implantable Doppler probe

In 3 of 40 iD-probes, the Doppler crystal was dismantled from the silicone cuff when carrying out other procedures and a new probe was placed. This happened during some of the first animals.

In all four groups, both iD-probes gave a consistent signal during the baseline period (Fig 2). In the stepwise venous occlusion group, no changes in signal were noted after the first intervention, neither on the venous nor the arterial side. After the second intervention one venous probe lost signal at t = 255. At t = 375, immediately after the third intervention, all venous probes had lost signal, though one of the venous probes had a delay, and signal was lost at t = 390. All arterial probes gave a signal throughout the experiment and were unaffected by the venous occlusion. In the controls all probes gave a consistent signal throughout the experiment from both the artery and the vein, except one, where a clear venous signal was not obtainable.

Following complete venous occlusion, there was no signal from the venous probes, and a complete stop in arterial flow was noted at t = 135, 15 min after the intervention. The arterial probes were not able to detect the venous occlusion in any of those experiments. Complete arterial occlusion caused immediate loss of signal from the arterial iD-probes. In three arterial probes the signal stopped at t = 135. The last arterial probe had a delay, and signal was lost at t = 195, 75 minutes after complete arterial occlusion. One venous probe signal stopped at t = 195, while the remaining three did not detect the arterial occlusion.

### Microdialysis

A total 57 of 544 samples were not achieved. Of these, 45 were due to faulty catheters and 12 were missing due to faults during analysis. Further, some values were excluded from the data analysis as outliers.

**Fig 3:**
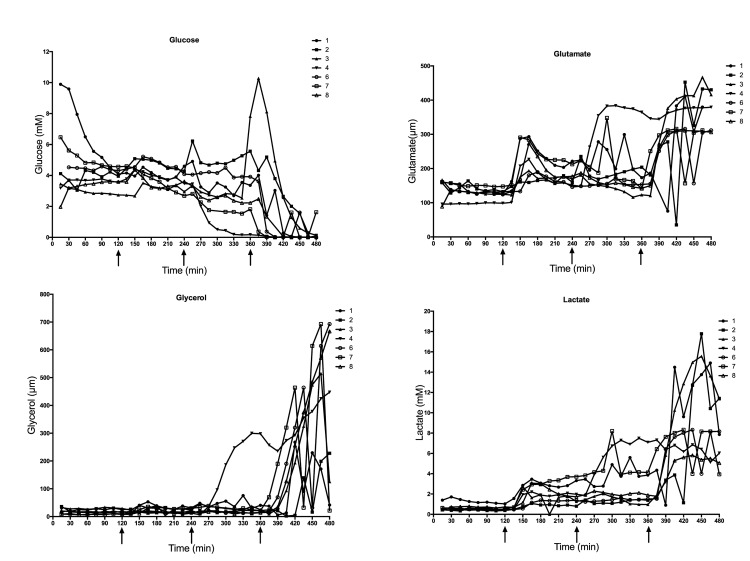
Microdialysis metabolites in individual animals. Levels of glucose, glutamate, glycerol and lactate in the individual animals measured by microdialysis after stepwise venous occlusion. Arrows indicates the three interventions.

**Fig 4:**
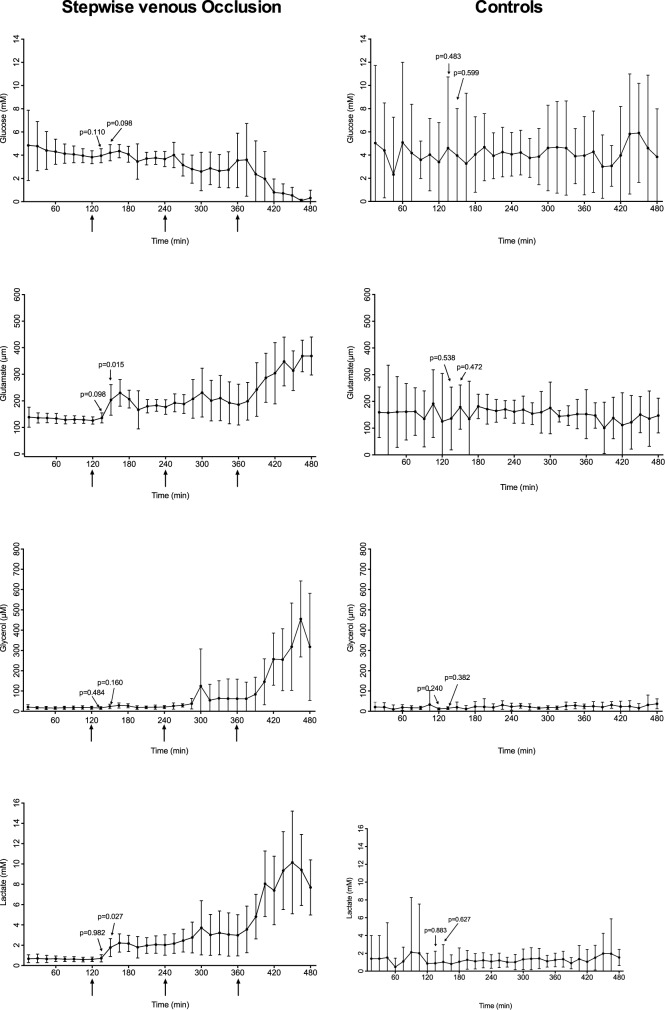
Microdialysis metabolites after stepwise venous occlusion and controls. Levels of glucose, glutamate, glycerol and lactate measured by microdialysis after stepwise venous occlusion or controls. Arrows indicates the three interventions. The 95% confidence intervals are shown. P-values are estimated using baseline values as reference.

**Fig 5:**
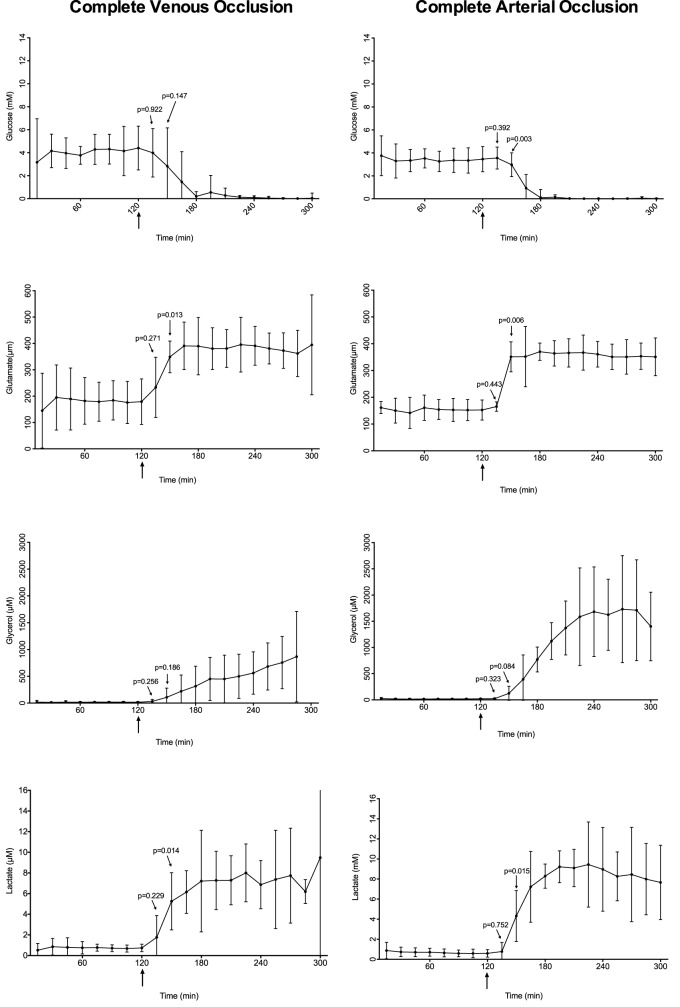
Microdialysis metabolites after complete venous and complete arterial occlusion. Levels of glucose, glutamate, glycerol and lactate measured by microdialysis after complete venous occlusion or complete arterial occlusion. Arrows indicates the intervention. The 95% confidence intervals are shown. P-values are estimated using baseline values as reference.

During baseline, all four metabolites measured by microdialysis remained stable in all four groups. In the controls the metabolites remained consistent, and reflected the baseline values in the other experimental groups throughout the experiment (Figs 3–5).

As shown in Figs 3 and 4, glucose remained stable through the first two venous interventions and then showed a falling trend 30 minutes after the third intervention. Glutamate increased significantly (p = 0.015) 30 minutes after the first intervention at t = 150. Glycerol levels did not increase until after the third intervention. Lactate increased within 30 minutes.

As seen in Fig 5, the arterial and venous occlusion group showed similar tendencies. Glutamate and lactate both had a faster reaction to the vascular occlusion than glycerol and glucose.

### Urine production

**Fig 6:**
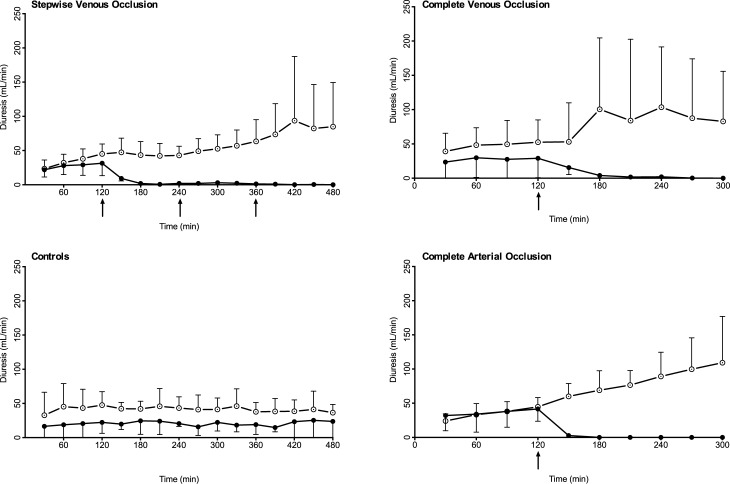
Urine production. Diuresis during the experiment. Arrows indicates interventions. Dotted circles indicates the right (control) kidney and black circles indicates the left kidney. 95% confidence intervals are shown.

The urine production for all four groups is depicted in Fig 6. In the gradual venous occlusion group, the urine production had almost stopped completely one hour after the first intervention; this was also noted in the complete venous occlusion group. In the complete arterial occlusion group, the urine production stopped 30 minutes after the intervention, and it remained zero throughout the experiment. As expected the diuresis of the right kidney increased significantly (p<0.005) after the interventions.

## Discussion

In a new model of stepwise renal venous occlusion, it was demonstrated that the implantable Doppler probe detected flow changes much later than microdialysis detected metabolic changes. Further the probe only detected occlusions in the vessel where it was placed and did not detect serious flow problems related to occlusion in other vessels. In contrast, significant changes in the local metabolism were detected within 30 minutes by microdialysis, both after total venous and arterial occlusion, and 2/3 (66%) venous occlusion, whereas the iD-probe only detected full occlusion.

Prior to this study, other models for simulating venous thrombosis have been studied. A thrombosis model previously used on skin flaps[15,16] proved difficult to standardise and the same applied to use of an adjustable clamp[17]. Our research team has shown at an earlier stage that microdialysis can detect arterial and venous clamping, and also stepwise arterial occlusion[11,18]. The presented model for stepwise renal venous occlusion was established to simulate the development of renal vein thrombosis after kidney transplantation. The model was designed to control the flow in the renal vein, and to stop flow at three intervals during four hours, a situation known from a postoperative venous thrombosis in the renal transplantation setting. The first intervention resulted in a reduction to 1/3 in renal blood flow. The second intervention did not cause any further flow changes and the third intervention stopped the blood flow. Thus the model functioned as a two-step model rather than the intended three step-model, but it was highly reproducible.

The probe is simple to apply, albeit there is a small learning curve as reported in other studies[19,20]. The present study was performed after a pilot period for such learning. The continuous signal from the probe is easy to interpret, the method is minimally invasive, adding no extra surgical trauma to the patient, and adding only a little extra operating time[20,21]. The probe can be left in the patient for up to seven days and it is easily removed, as the crystal will detach from the silicone cuff, with a light pull. The silicone cuff remains in the patient.

If the probe is attached too tightly, it may obstruct flow. This problem was not encountered in this study. Furthermore, even though this potential problem is mentioned in the literature, it has not been described yet[22,23]. The probe was not removed in this study. We therefore did not encounter any problems with probe removal. In a clinical setting, the probe would be kept for days rather than hours.

The probe was developed for monitoring of free skin flaps[24]. One probe was used for monitoring of both the artery and the vein (as an arterial flow stop in the small vessels to the flap, would also result in a venous flow stop). The probe has showed promising results for detecting vascular occlusions and improving salvage rates[1,19,20,22]. However, two studies have shown that neither the probe nor microdialysis are as effective as clinical monitoring[25,26].

The probe has also been applied to liver transplants[21], to detect early hepatic arterial thrombosis. In the CONDOR study, 102 patients undergoing liver transplantation had the probe implanted. The probe increased the accuracy of the diagnosis, but it had a low sensitivity of only 60%, and could thus not replace the external ultrasonography evaluation.

Crane et al.[4] tested the iD-probe on 15 uncomplicated kidney transplantations and concluded that it could potentially save organs. We found that the iD-probe was able to detect a full stop of flow in the renal artery and vein, but that an arterial iD-probe was not able to detect venous occlusions and vice versa. Hence two probes would be needed. In the two venous occlusion groups, the reference flowprobe on the artery confirmed that there was no flow in the artery after complete venous occlusion. The reference flowprobe revealed that there was a column of pulsating blood in the artery not entering the kidney, causing the iDa-probe to give signal. While these iDa-probes did not lose signal after complete occlusion, a difference could be heard in the audio signal as the signal became less clear after an intervention. This was also seen in the Condor liver study[21]. The clinical relevance of this is minor, as it is unlikely to be the same person interpreting the signal throughout the observation period, and different people might not hear such slight differences in signal.

If the iD-probe was able to translate the audio signal into a continuous flow output, the results of this study might have been completely different, as some of the major limitations of the iD-probe would have been overcome. With a continuous flow output, the iD-probe would probably have been able to detect reduced flow, already after the first intervention, and give warning before the graft was lost. Therefore, translation of sound into another signal the iD-probe could probably be developed for clinical use in renal transplantation, and renal transplantation, with appropriate alarms could be introduced.

Microdialysis is based on diffusion over a semipermeable membrane. The microdialysis catheter is placed in the tissue of interest for the monitoring of the local metabolism. It is easy to apply, samples are easily obtained and analysed and it is minimally invasive. Furthermore, microdialysis is a validated method for detection of renal ischemia in experimental setups[7,10,14,27,28]. There are, however, some drawbacks to the microdialysis technique as well. The analysis for four metabolites takes about six minutes, and the results have to be considered based on earlier samples. This creates a delay in real time observation. In this study, two out of four metabolites gave an early warning of ischemia within 30 minutes after a 2/3 (66%) reduction in renal venous blood flow. In our setup, we had a perfusion rate at 0.5 μL per minute and an outlet tube of 25 cm, creating a delay between catheter and outlet of nine minutes. This prolongs the reaction time of the microdialysis, and with a higher perfusion rate and a shorter outlet tube, we might have been able to detect flow changes even earlier. However, the samples would have been diluted and this may distort such an apparent advantage. A solution to this problem could possibly be to use two microdialysis catheters, one with a fast perfusion rate and one with a slower rate enabling further analysis of the perfusion rate. The microdialysis results showed some variations, which emphasises that it is better to consider the individual patient in changing trends, rather than absolute values. It would still be necessary though, with cut-off values for clinical use, which are also stressed in other studies[10,13].

In this model of stepwise venous occlusion, glucose was seen to have a slow reaction to flow changes. This is probably because there is still some glucose delivery to the kidney from the arterial supply through the first two interventions. Traditionally, glycerol has been viewed as one of the first markers for ischemia and cell damage[10,14,29]. However, this is not in line with our results which show glycerol and glucose as slow markers first giving a warning after the last venous intervention and then lactate and glutamate as fast markers, increasing after the first intervention. Glycerol is a marker of irreparable cell damage[30]. If the lactate and glutamate levels were considered and used as primary markers for vascular occlusion, it would be possible to react before glycerol levels start to increase and it thus it would be possible to salvage the organs.

We managed to develop a new model for stepwise reduction of renal venous blood flow appropriate to our method of study and to detect renal vascular problems of relevance through early and pro-active observations.

Furthermore, this study is the first comparison of the iD-probe and microdialysis for detection of partial renal vein occlusions. The implantable Doppler probe could not detect flow changes earlier than microdialysis detected metabolic changes, and the iD-probe could not detect flow changes before there was a full stop of flow.

Based on these results, the Implantable Doppler probe cannot be recommended for early detection of vascular complications after renal transplantation, without further modifications being made to the iD-probe.

## Supporting information

S1 Data All experimental data are available in the supporting information file. (ZIP)
