# Supplementary material for: The unsuitability of implantable Doppler probes for the early detection of renal vascular complications – a porcine model for prevention of renal transplant loss
Source: PLoS One. 2017 May 25;12(5):e0178301. doi: 10.1371/journal.pone.0178301 (PMC5444816; doi:10.1371/journal.pone.0178301)

Skejby Sygehus

Urinvejskirurgisk afdeling K

Surgeon:

Operation Date: 20-08-2013 11:13:52

Patient Name: gris 15 art kontrol 2

Comments:

Patient ID:

Birthdate: Gender:

Height: Weight:

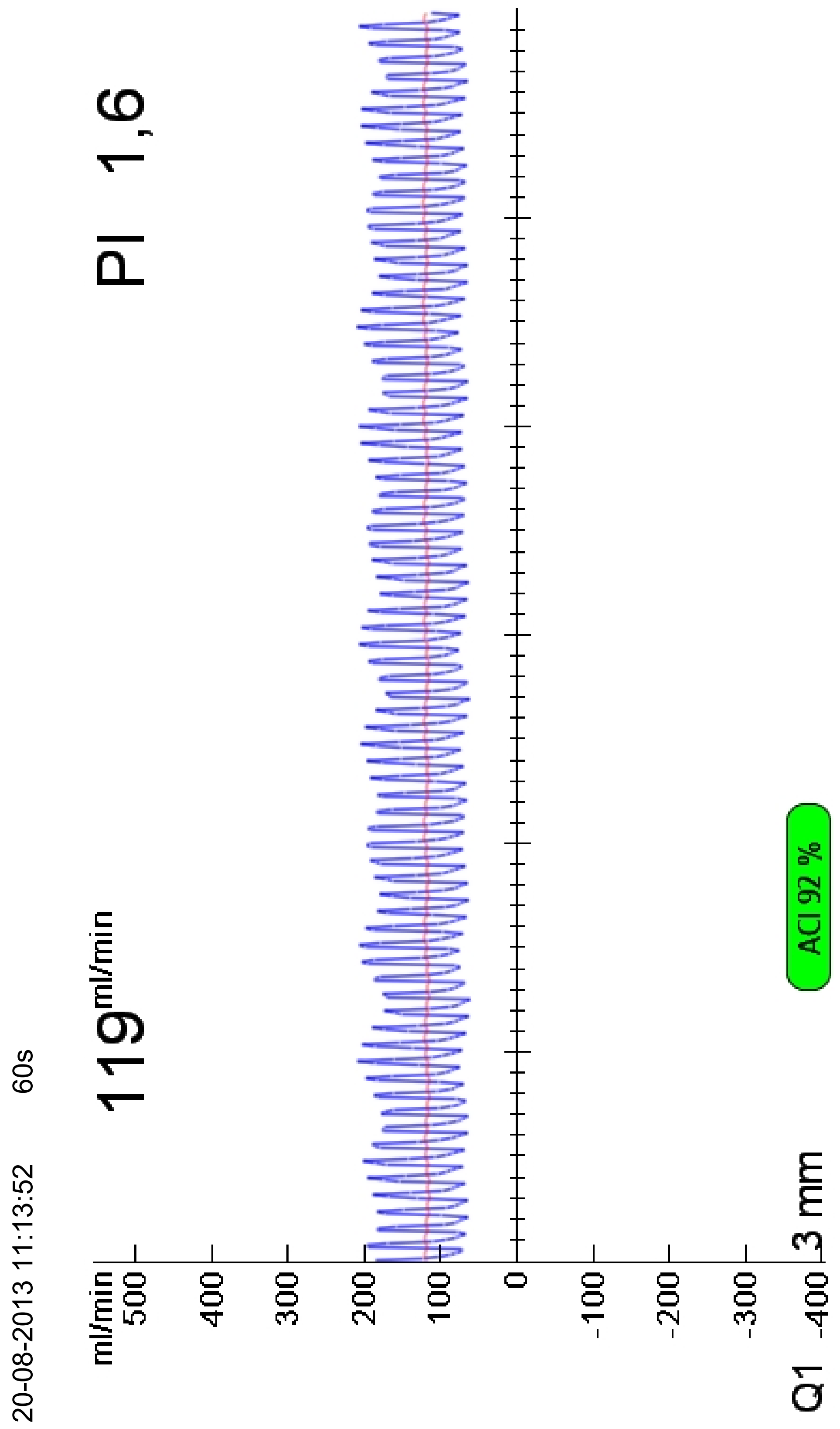

Skejby Sygehus

Urinvejskirurgisk afdeling K

Surgeon:

Operation Date: 20-08-2013 11:13:52

Patient Name: gris 15 art kontrol 2

Patient ID:

Birthdate:

Gender:

Height:

Weight:

Comments:

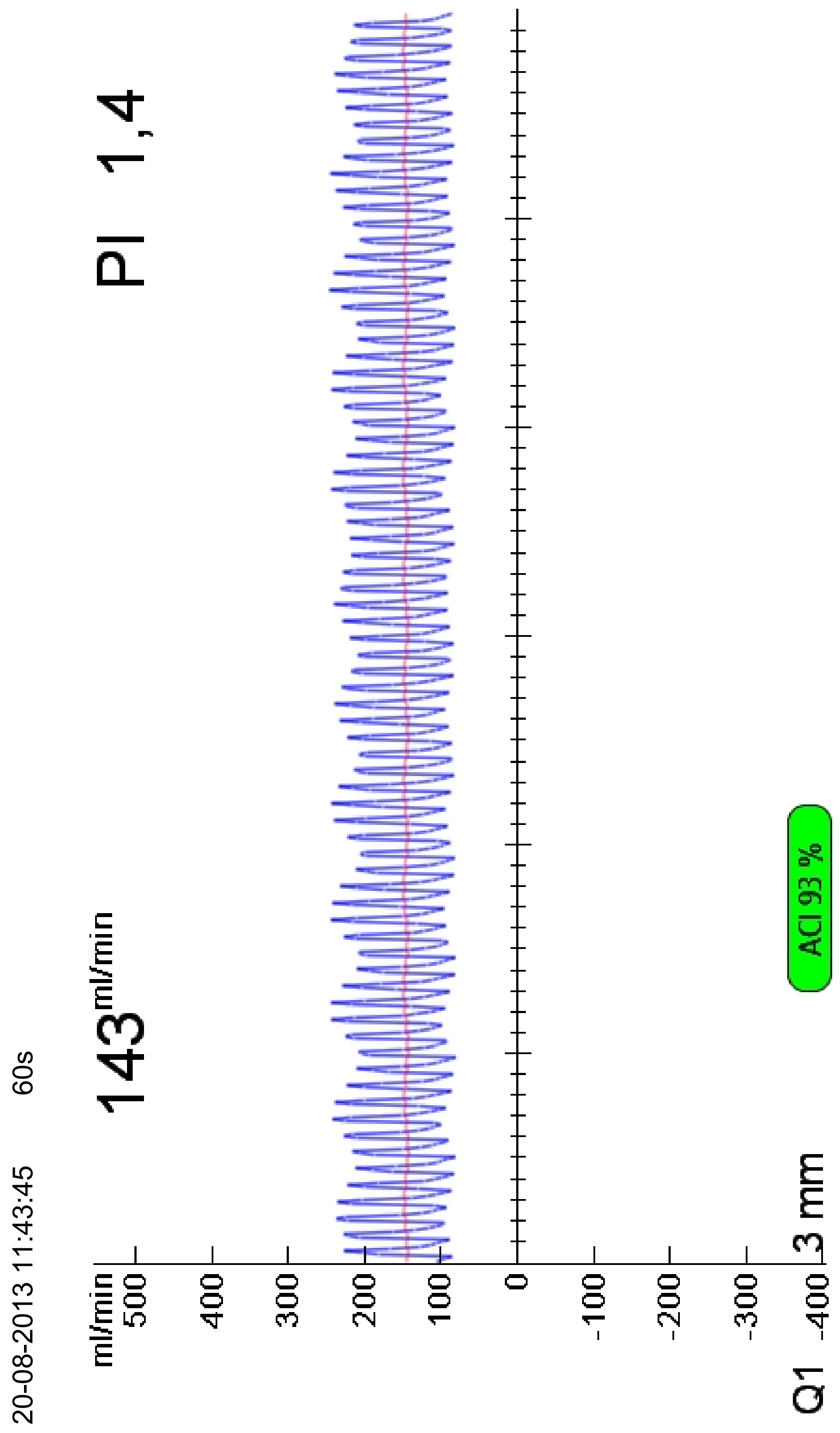

Skejby Sygehus

Urinvejskirurgisk afdeling K

Surgeon:

Operation Date: 20-08-2013 11:13:52

Patient Name: gris 15 art kontrol 2

Comments:

Patient ID:

Birthdate: Gender:

Height: Weight:

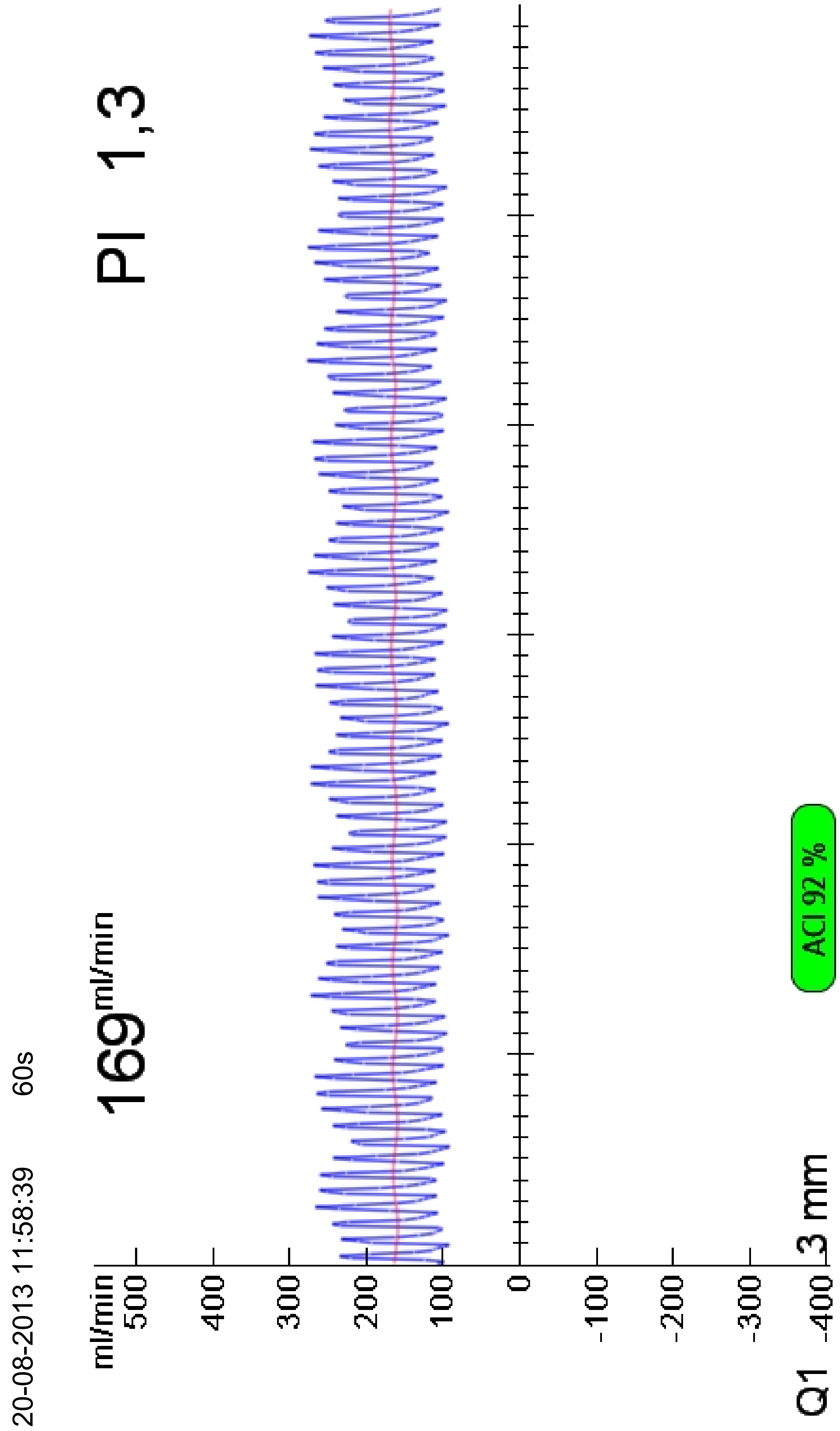

Skejby Sygehus

Urinvejskirurgisk afdeling K

Surgeon: Operation Date: 20-08-2013 11:13:52

Patient Name: gris 15 art kontrol 2

Comments:

Patient ID:

Birthdate: Gender:

Height: Weight:

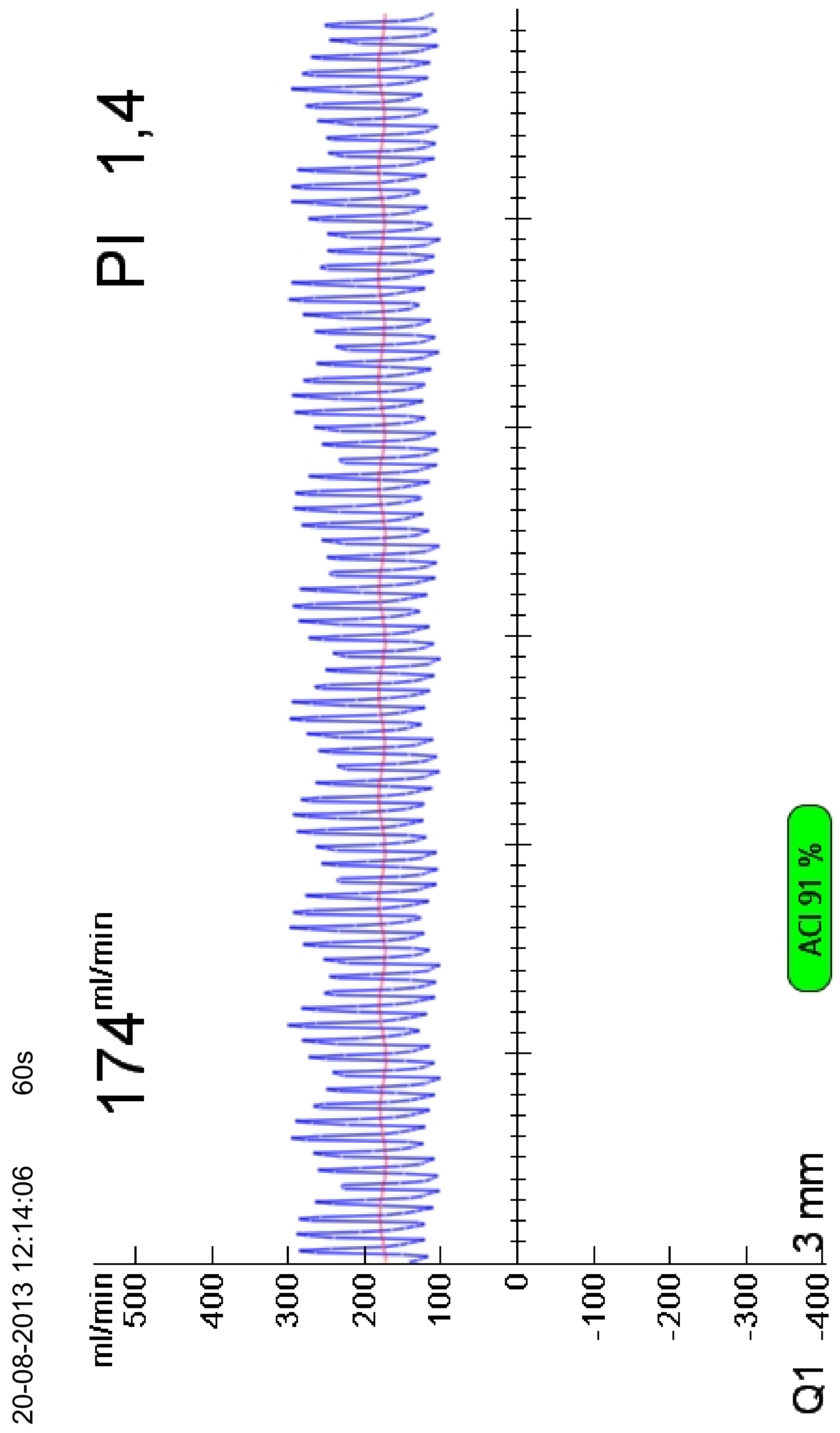

Skejby Sygehus

Urinvejskirurgisk afdeling K

Surgeon:

Operation Date: 20-08-2013 11:13:52

Patient Name: gris 15 art kontrol 2

Comments:

Patient ID:

Birthdate:

Gender:

Height:

Weight:

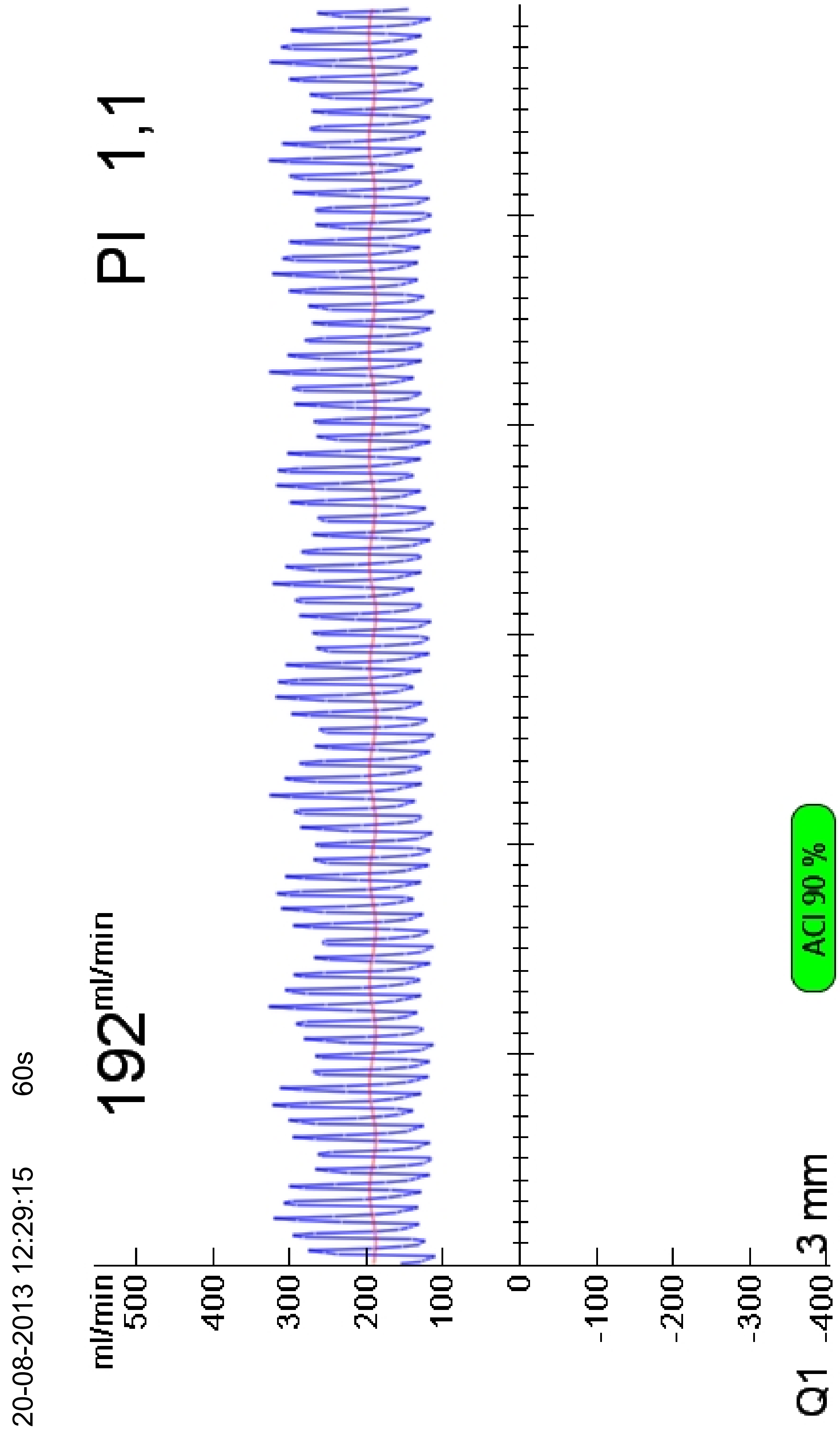

Skejby Sygehus

Urinvejskirurgisk afdeling K

Surgeon:

Operation Date: 20-08-2013 11:13:52

Patient Name: gris 15 art kontrol 2

Comments:

Patient ID:

Birthdate: Gender:

Height: Weight:

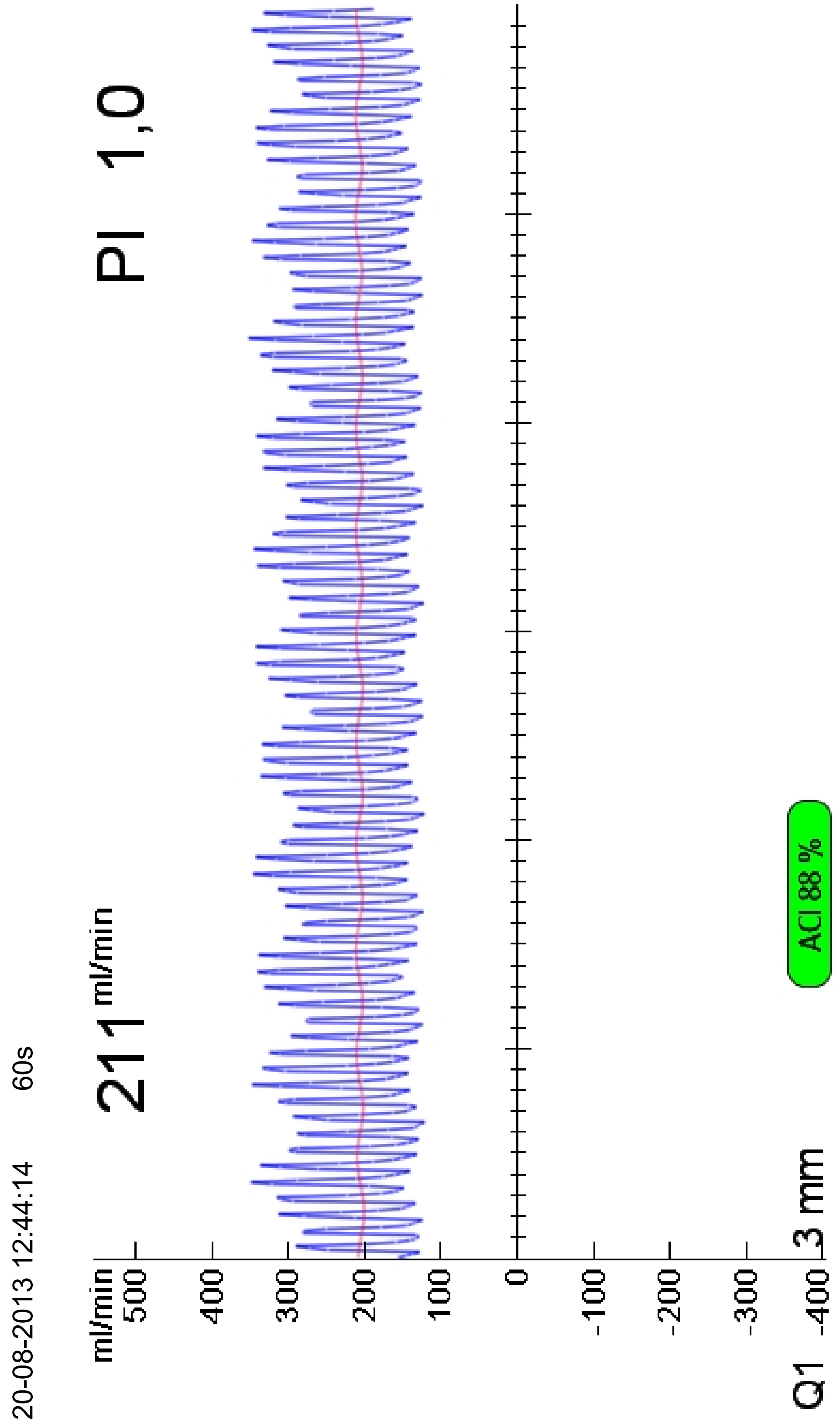

Skejby Sygehus

Urinvejskirurgisk afdeling K

Surgeon:

Operation Date: 20-08-2013 11:13:52

Patient Name: gris 15 art kontrol 2

Comments:

Patient ID:

Birthdate:

Gender:

Height:

Weight:

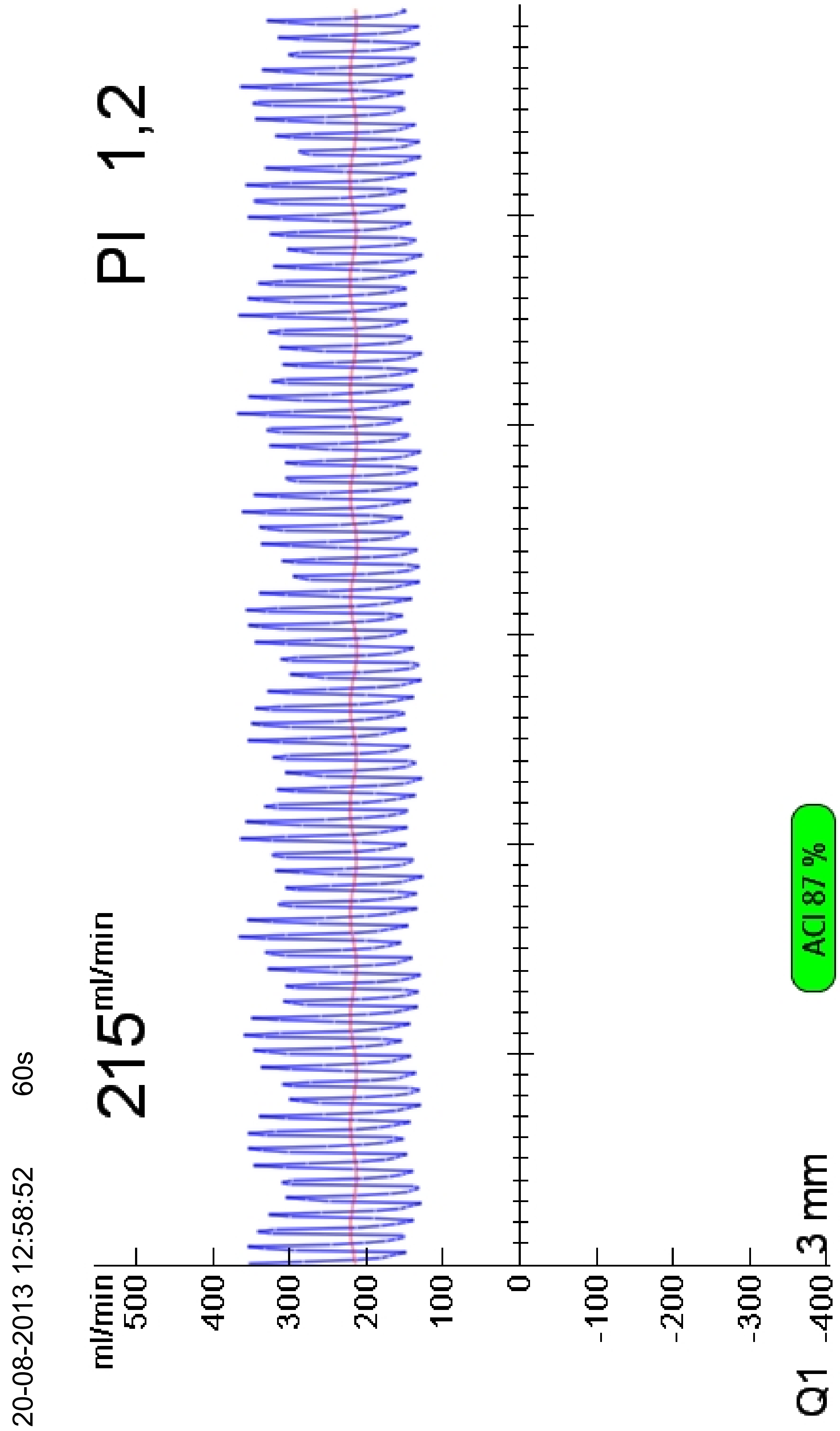

Skejby Sygehus

Urinvejskirurgisk afdeling K

MEDISTIM

Surgeon:

Operation Date: 20-08-2013 11:13:52

Patient Name: gris 15 art kontrol 2

Comments:

Patient ID:

Birthdate: Gender:

Height: Weight:

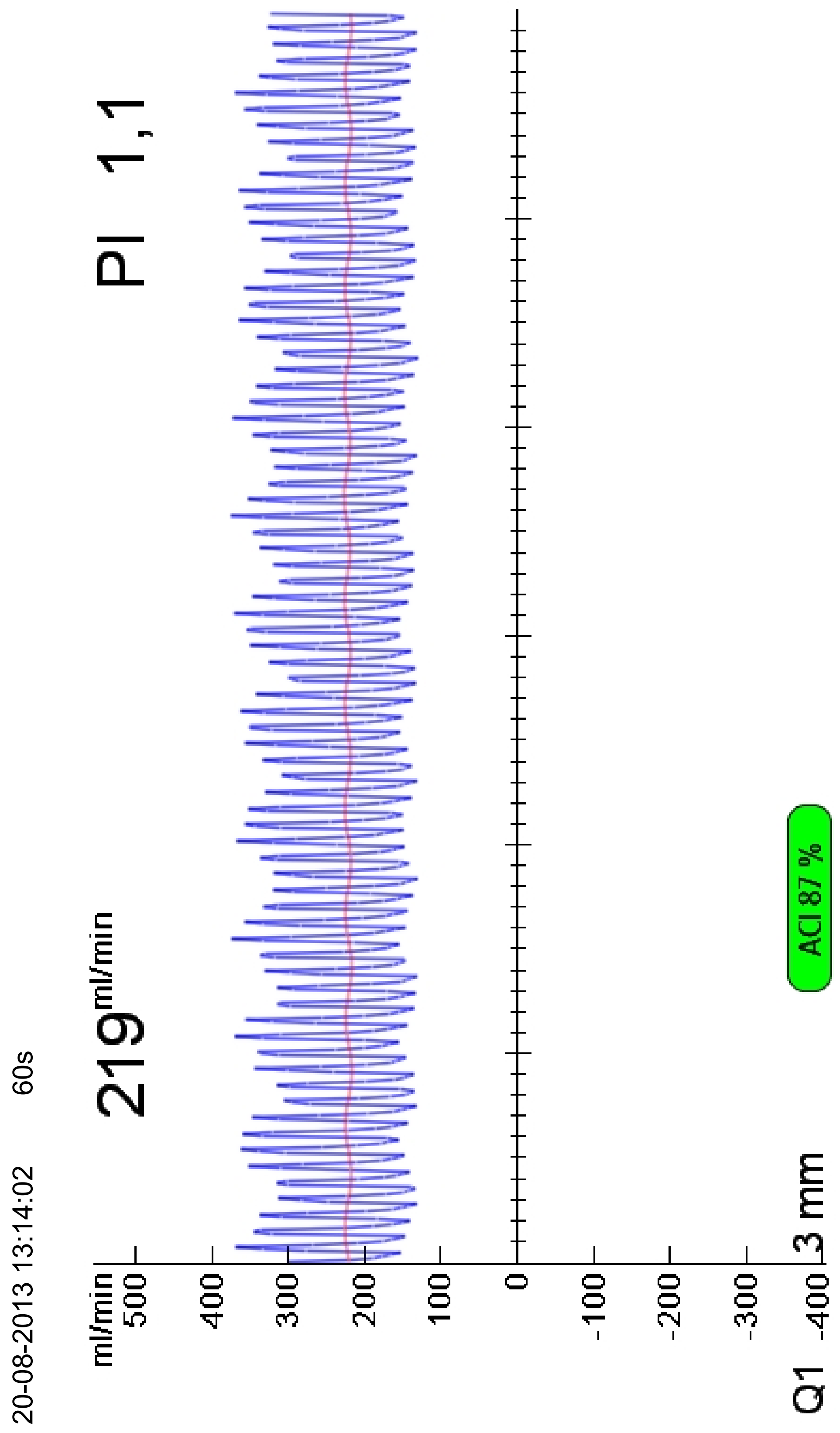

Skejby Sygehus

Urinvejskirurgisk afdeling K

Surgeon:

Operation Date: 20-08-2013 11:13:52

Patient Name: gris 15 art kontrol 2

Comments:

Patient ID:

Birthdate:

Gender:

Height:

Weight:

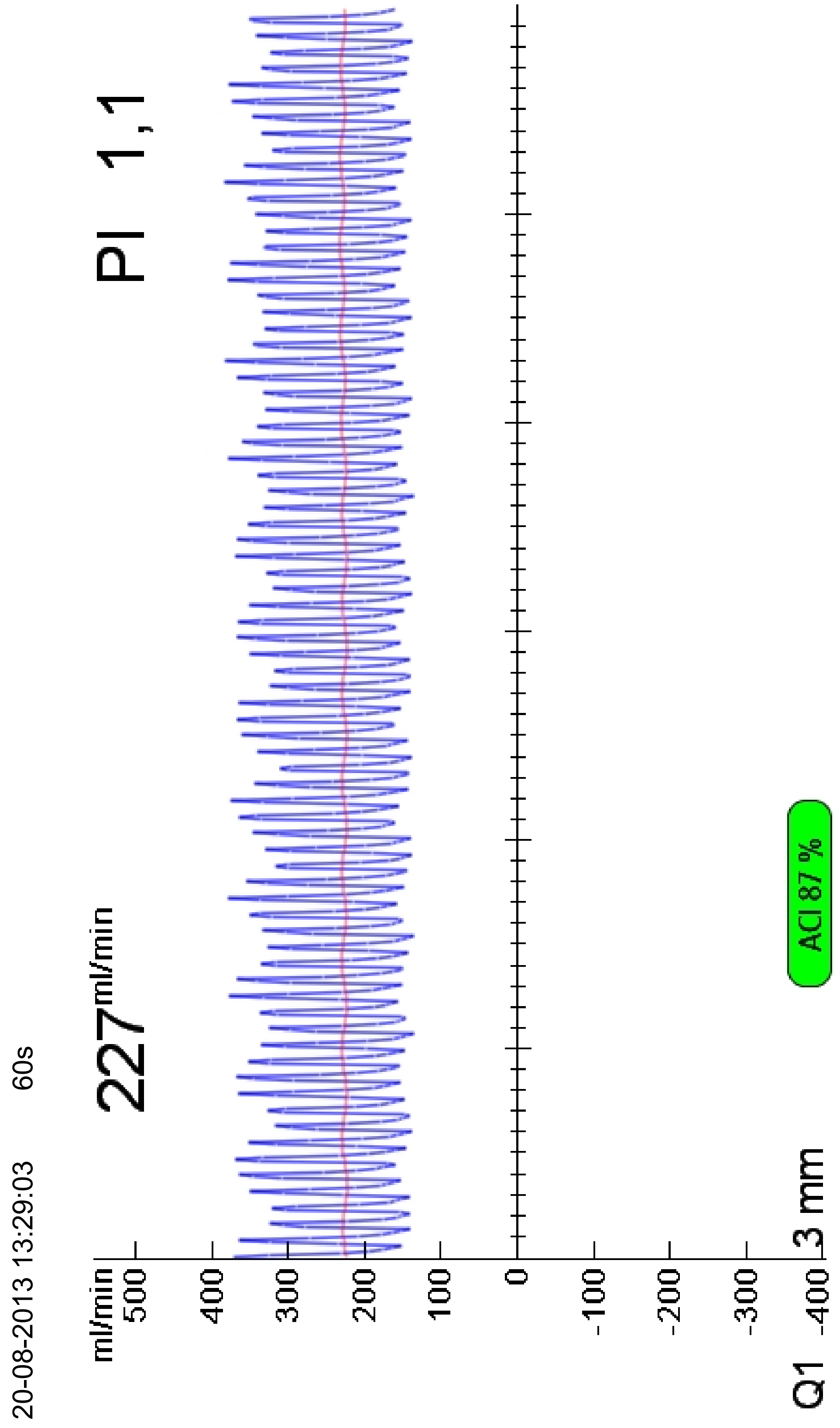

Skejby Sygehus

Urinvejskirurgisk afdeling K

Surgeon:

Operation Date: 20-08-2013 11:13:52

Patient Name: gris 15 art kontrol 2

Comments:

Patient ID:

Birthdate: Gender:

Height: Weight:

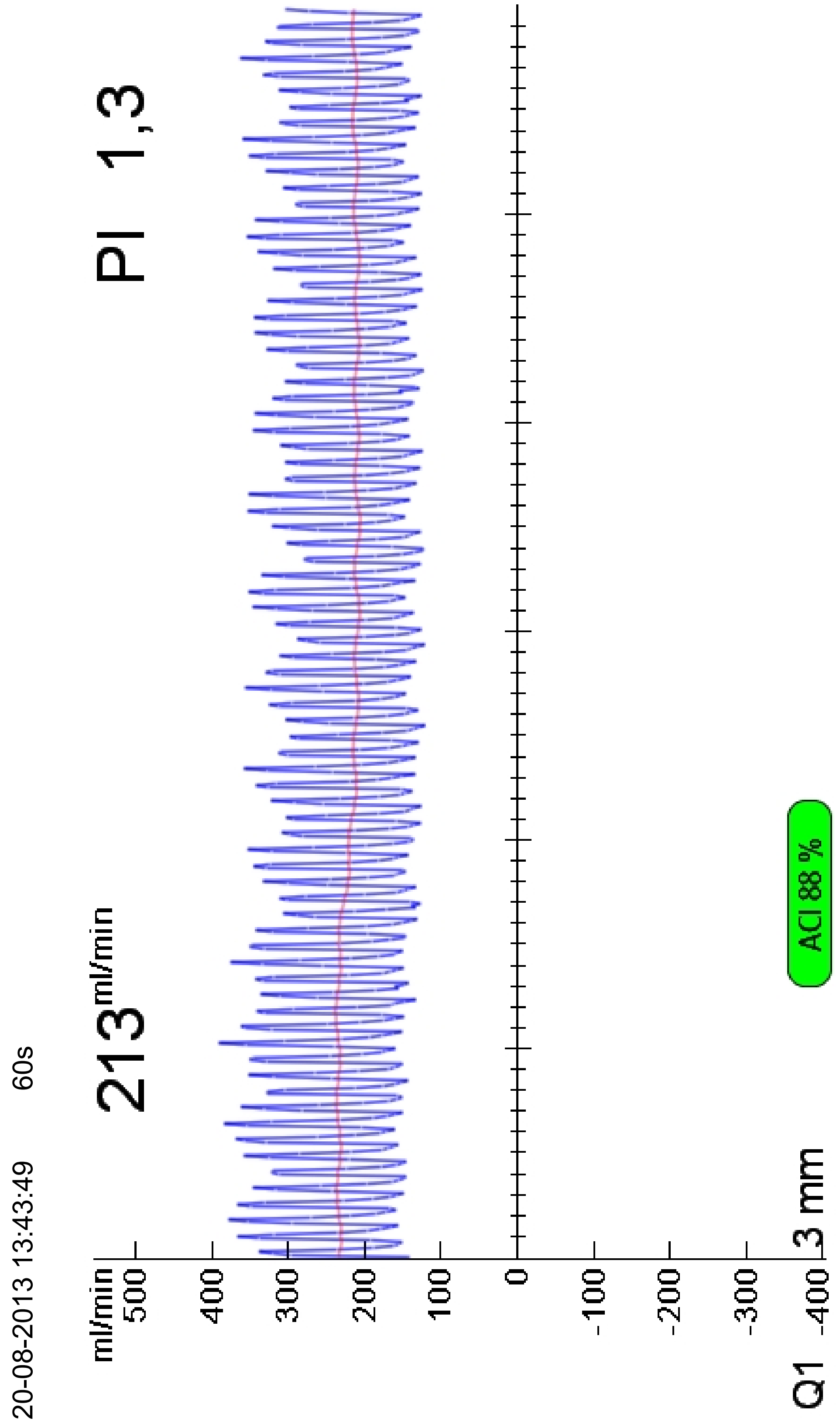

Skejby Sygehus

Urinvejskirurgisk afdeling K

Surgeon:

Operation Date: 20-08-2013 11:13:52

Patient Name: gris 15 art kontrol 2

Comments:

Patient ID:

Birthdate:

Gender:

Height:

Weight:

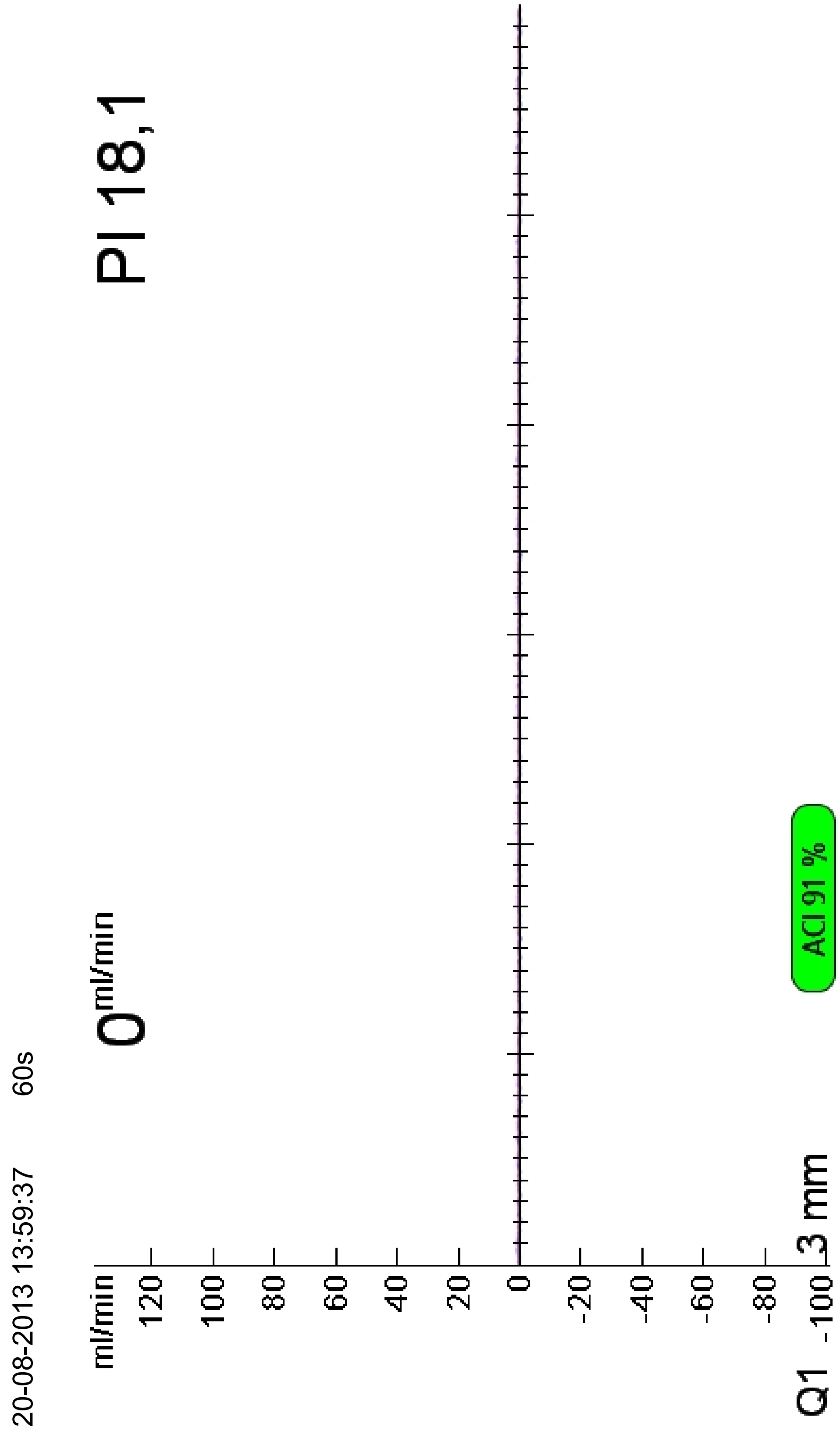

Skejby Sygehus

Urinvejskirurgisk afdeling K

Surgeon:

Operation Date: 20-08-2013 11:13:52

Patient Name: gris 15 art kontrol 2

Comments:

Patient ID:

Birthdate: Gender:

Height: Weight:

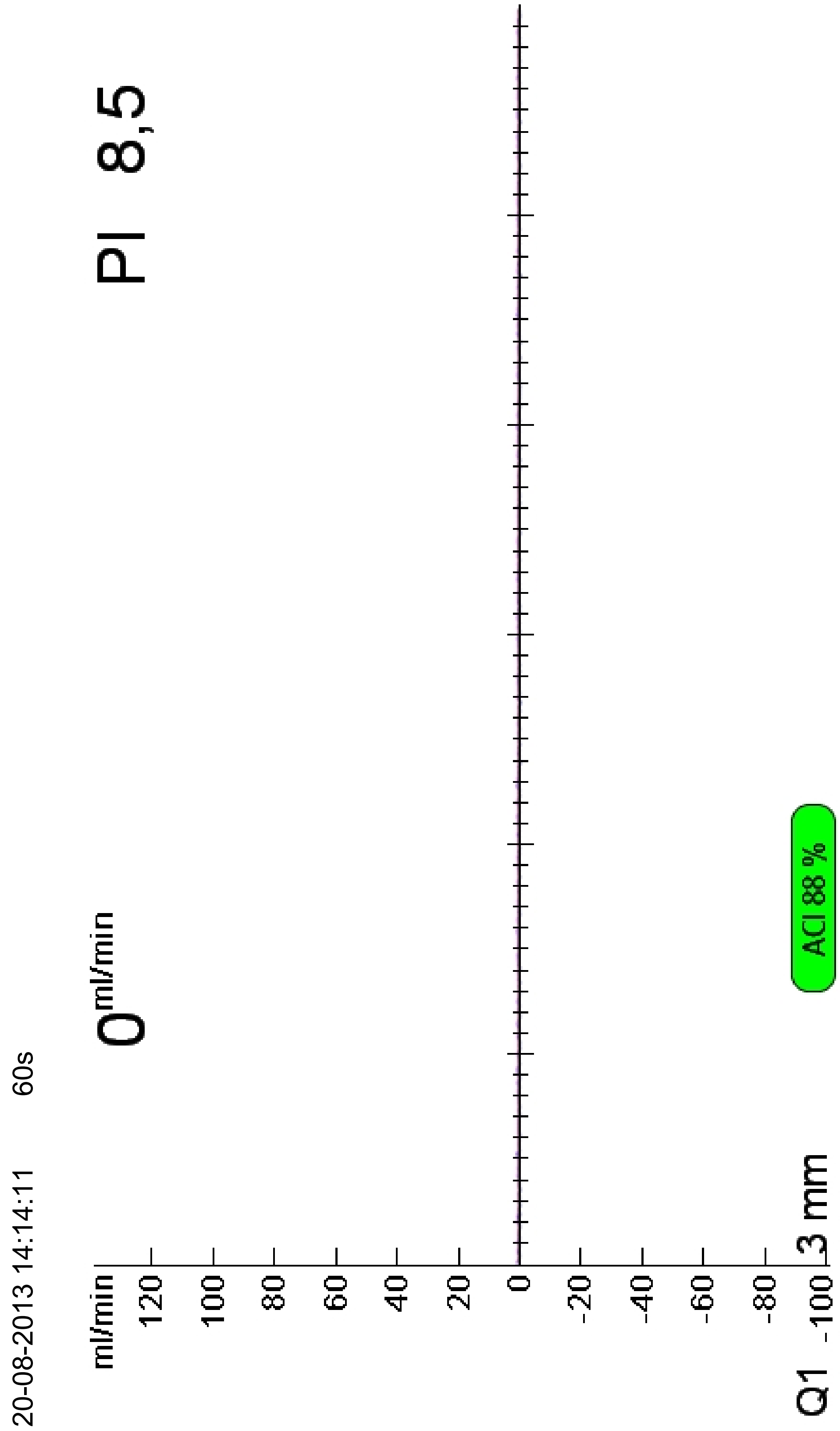

Skejby Sygehus

Urinvejskirurgisk afdeling K

Surgeon:

Operation Date: 20-08-2013 11:13:52

Patient Name: gris 15 art kontrol 2

Comments:

Patient ID:

Birthdate:

Gender:

Height:

Weight:

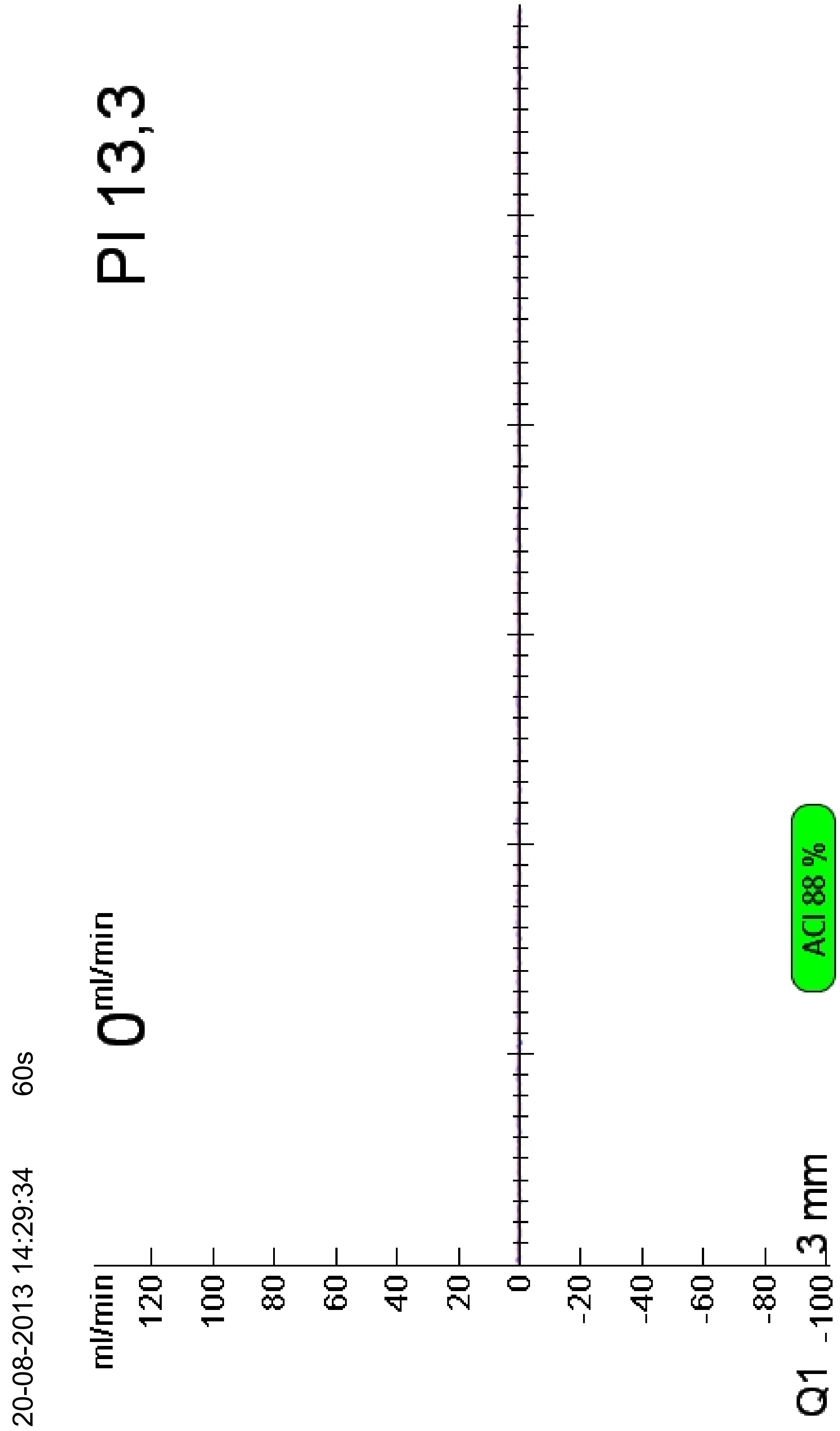

Skejby Sygehus

Urinvejskirurgisk afdeling K

Surgeon: Operation Date: 20-08-2013 11:13:52

Patient Name: gris 15 art kontrol 2

Comments:

Patient ID:

Birthdate: Gender:

Height: Weight:

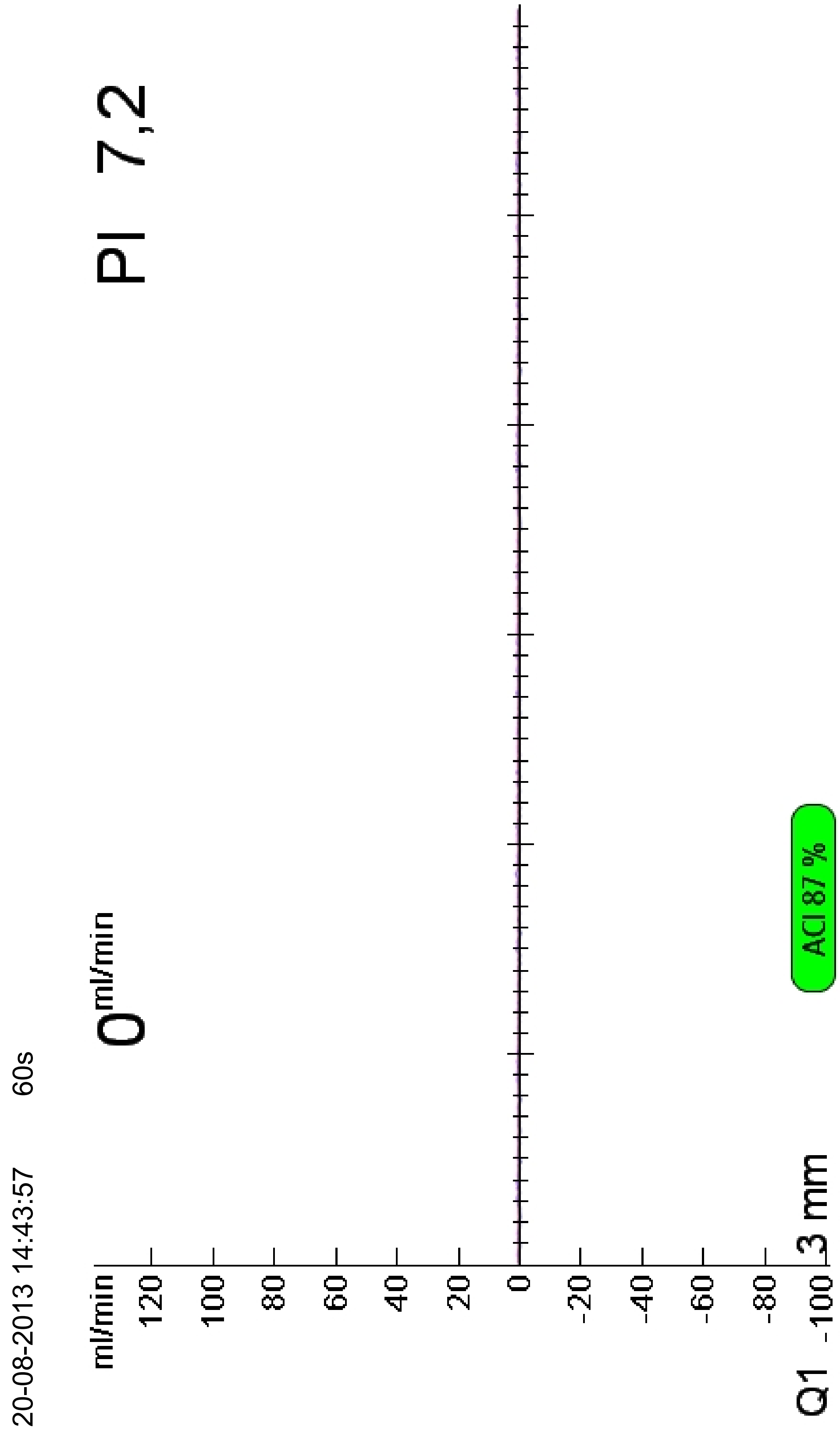

Skejby Sygehus

MEDISTIM

Urinvejskirurgisk afdeling K

Surgeon:

Operation Date: 20-08-2013 11:13:52

Patient Name: gris 15 art kontrol 2

Comments:

Patient ID:

Birthdate:

Gender:

Height:

Weight:

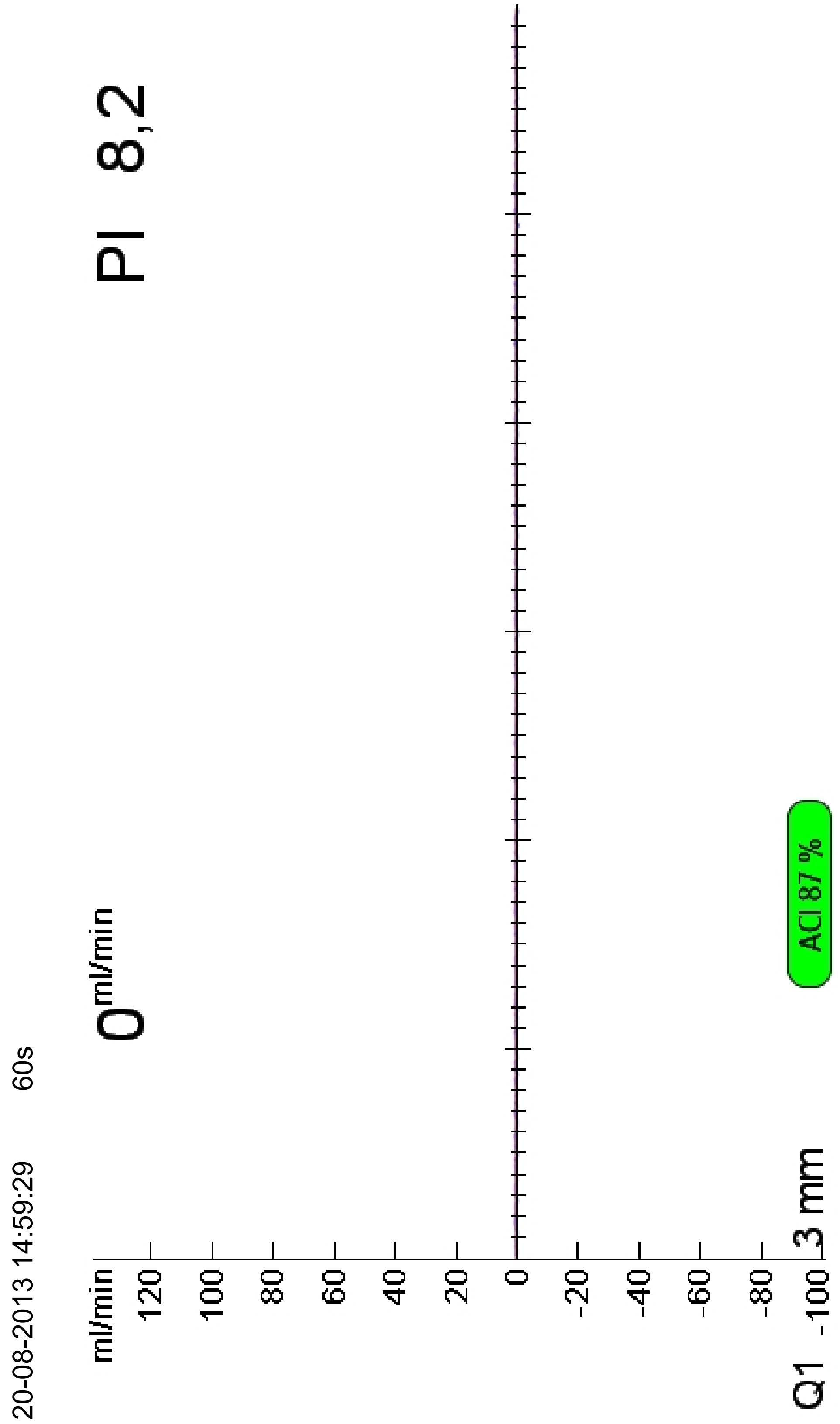

Skejby Sygehus

Urinvejskirurgisk afdeling K

MEDISTIM

Surgeon:

Operation Date: 20-08-2013 11:13:52

Patient Name: gris 15 art kontrol 2

Comments:

Patient ID:

Birthdate: Gender:

Height: Weight:

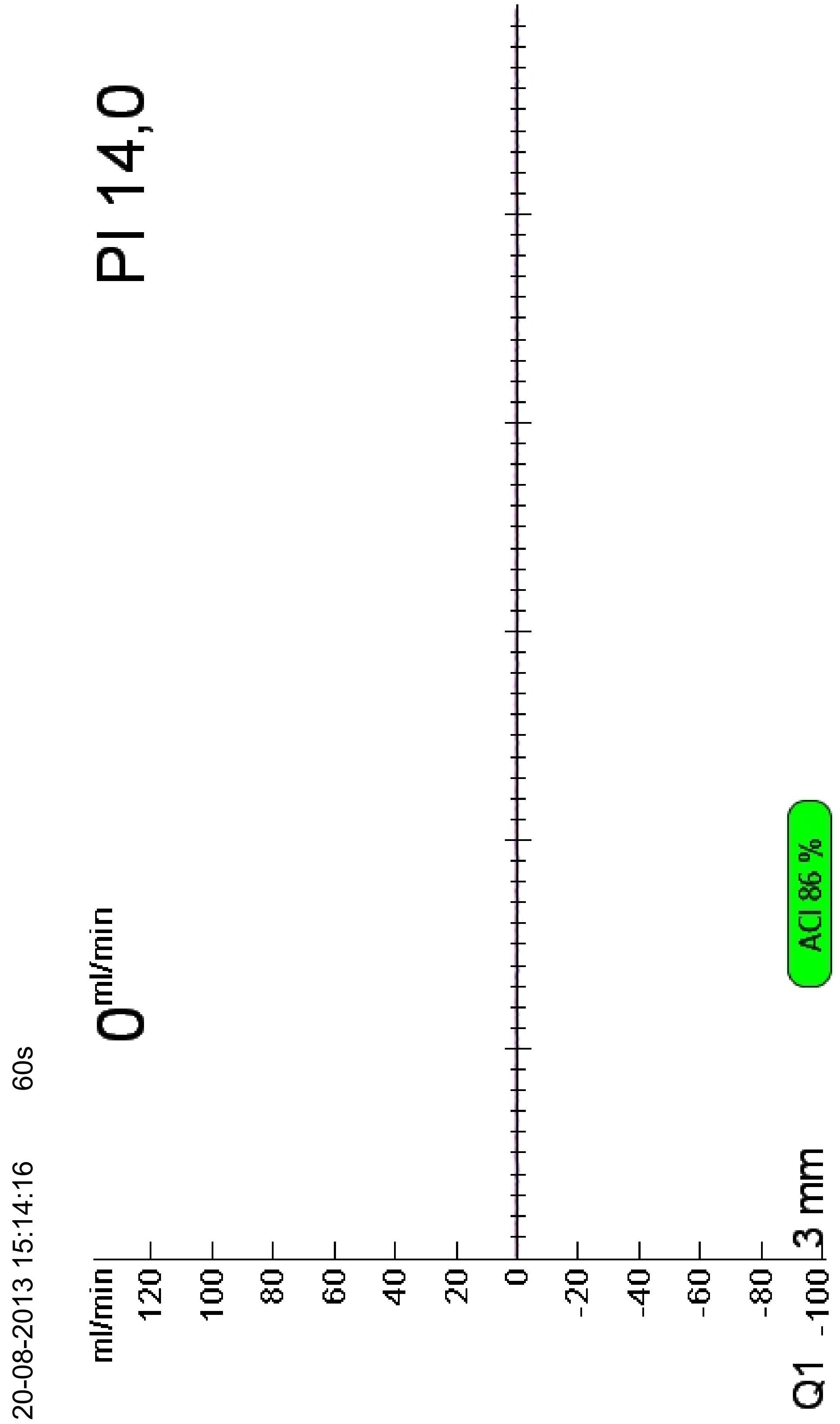

Skejby Sygehus

Urinvejskirurgisk afdeling K

MEDISTIM

Surgeon:

Operation Date: 20-08-2013 11:13:52

Patient Name: gris 15 art kontrol 2

Comments:

Patient ID:

Birthdate: Gender:

Height: Weight:

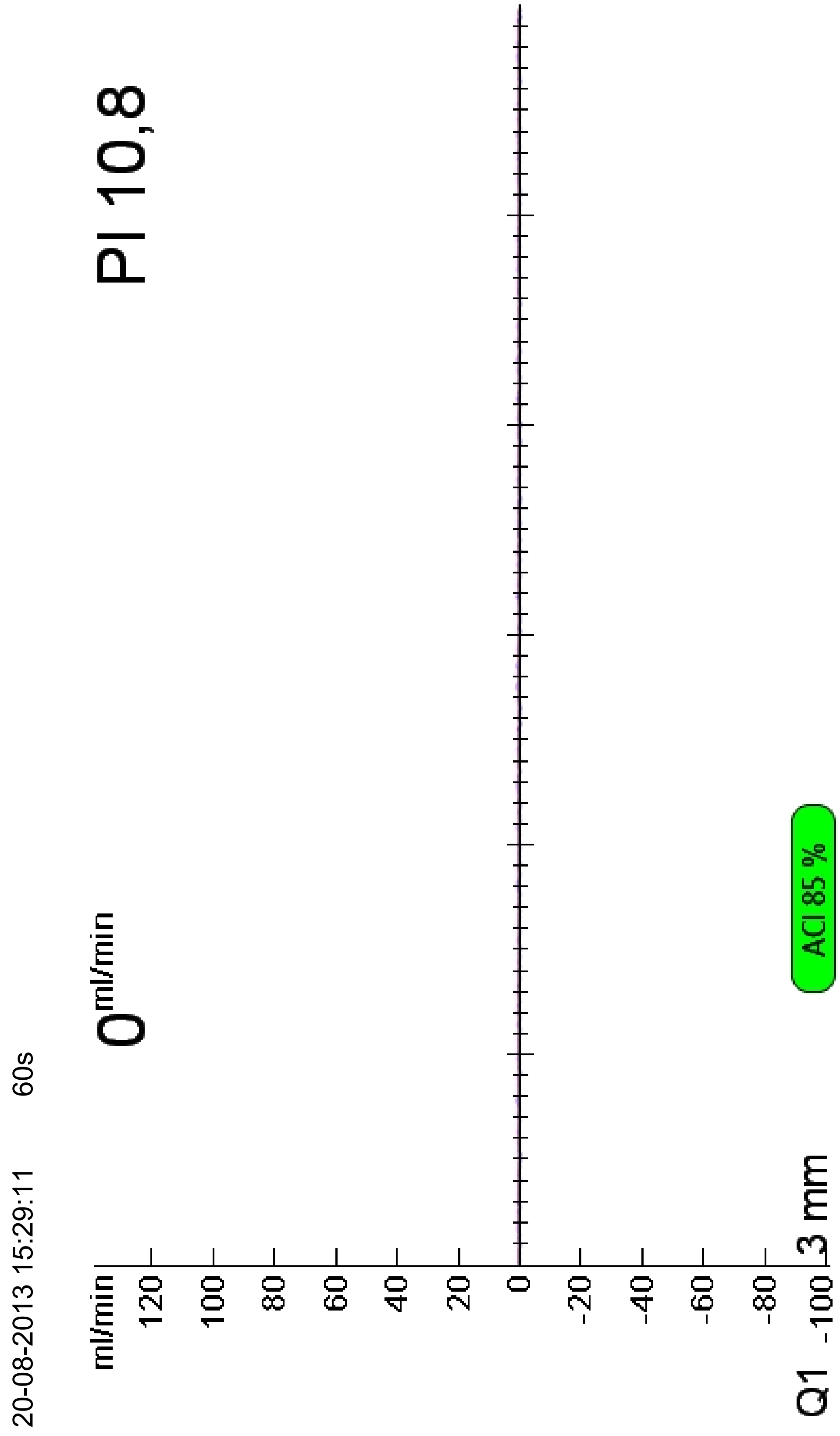

Skejby Sygehus

Urinvejskirurgisk afdeling K

Surgeon:

Operation Date: 20-08-2013 11:13:52

Patient Name: gris 15 art kontrol 2

Patient ID:

Birthdate:

Gender:

Height:

Weight:

Comments:

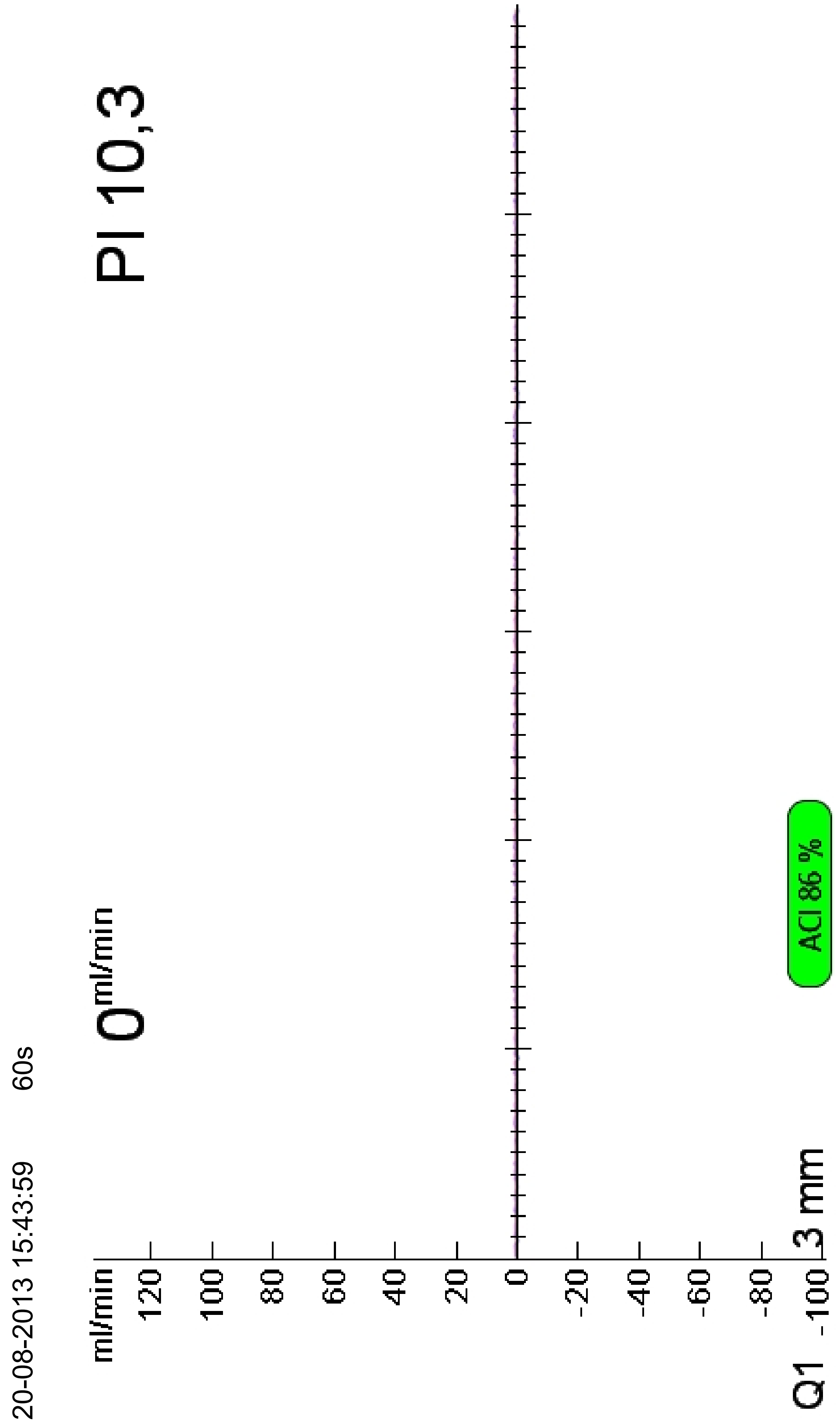

Patient Report

Skejby Sygehus

Urinvejskirurgisk afdeling K

Surgeon:

Operation Date: 20-08-2013 11:13:52

Patient Name: gris 15 art kontrol 2

Comments:

Patient ID:

Birthdate: Gender:

Height: Weight:

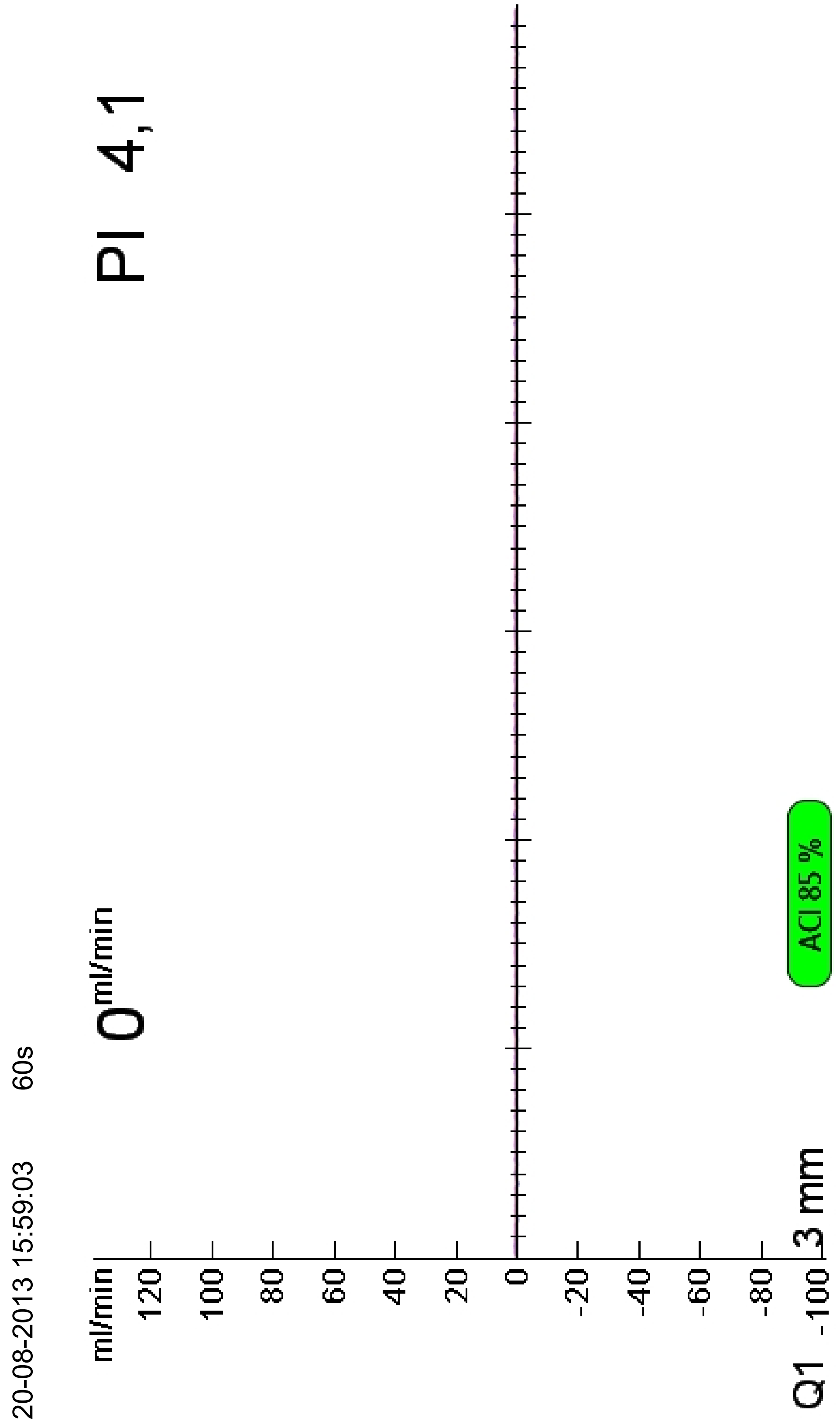

Skejby Sygehus

Urinvejskirurgisk afdeling K

Surgeon: Operation Date: 20-08-2013 11:13:52

Patient Name: gris 15 art kontrol 2

Comments:

Patient ID:

Birthdate: Gender:

Height: Weight:

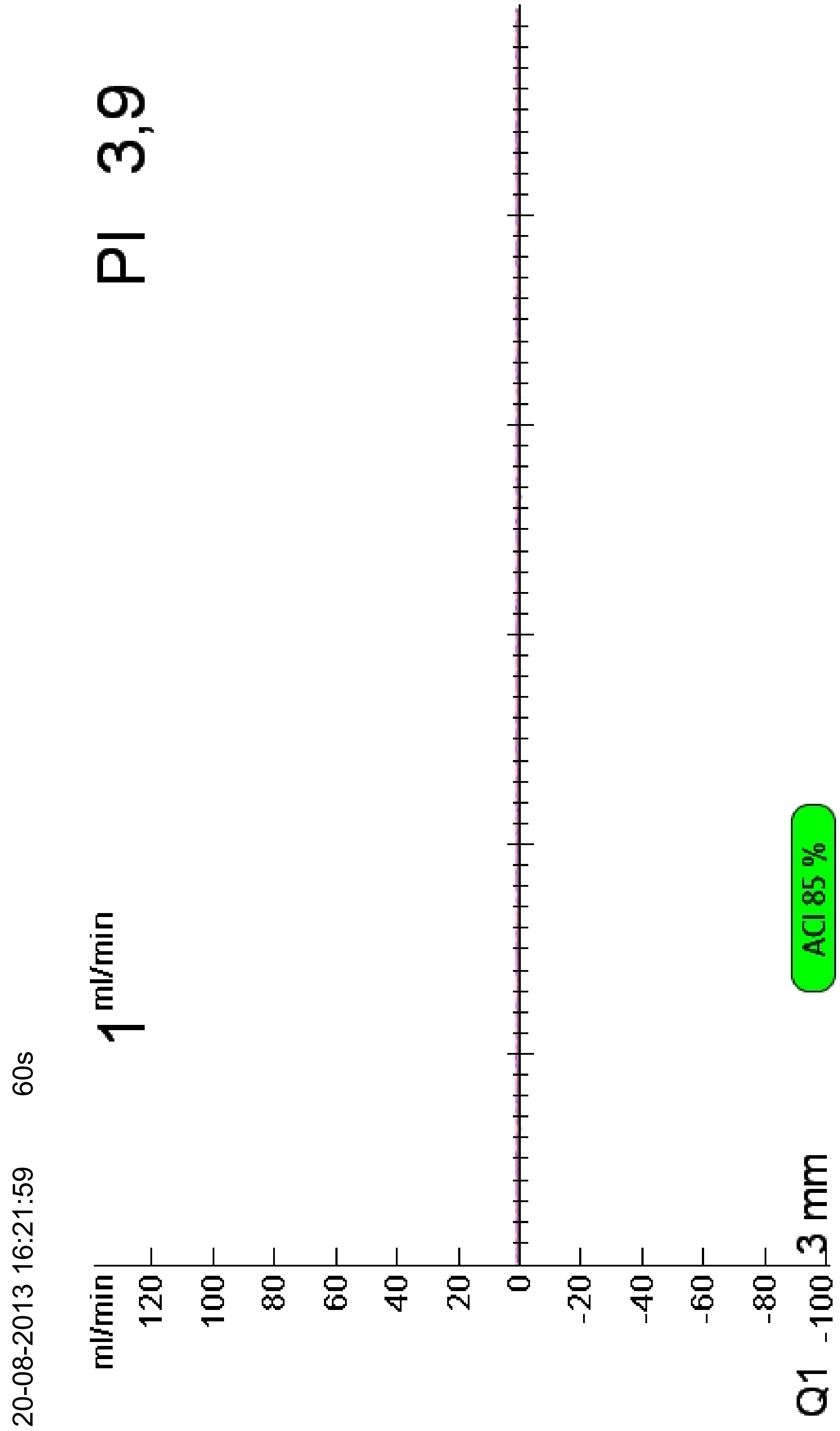

Skejby Sygehus

MEDISTIM

Urinvejskirurgisk afdeling K

Surgeon:

Operation Date: 20-08-2013 11:13:52

Patient Name: gris 15 art kontrol 2

Comments:

Patient ID:

Birthdate: Gender:

Height: Weight:

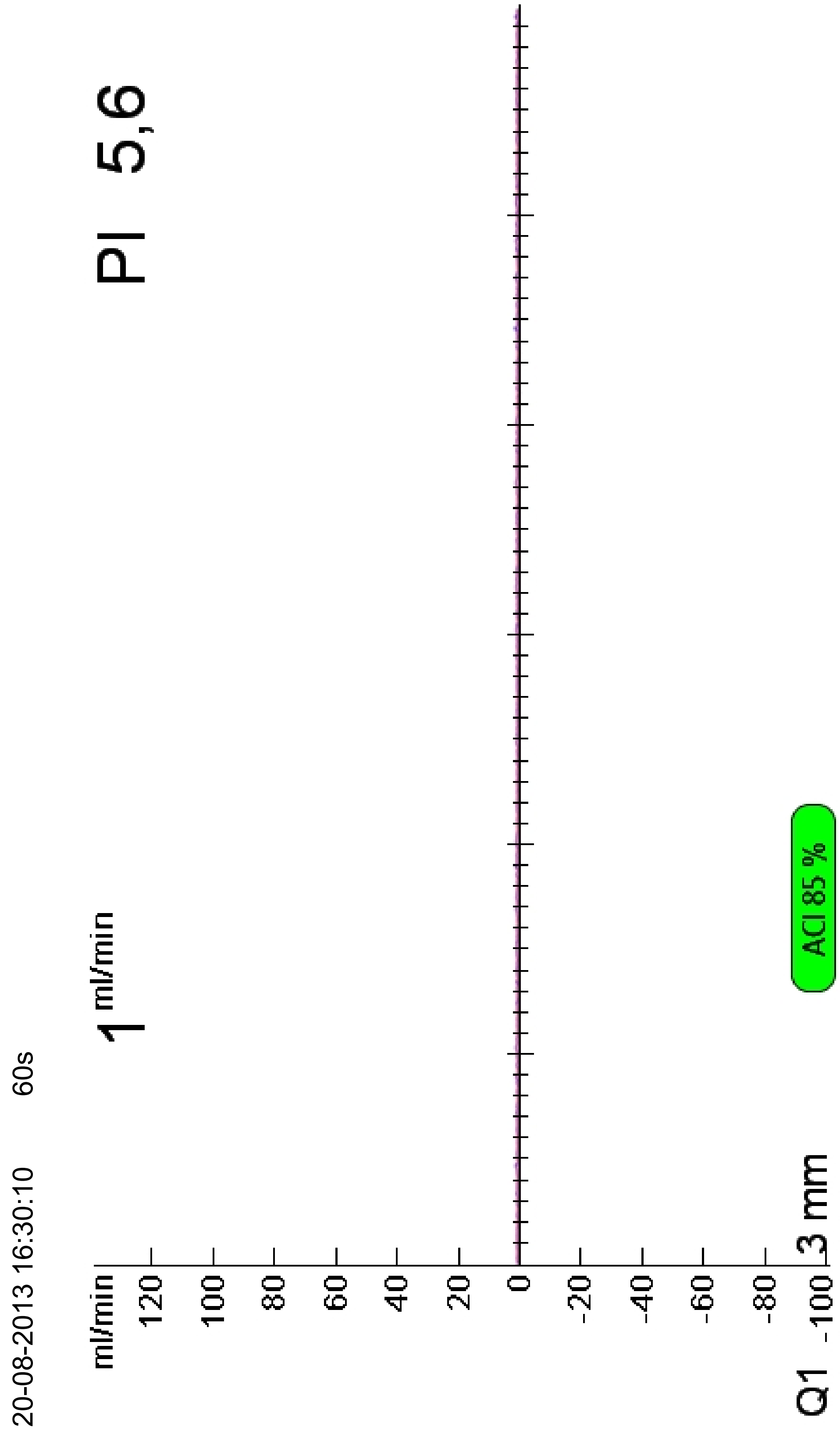

Skejby Sygehus

Urinvejskirurgisk afdeling K

Surgeon:

Operation Date: 20-08-2013 11:13:52

Patient Name: gris 15 art kontrol 2

Comments:

Patient ID:

Birthdate: Gender:

Height: Weight:

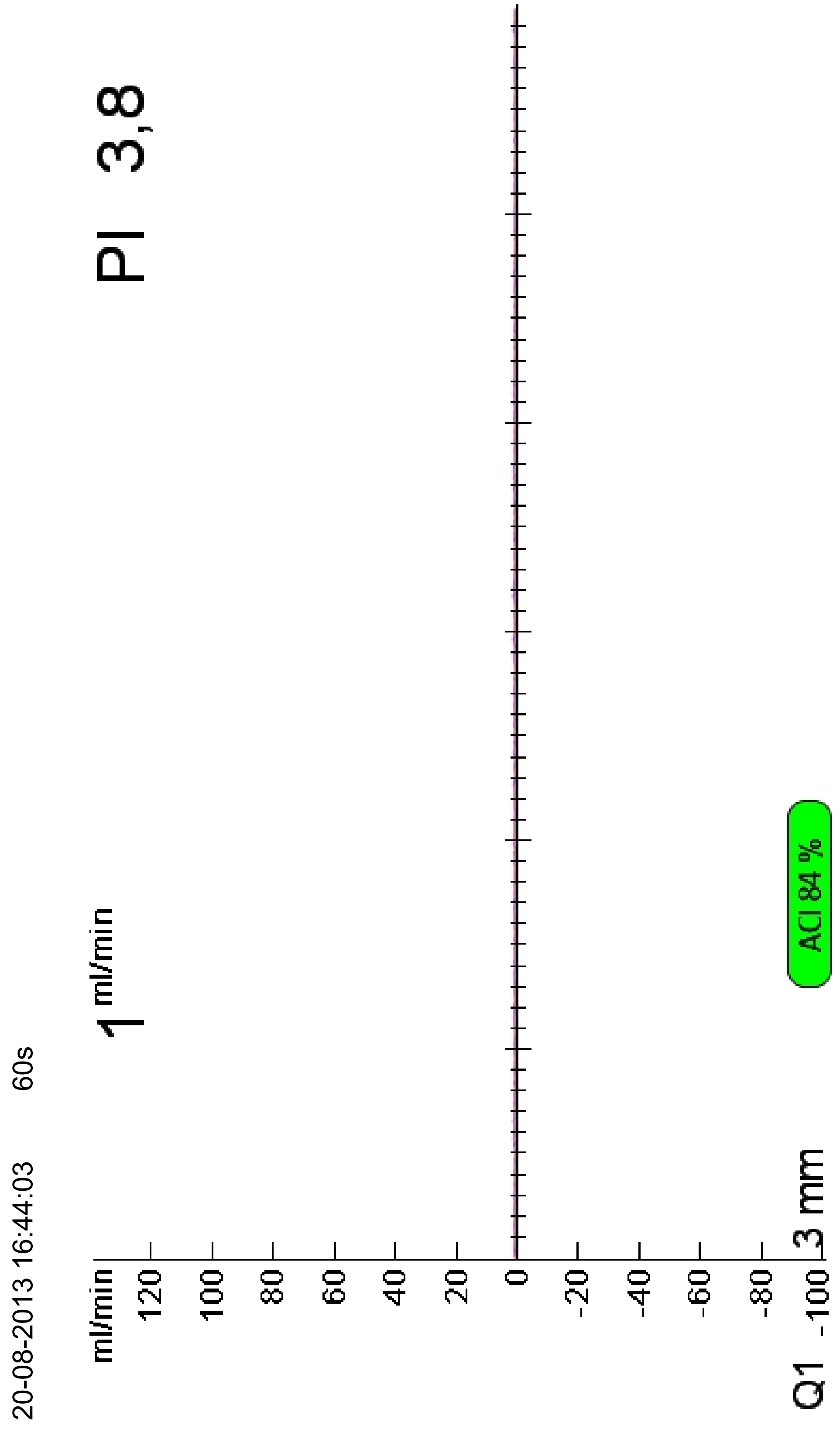

Supplement: S1 Data — (ZIP) [file pone.0178301.s001.zip › Supporting Information/Art. 2 d. 20.08.13/gris 15 art kontrol 2.pdf]
