# Supplementary material for: The unsuitability of implantable Doppler probes for the early detection of renal vascular complications – a porcine model for prevention of renal transplant loss
Source: PLoS One. 2017 May 25;12(5):e0178301. doi: 10.1371/journal.pone.0178301 (PMC5444816; doi:10.1371/journal.pone.0178301)
Supplement: S1 Data — (ZIP) [file pone.0178301.s001.zip › Supporting Information/Kontrol 1 d. 03.06.13/Cook-problemer.docx]

Cook-problemer d. 03.06.2013

3 arterier med en sen samling.

Medistimproben beskriver flow på ca. 100 ml/min.

Cook-proben giver intet signal på arterien. Der forsøges først med to brugte prober, der begge giver signal på venen.

Derefter blev der forsøgt med en ny probe. Den gav også signal på venen, men intet signal fra arterien.

Enden blev, at cookproben blev sat på arterien, uden signal, trods pænt flow.
