# Supplementary material for: The unsuitability of implantable Doppler probes for the early detection of renal vascular complications – a porcine model for prevention of renal transplant loss
Source: PLoS One. 2017 May 25;12(5):e0178301. doi: 10.1371/journal.pone.0178301 (PMC5444816; doi:10.1371/journal.pone.0178301)

Skejby Sygehus

Urinvejskirurgisk afdeling K

Surgeon:

Operation Date: 31-07-2013 13:44:55

Patient Name: chris gris 10

Comments:

Patient ID:

Birthdate:

Gender:

Height:

Weight:

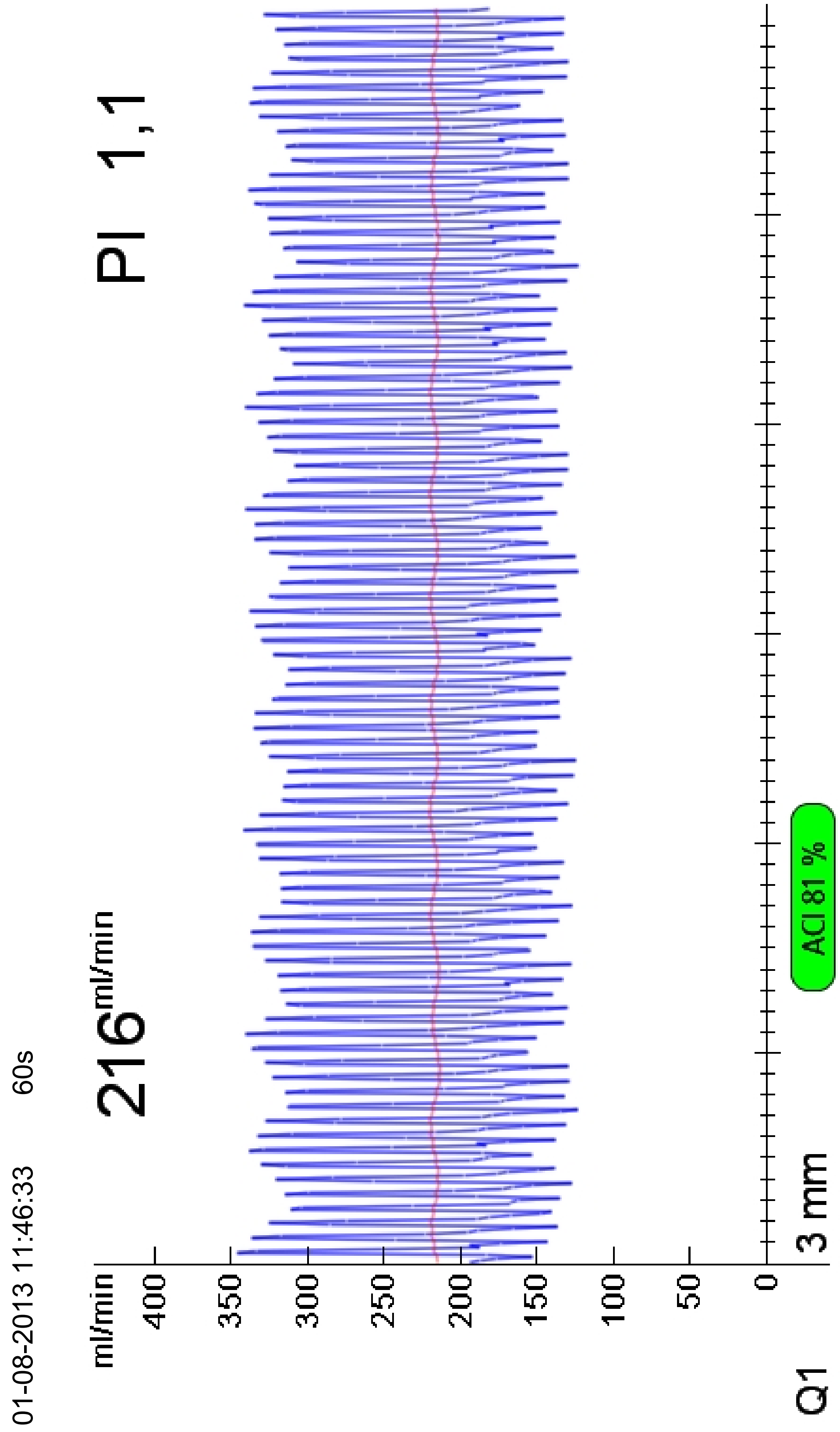

Skejby Sygehus

Urinvejskirurgisk afdeling K

MEDISTIM

Surgeon:

Operation Date: 31-07-2013 13:44:55

Patient Name: chris gris 10

Comments:

Patient ID:

Birthdate: Gender:

Height: Weight:

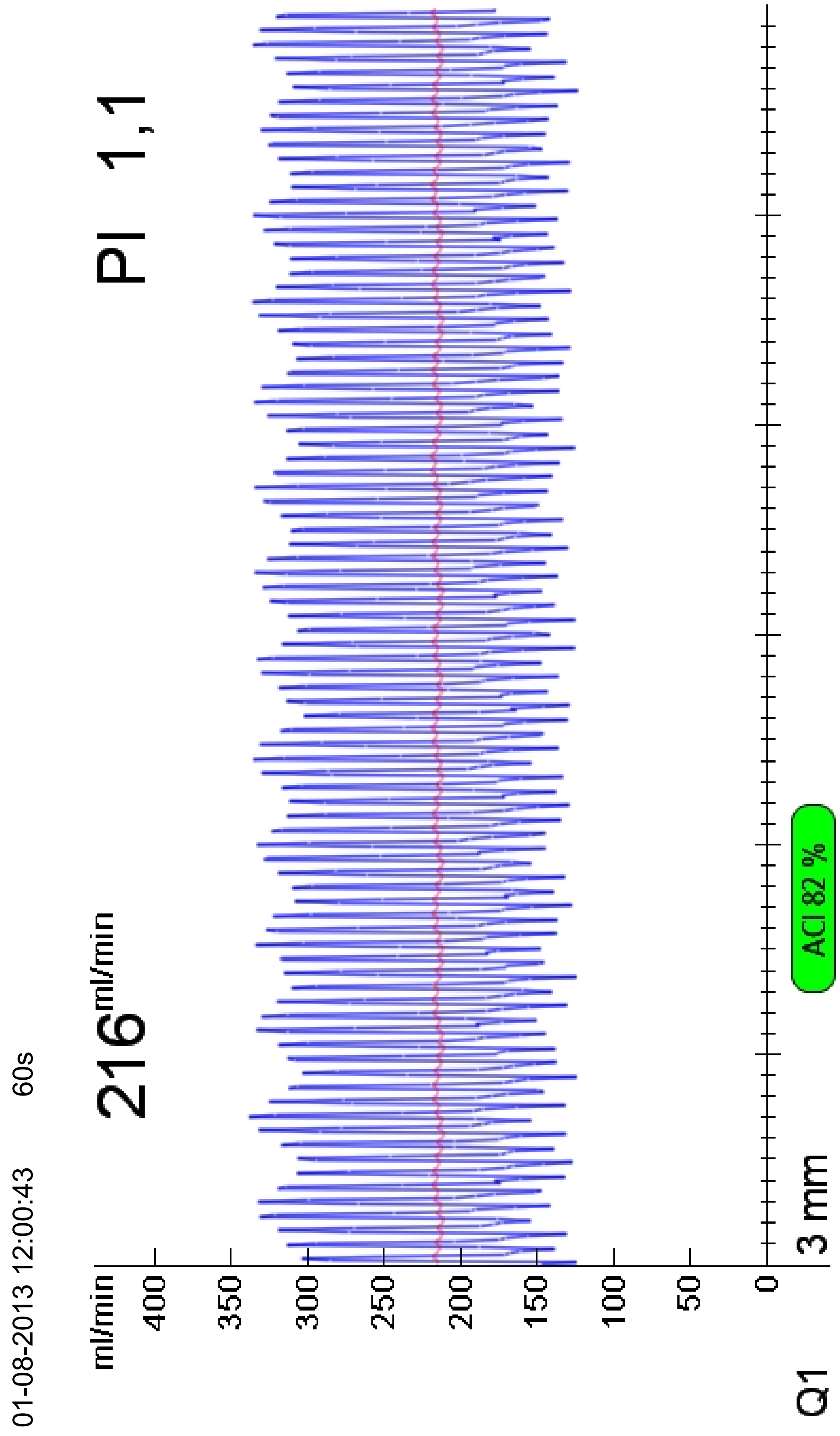

Skejby Sygehus

Urinvejskirurgisk afdeling K

MEDISTIM

Surgeon:

Operation Date: 31-07-2013 13:44:55

Patient Name: chris gris 10

Comments:

Patient ID:

Birthdate:

Gender:

Height:

Weight:

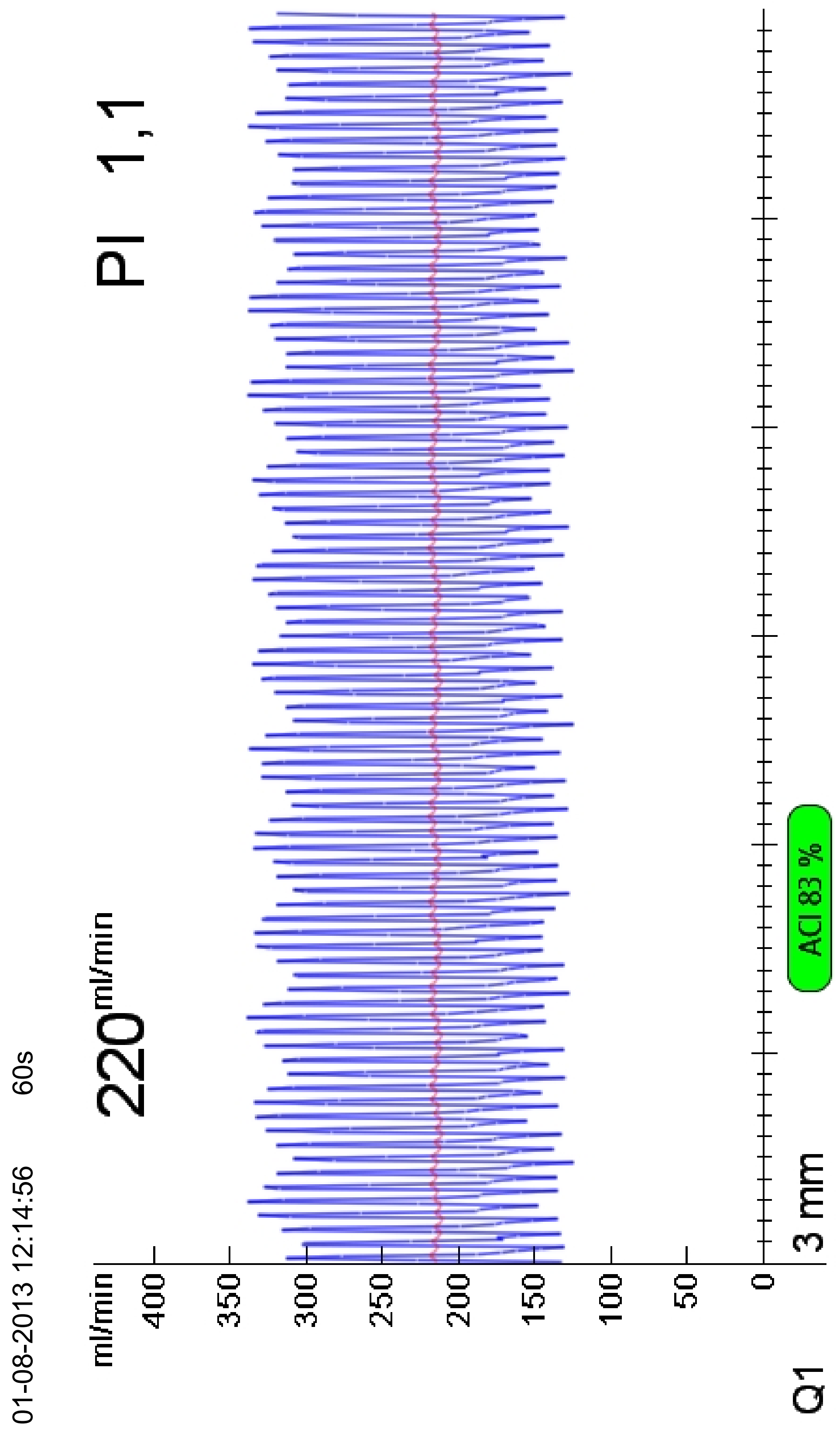

Skejby Sygehus

Urinvejskirurgisk afdeling K

Surgeon:

Operation Date: 31-07-2013 13:44:55

Patient Name: chris gris 10

Comments:

Patient ID:

Birthdate:

Gender:

Height:

Weight:

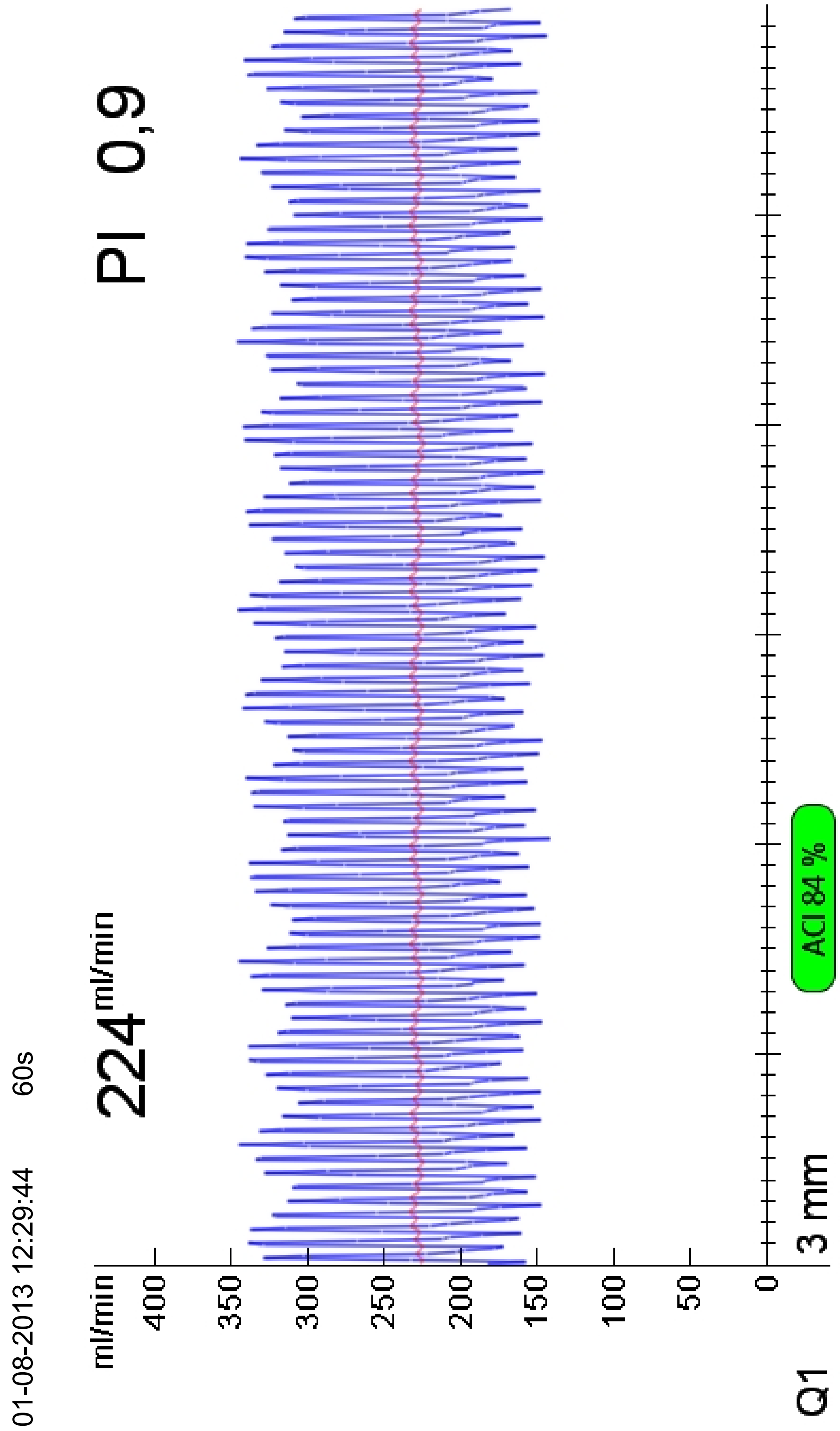

Skejby Sygehus

Urinvejskirurgisk afdeling K

Surgeon:

Operation Date: 31-07-2013 13:44:55

Patient Name: chris gris 10

Patient ID:

Birthdate:

Gender:

Height:

Weight:

Comments:

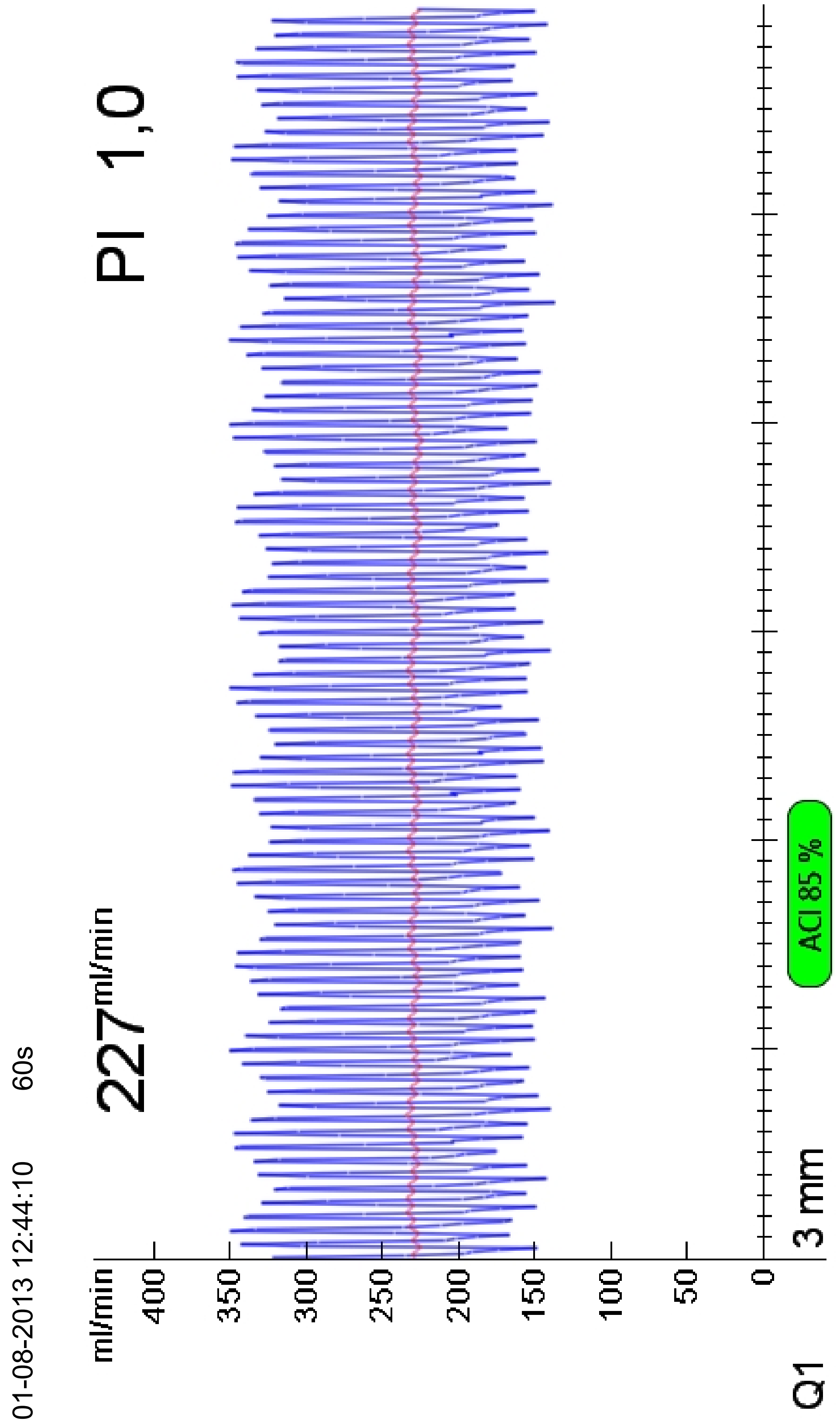

Skejby Sygehus

Urinvejskirurgisk afdeling K

MEDISTIM

Surgeon:

Operation Date: 31-07-2013 13:44:55

Patient Name: chris gris 10

Comments:

Patient ID:

Birthdate:

Gender:

Height:

Weight:

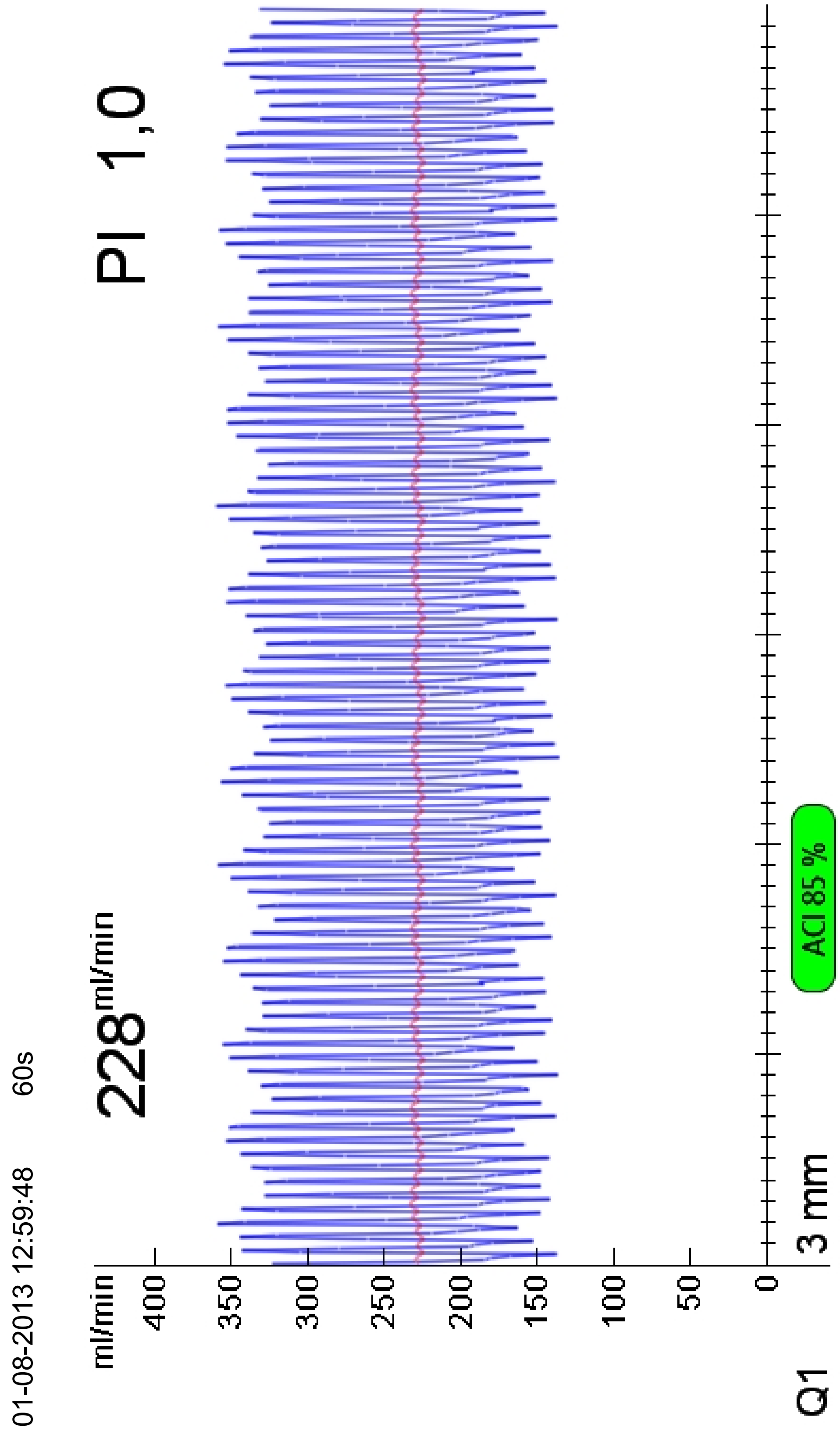

Skejby Sygehus

Urinvejskirurgisk afdeling K

Surgeon:

Operation Date: 31-07-2013 13:44:55

Patient Name: chris gris 10

Comments:

Patient ID:

Birthdate: Gender:

Height: Weight:

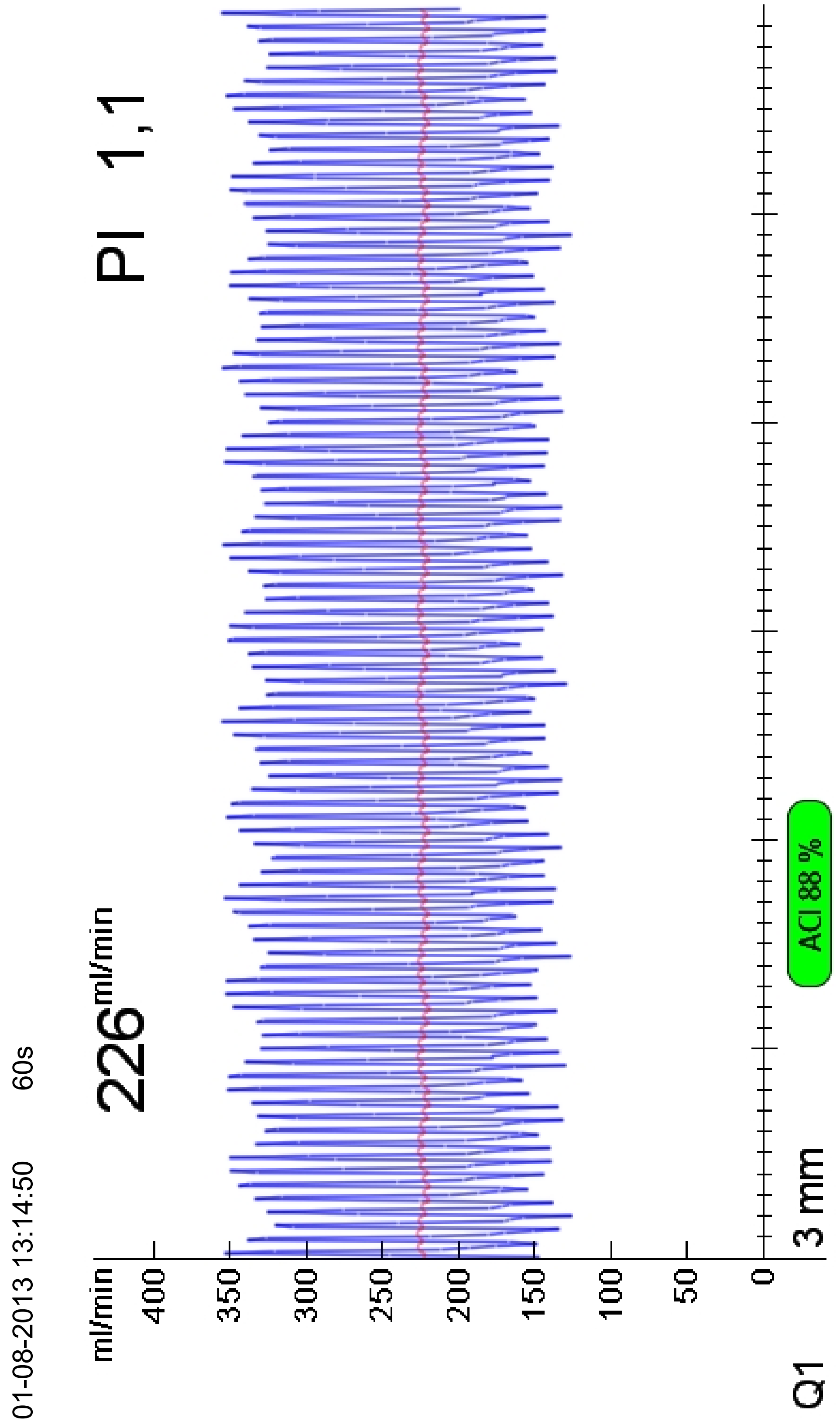

Skejby Sygehus

MEDISTIM

Urinvejskirurgisk afdeling K

Surgeon:

Operation Date: 31-07-2013 13:44:55

Patient Name: chris gris 10

Comments:

Patient ID:

Birthdate: Gender:

Height: Weight:

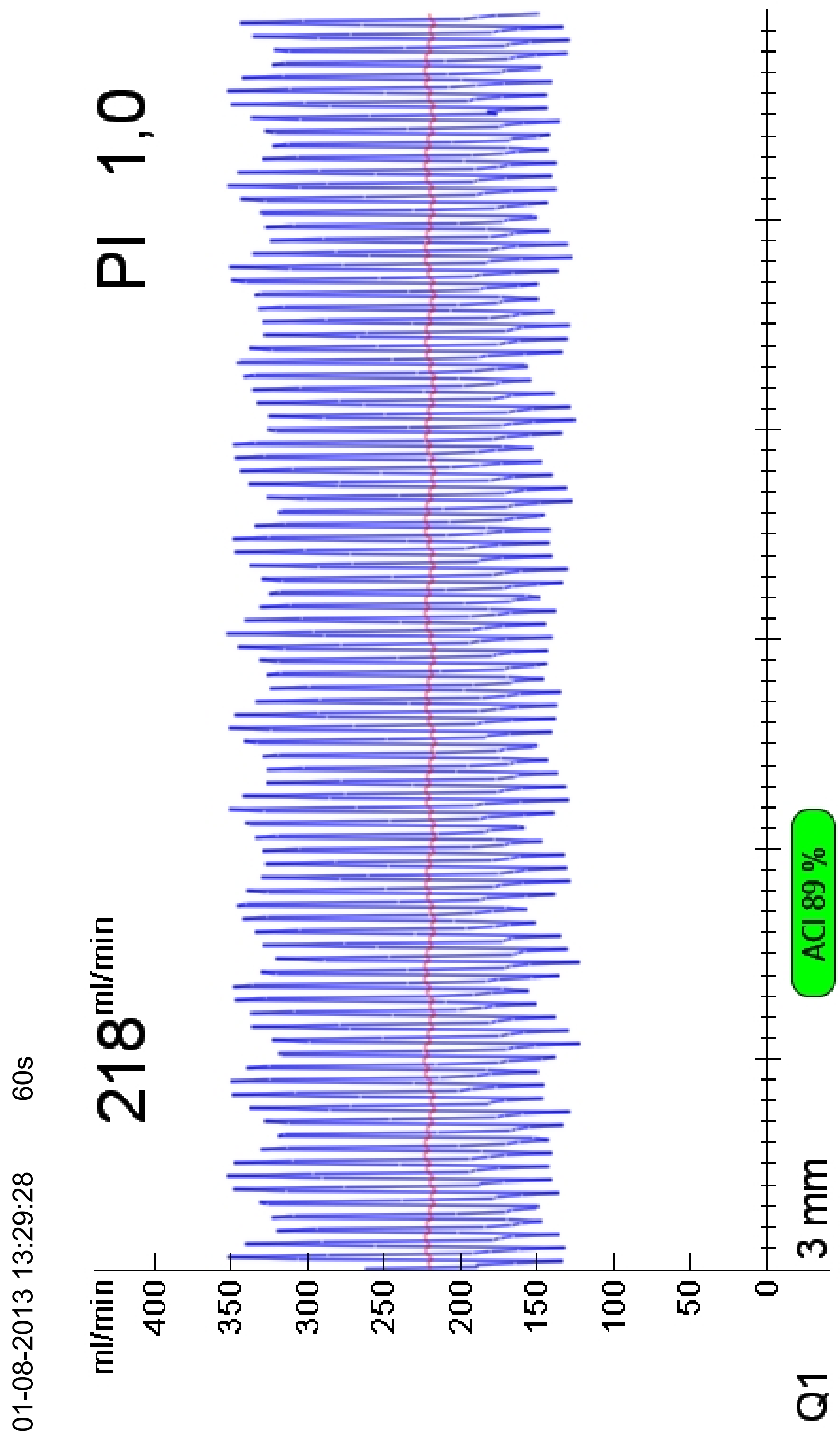

Skejby Sygehus

Urinvejskirurgisk afdeling K

MEDISTIM

Surgeon:

Operation Date: 31-07-2013 13:44:55

Patient Name: chris gris 10

Comments:

Patient ID:

Birthdate:

Gender:

Height:

Weight:

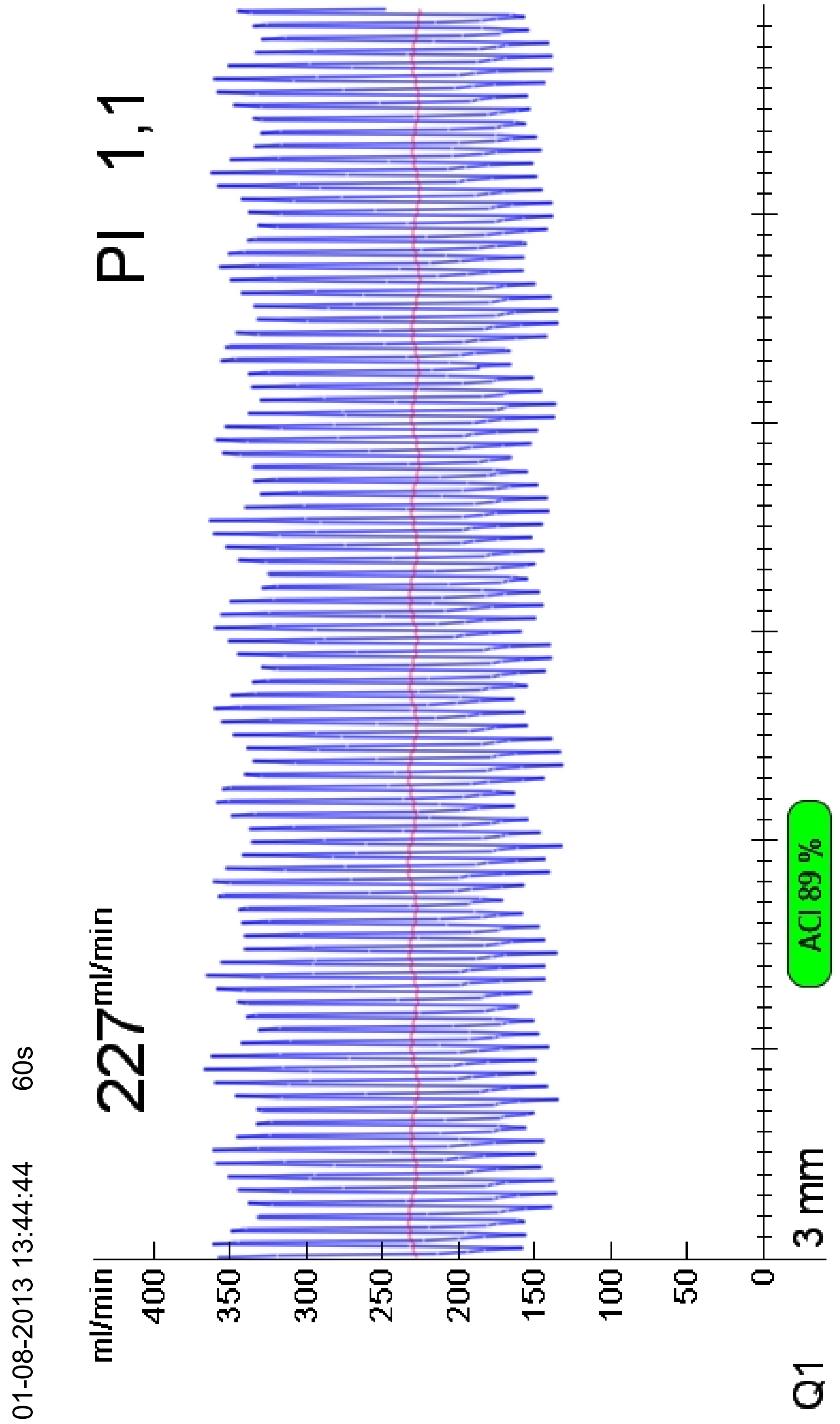

Skejby Sygehus

Urinvejskirurgisk afdeling K

Surgeon:

Operation Date: 31-07-2013 13:44:55

Patient Name: chris gris 10

Comments:

Patient ID:

Birthdate:

Gender:

Height:

Weight:

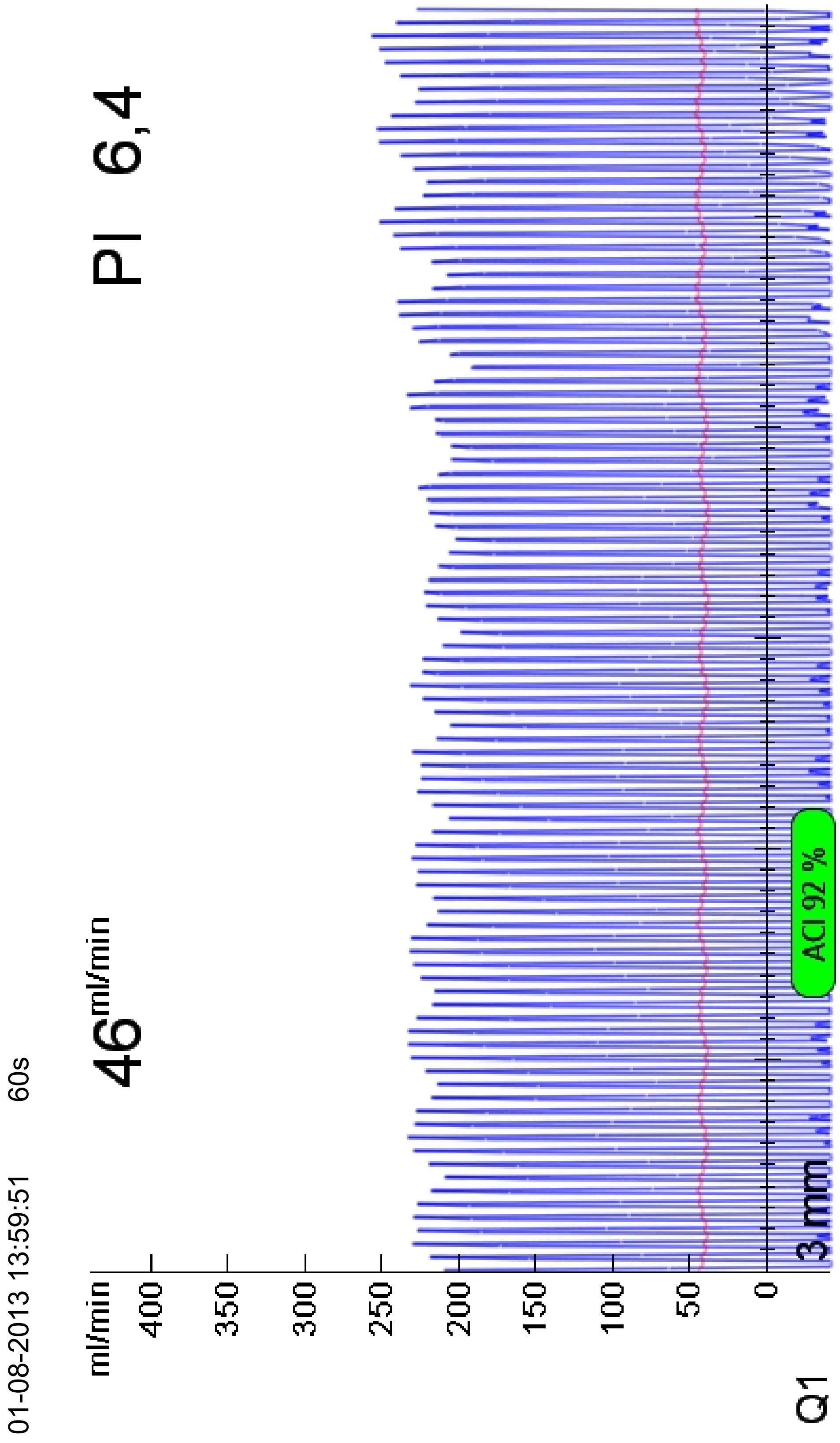

Skejby Sygehus

Urinvejskirurgisk afdeling K

MEDISTIM

Surgeon:

Operation Date: 31-07-2013 13:44:55

Patient Name: chris gris 10

Comments:

Patient ID:

Birthdate:

Gender:

Height:

Weight:

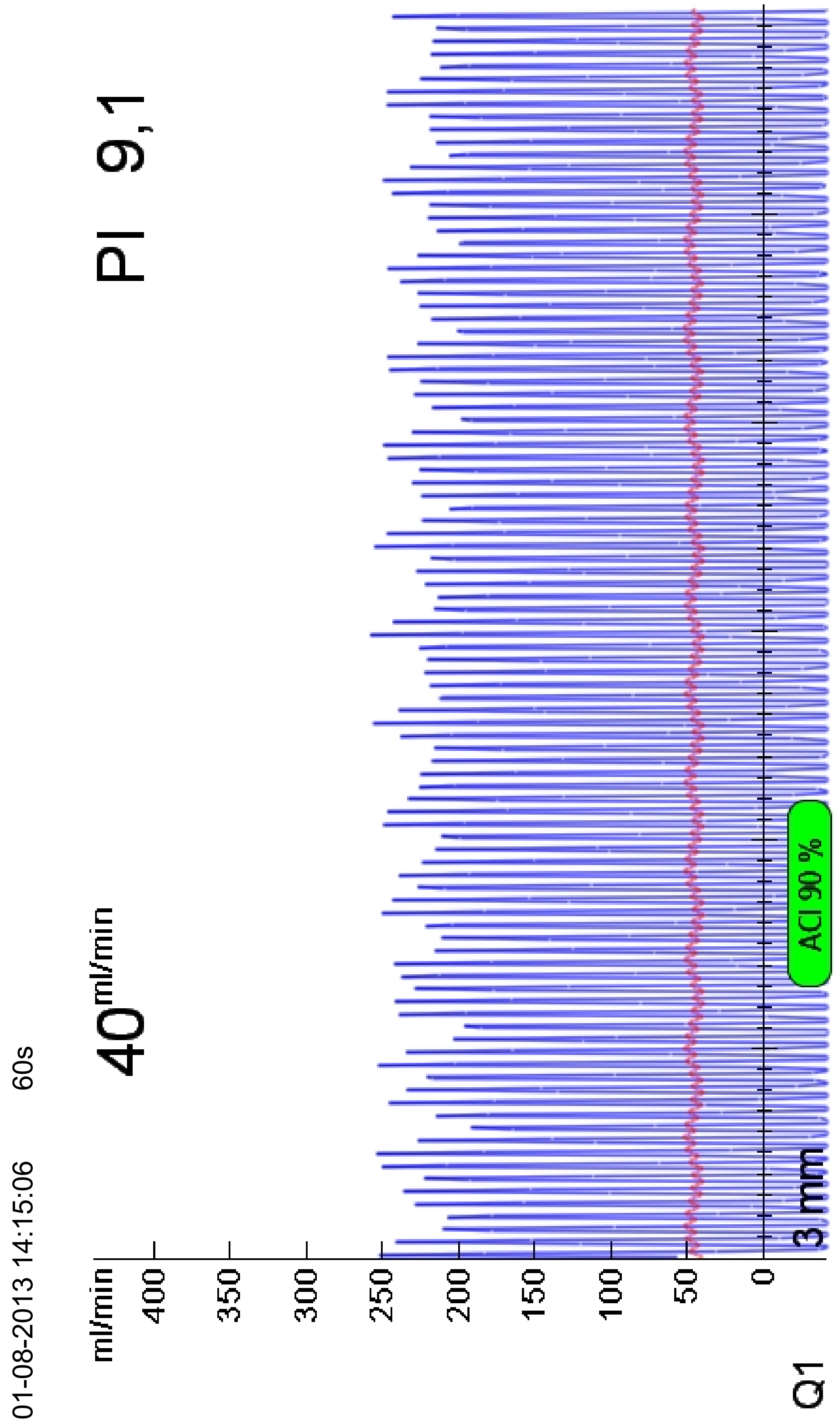

Skejby Sygehus

Urinvejskirurgisk afdeling K

MEDISTIM

Surgeon:

Operation Date: 31-07-2013 13:44:55

Patient Name: chris gris 10

Comments:

Patient ID:

Birthdate:

Gender:

Height:

Weight:

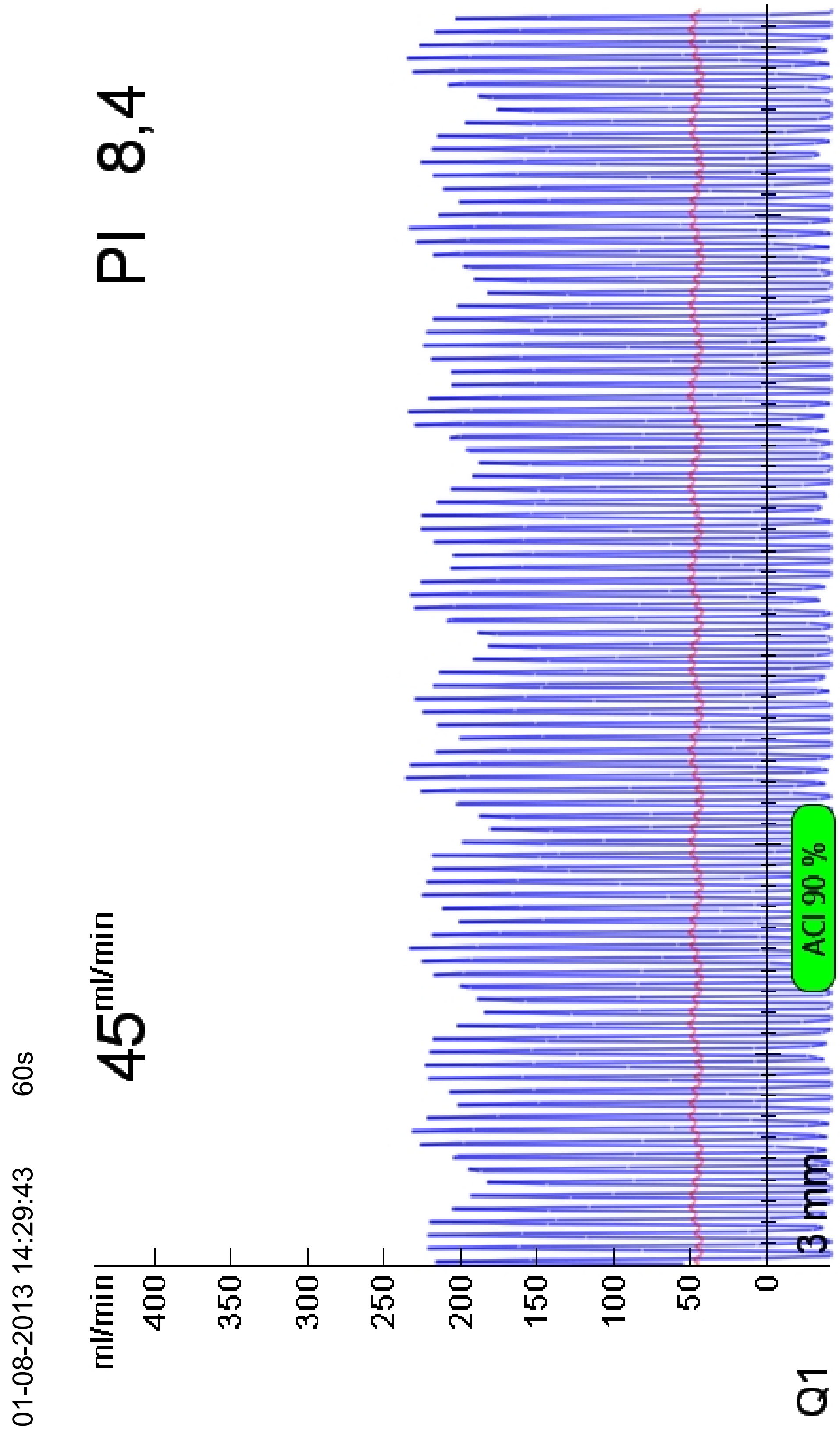

Skejby Sygehus

Urinvejskirurgisk afdeling K

Surgeon:

Operation Date: 31-07-2013 13:44:55

Patient Name: chris gris 10

Patient ID:

Birthdate:

Gender:

Height:

Weight:

Comments:

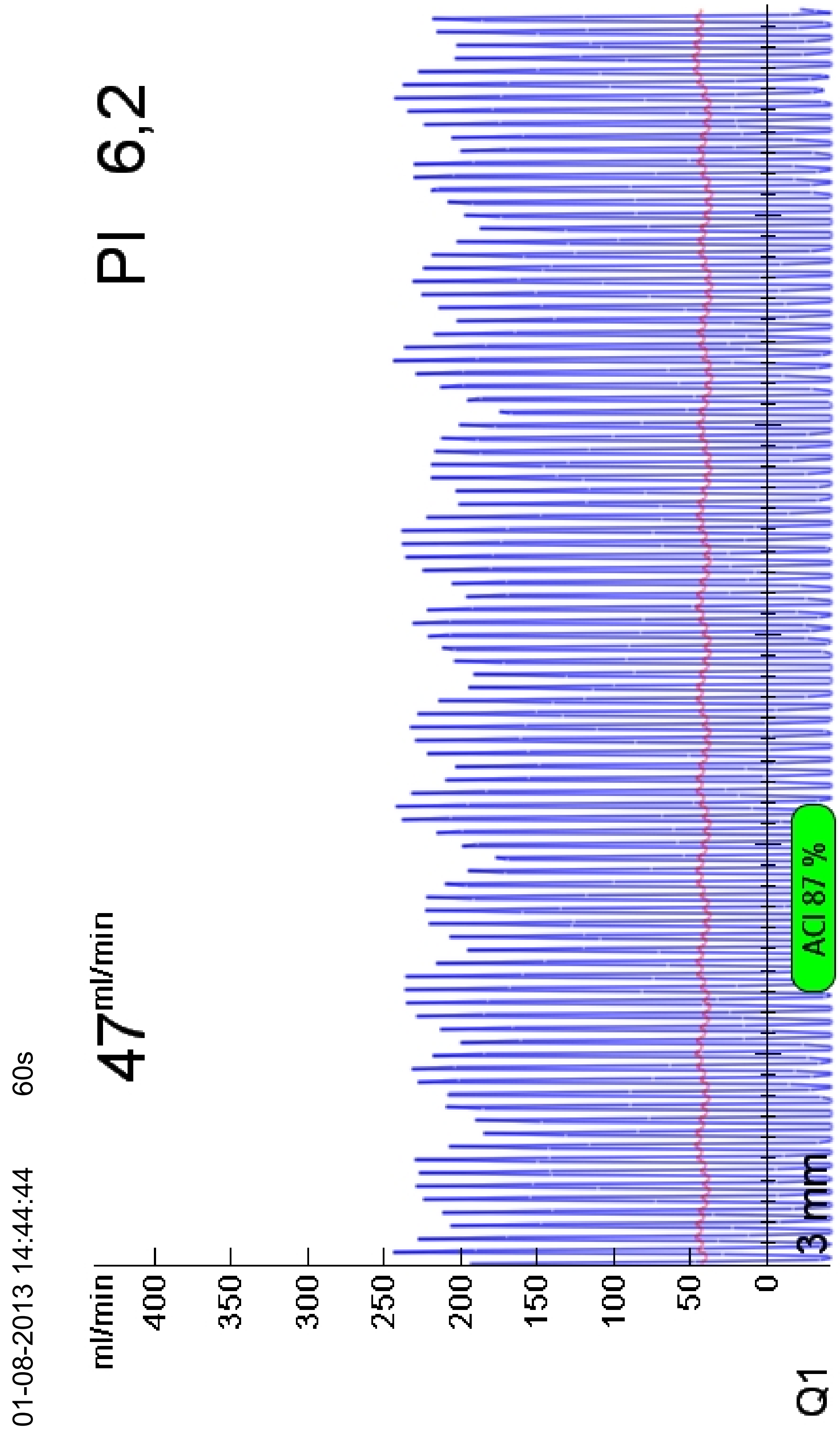

Skejby Sygehus

Urinvejskirurgisk afdeling K

MEDISTIM

Surgeon:

Operation Date: 31-07-2013 13:44:55

Patient Name: chris gris 10

Comments:

Patient ID:

Birthdate: Gender:

Height: Weight:

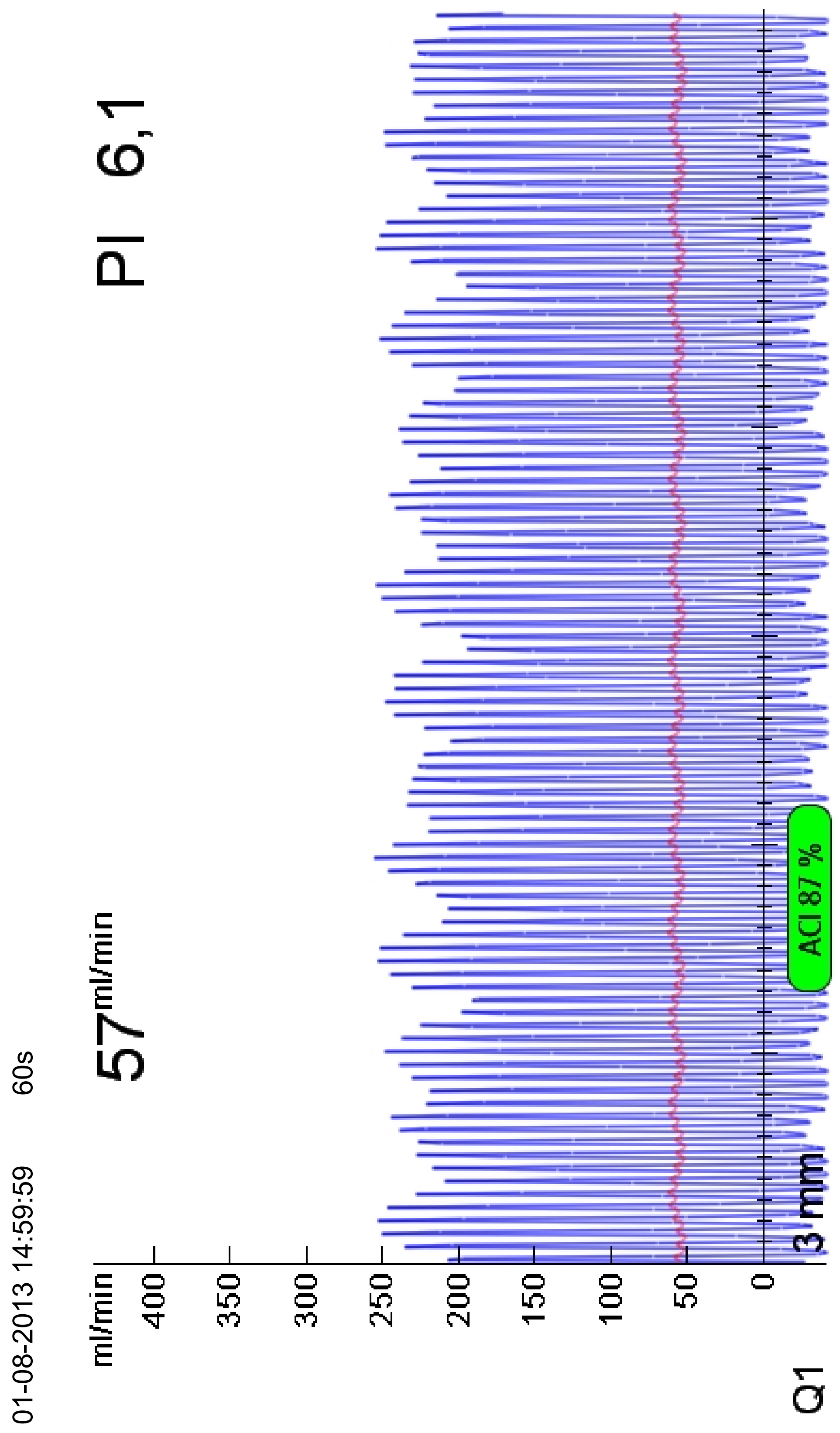

Skejby Sygehus

Urinvejskirurgisk afdeling K

Surgeon:

Operation Date: 31-07-2013 13:44:55

Patient Name: chris gris 10

Comments:

Patient ID:

Birthdate: Gender:

Height: Weight:

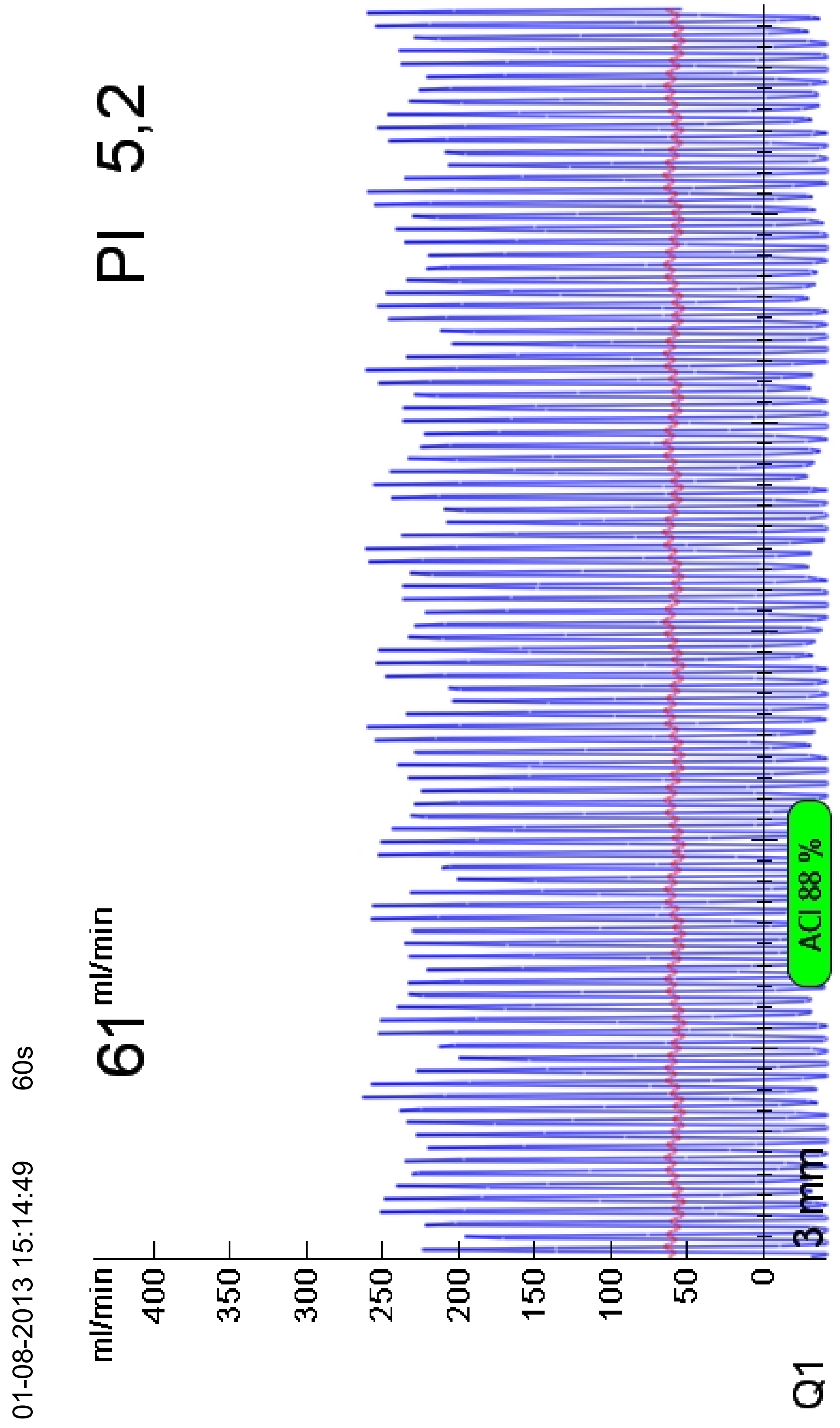

Skejby Sygehus

Urinvejskirurgisk afdeling K

Surgeon:

Operation Date: 31-07-2013 13:44:55

Patient Name: chris gris 10

Patient ID:

Birthdate:

Gender:

Height:

Weight:

Comments:

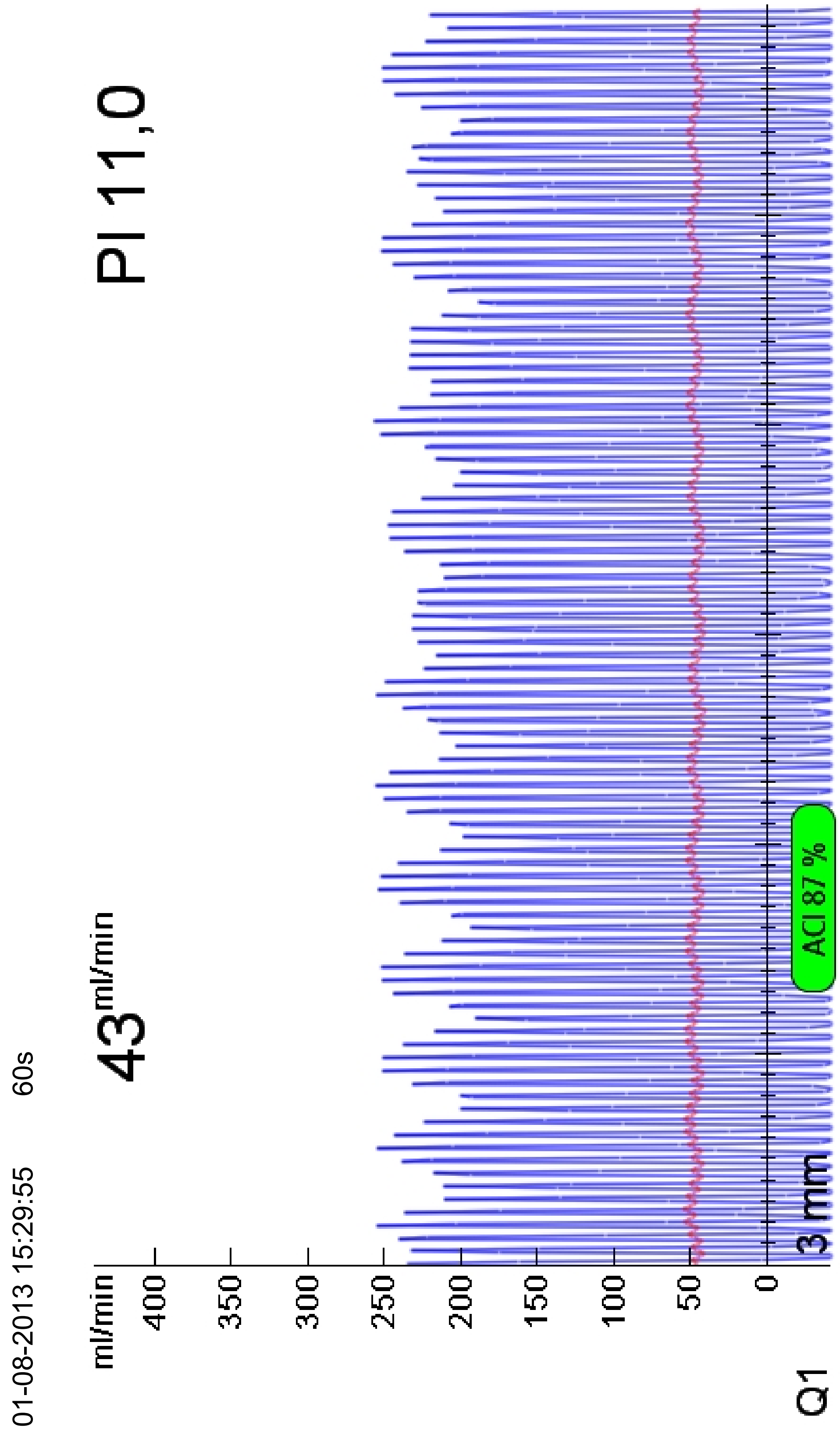

Skejby Sygehus

Urinvejskirurgisk afdeling K

Surgeon:

Operation Date: 31-07-2013 13:44:55

Patient Name: chris gris 10

Comments:

Patient ID:

Birthdate: Gender:

Height: Weight:

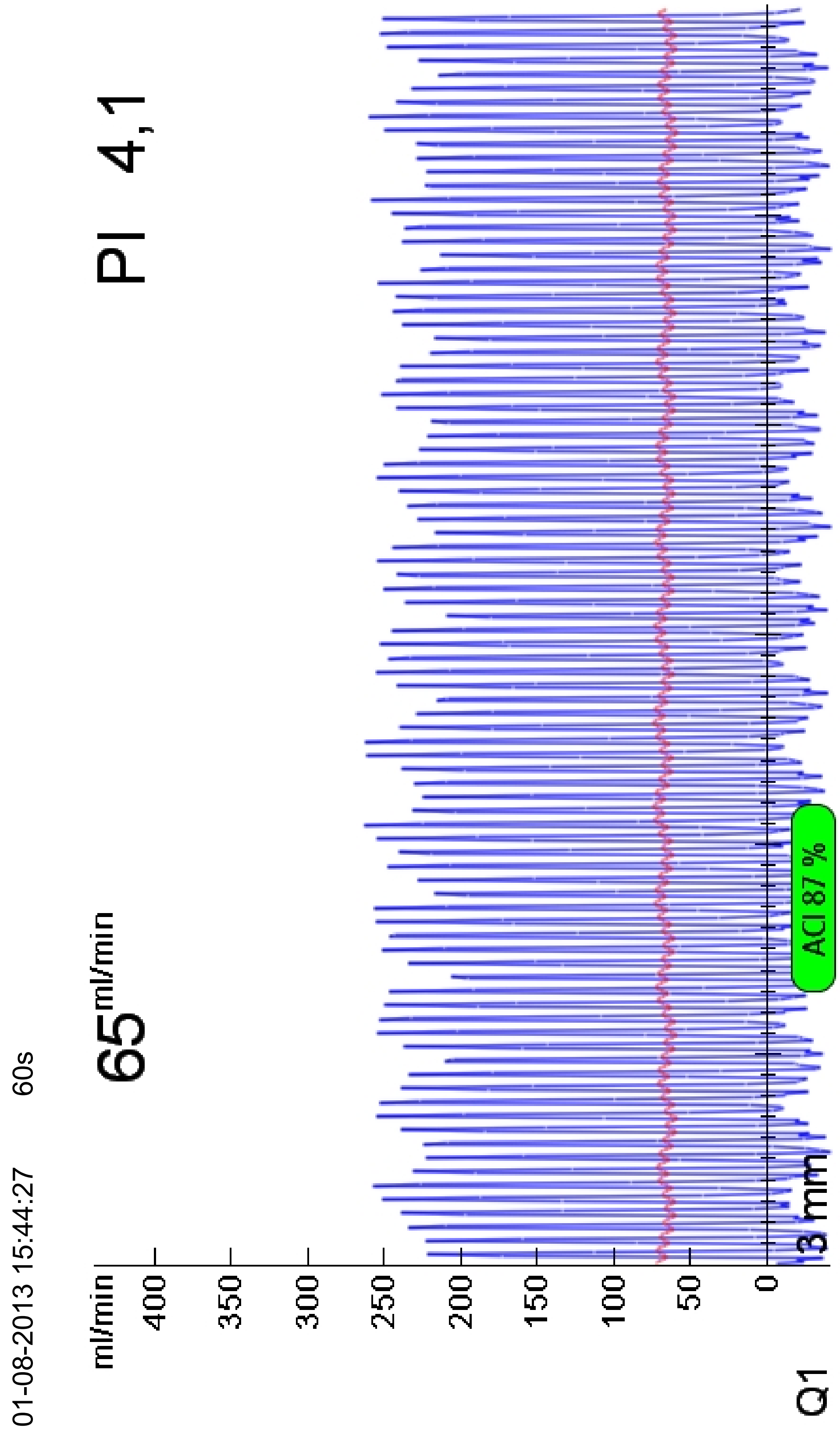

Skejby Sygehus

MEDISTIM

Urinvejskirurgisk afdeling K

Surgeon:

Operation Date: 31-07-2013 13:44:55

Patient Name: chris gris 10

Comments:

Patient ID:

Birthdate: Gender:

Height: Weight:

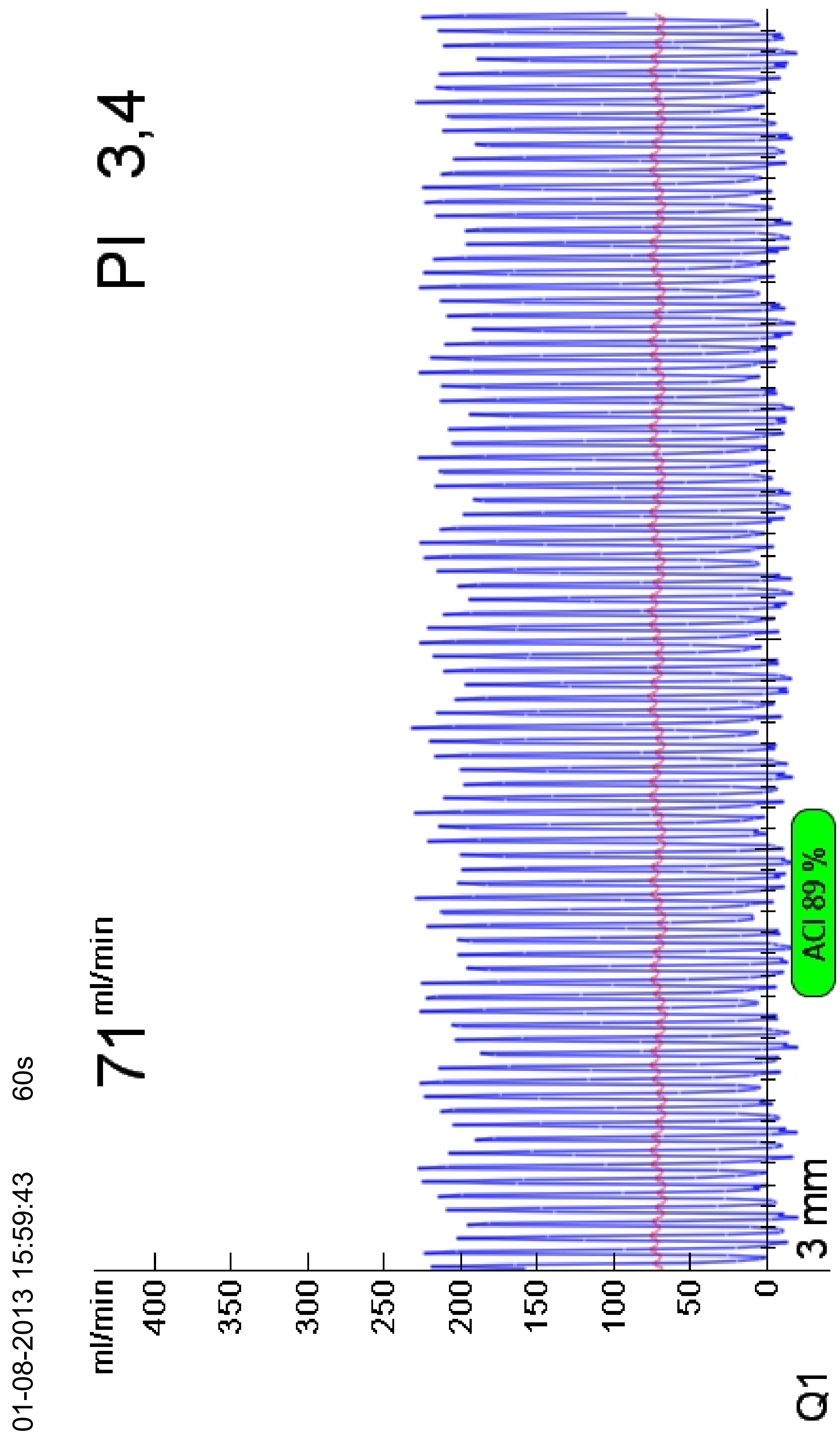

Skejby Sygehus

MEDISTIM

Urinvejskirurgisk afdeling K

Surgeon:

Operation Date: 31-07-2013 13:44:55

Patient Name: chris gris 10

Comments:

Patient ID:

Birthdate: Gender:

Height: Weight:

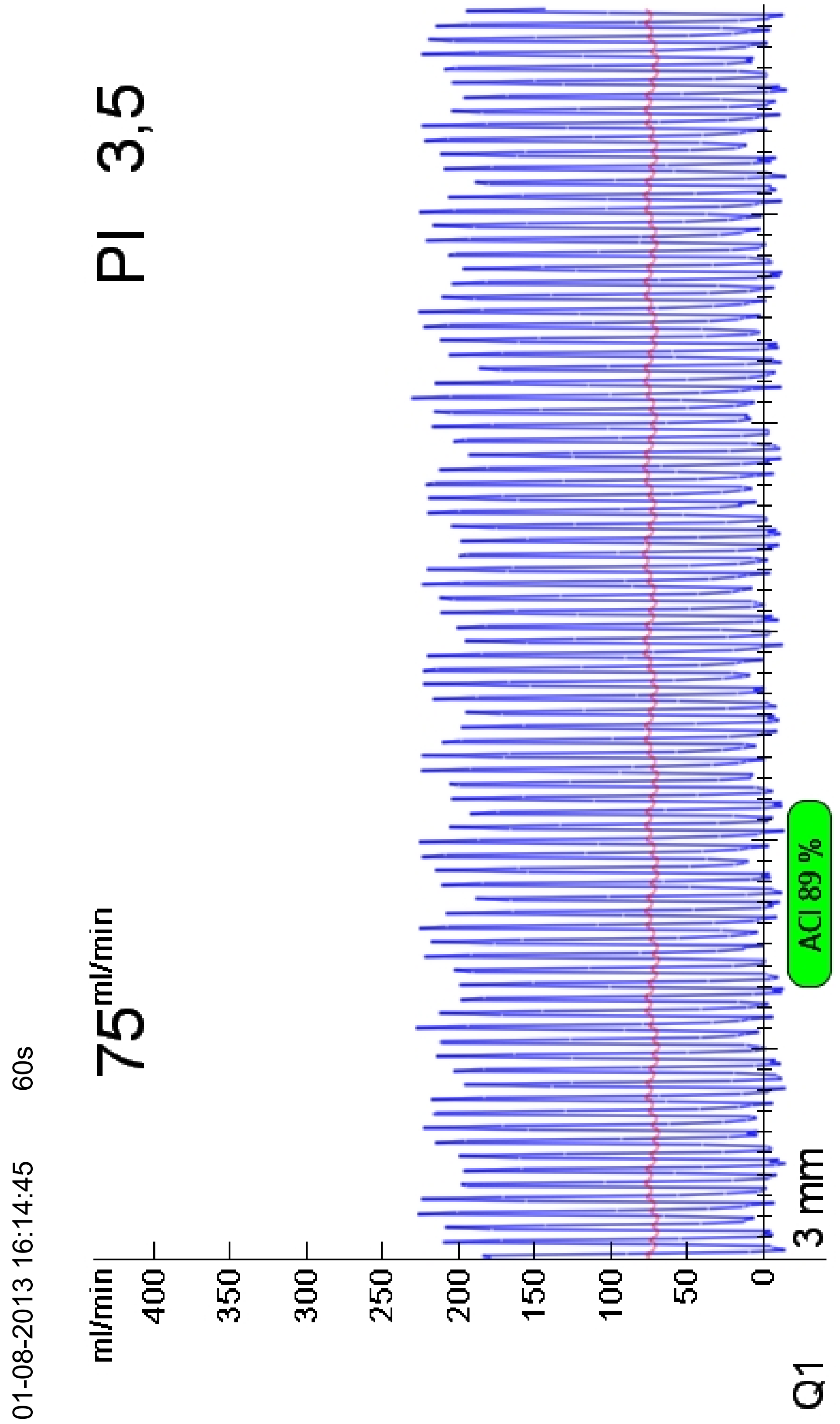

Skejby Sygehus

Urinvejskirurgisk afdeling K

Surgeon:

Operation Date: 31-07-2013 13:44:55

Patient Name: chris gris 10

Patient ID:

Birthdate: Gender:

Height: Weight:

Comments:

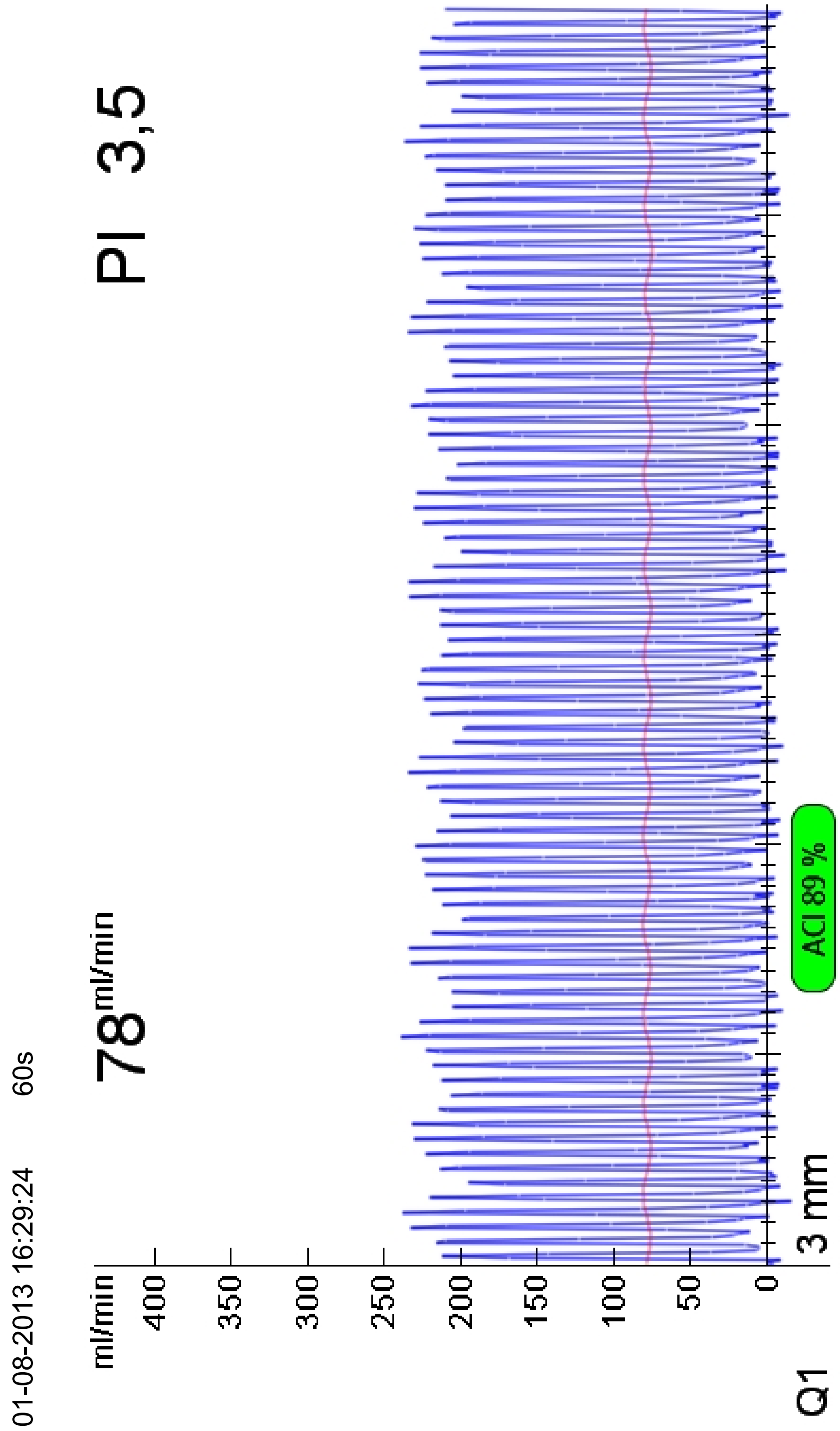

Skejby Sygehus

Urinvejskirurgisk afdeling K

Surgeon:

Operation Date: 31-07-2013 13:44:55

Patient Name: chris gris 10

Comments:

Patient ID:

Birthdate: Gender:

Height: Weight:

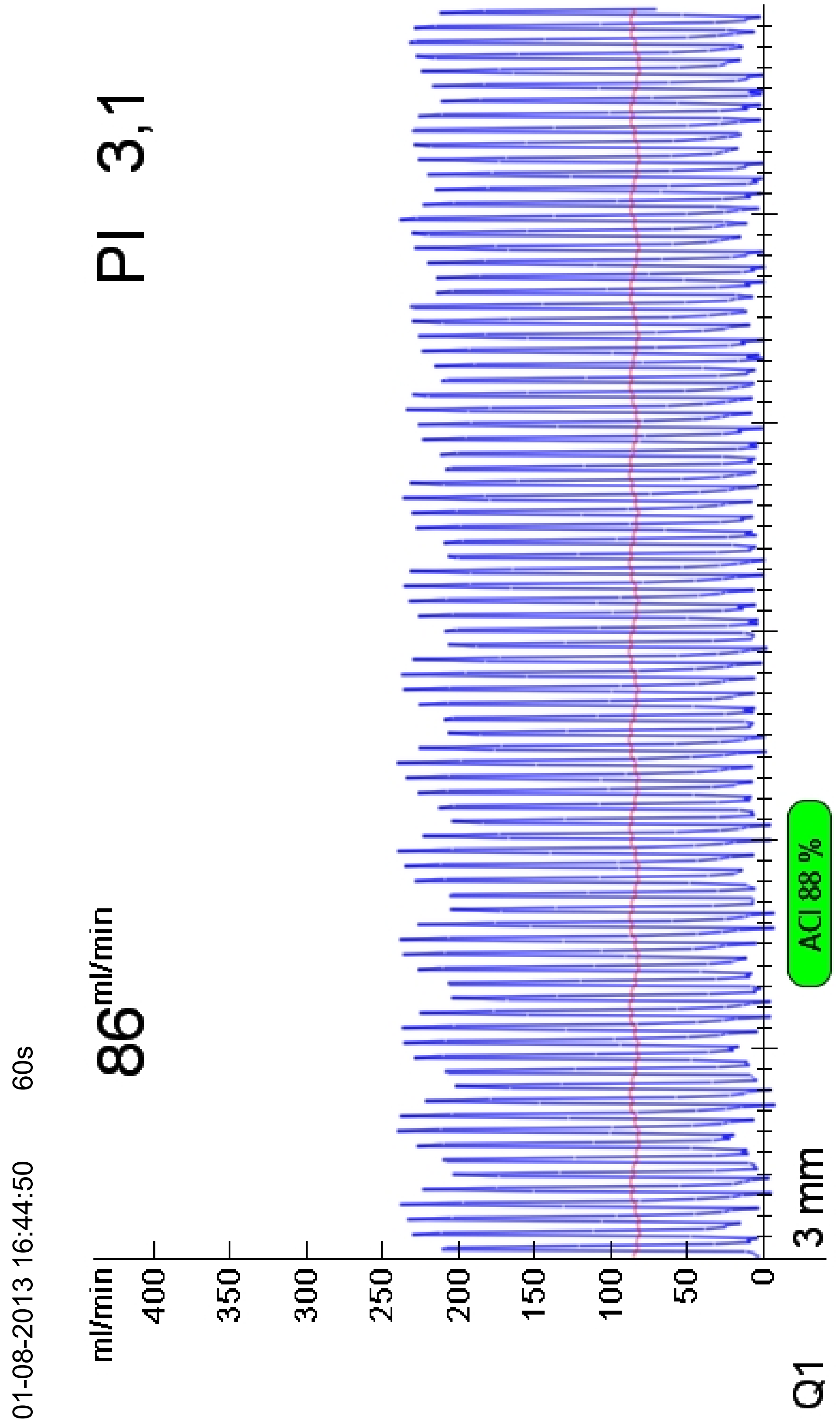

Skejby Sygehus

MEDISTIM

Urinvejskirurgisk afdeling K

Surgeon:

Operation Date: 31-07-2013 13:44:55

Patient Name: chris gris 10

Comments:

Patient ID:

Birthdate: Gender:

Height: Weight:

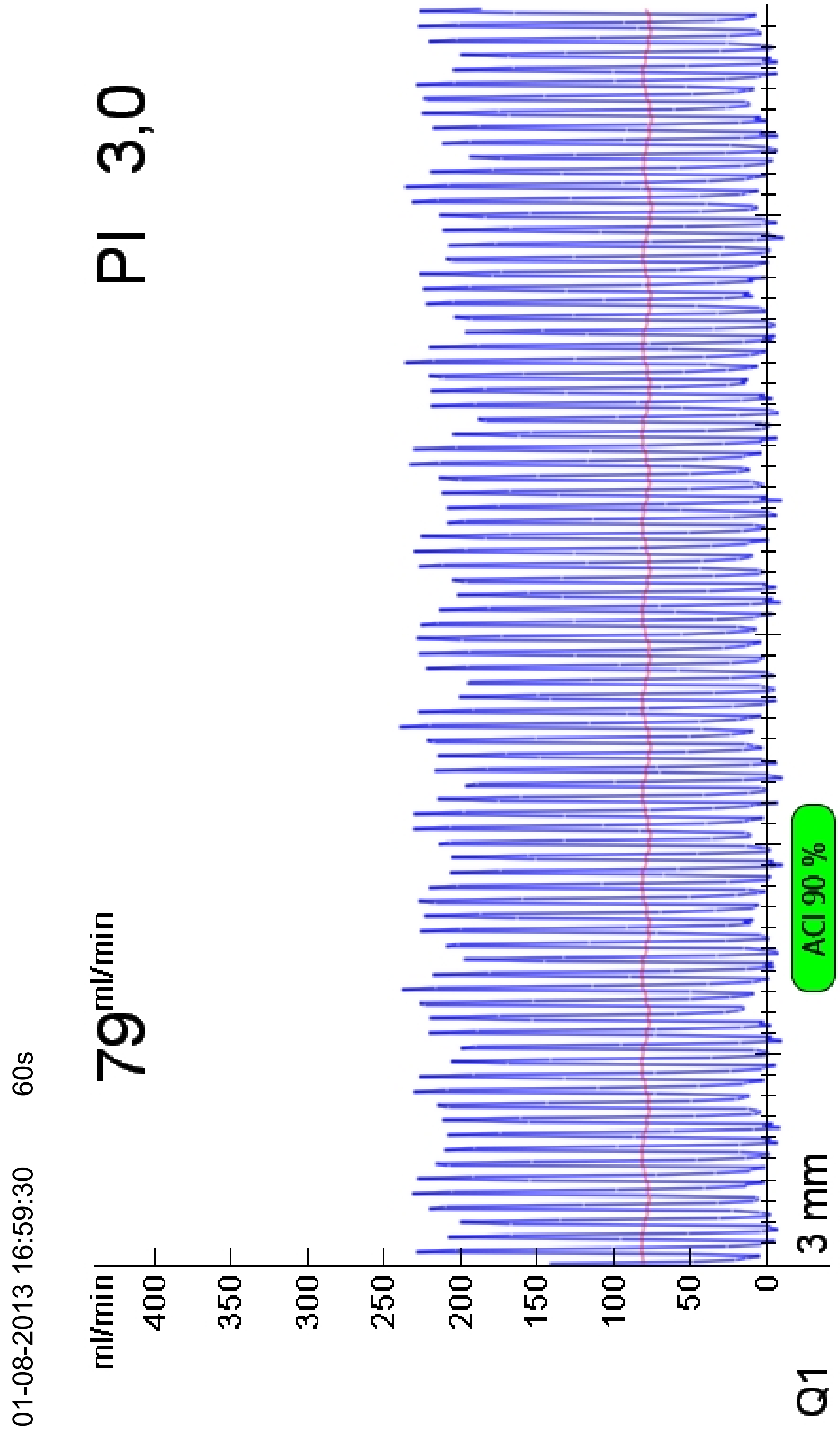

Skejby Sygehus

MEDISTIM

Urinvejskirurgisk afdeling K

Surgeon:

Operation Date: 31-07-2013 13:44:55

Patient Name: chris gris 10

Comments:

Patient ID:

Birthdate:

Gender:

Height:

Weight:

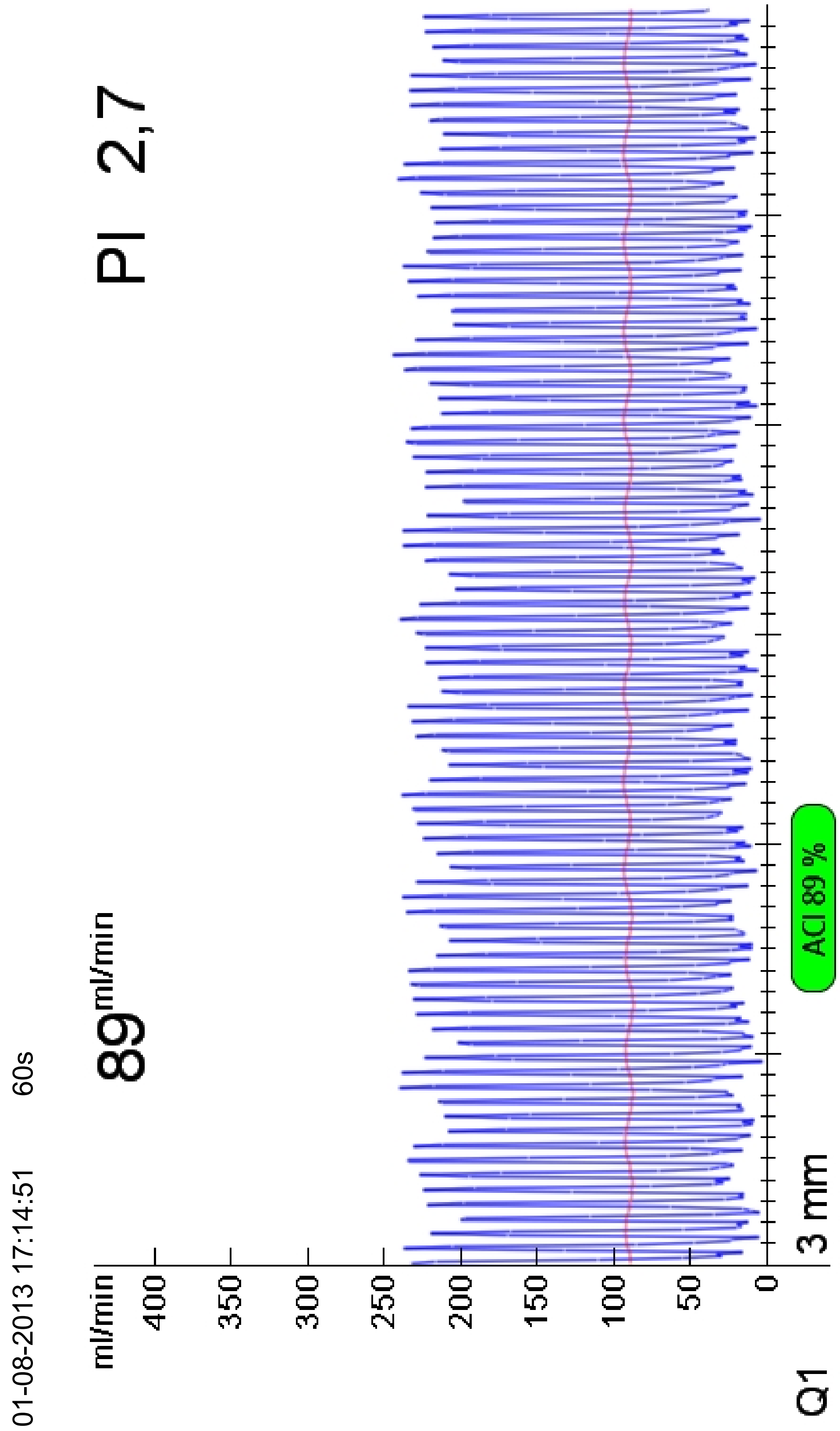

Skejby Sygehus

Urinvejskirurgisk afdeling K

Surgeon: Operation Date: 31-07-2013 13:44:55

Patient Name: chris gris 10

Comments:

Patient ID:

Birthdate: Gender:

Height: Weight:

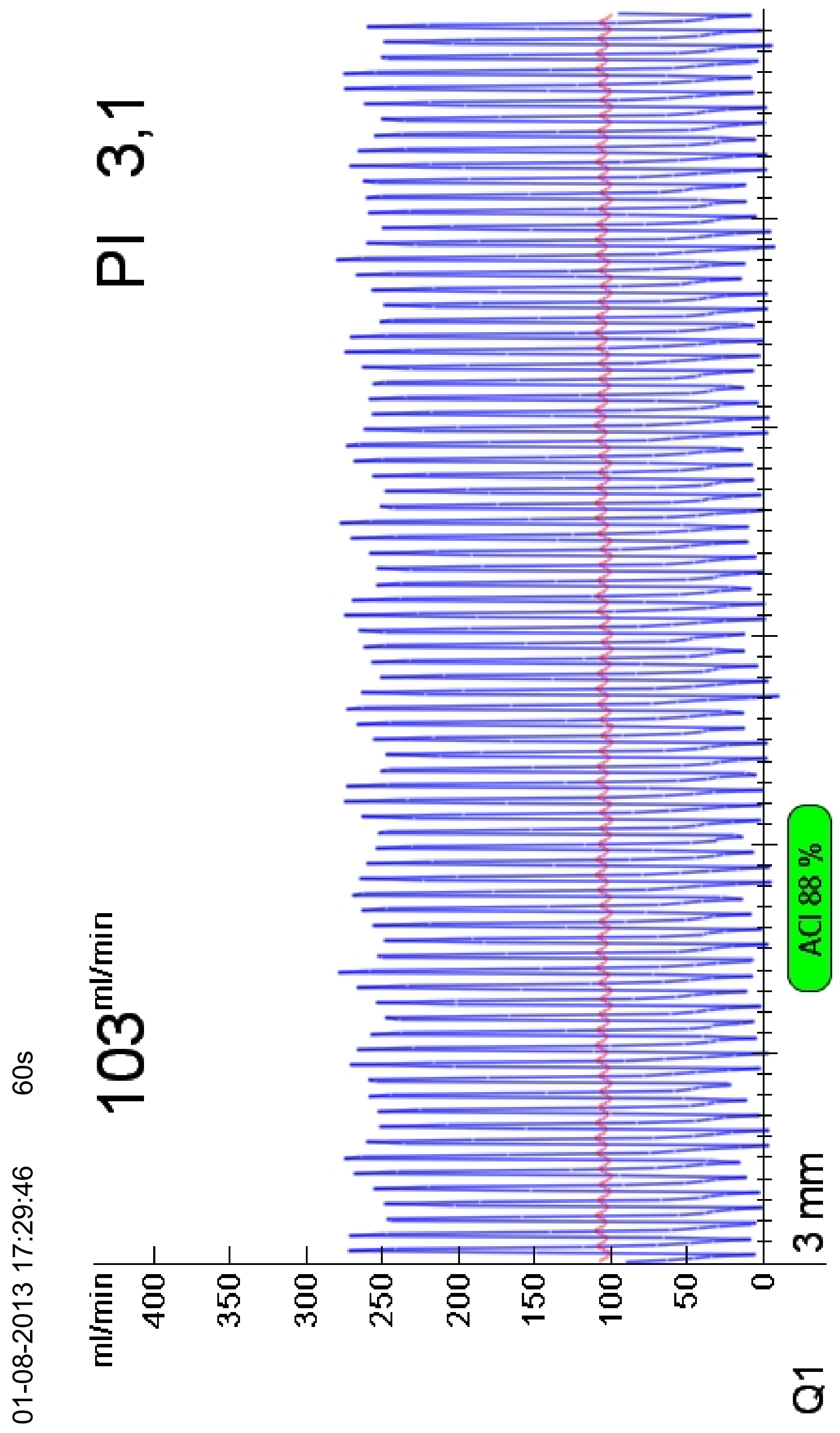

Skejby Sygehus

Urinvejskirurgisk afdeling K

Surgeon:

Operation Date: 31-07-2013 13:44:55

Patient Name: chris gris 10

Comments:

Patient ID:

Birthdate: Gender:

Height: Weight:

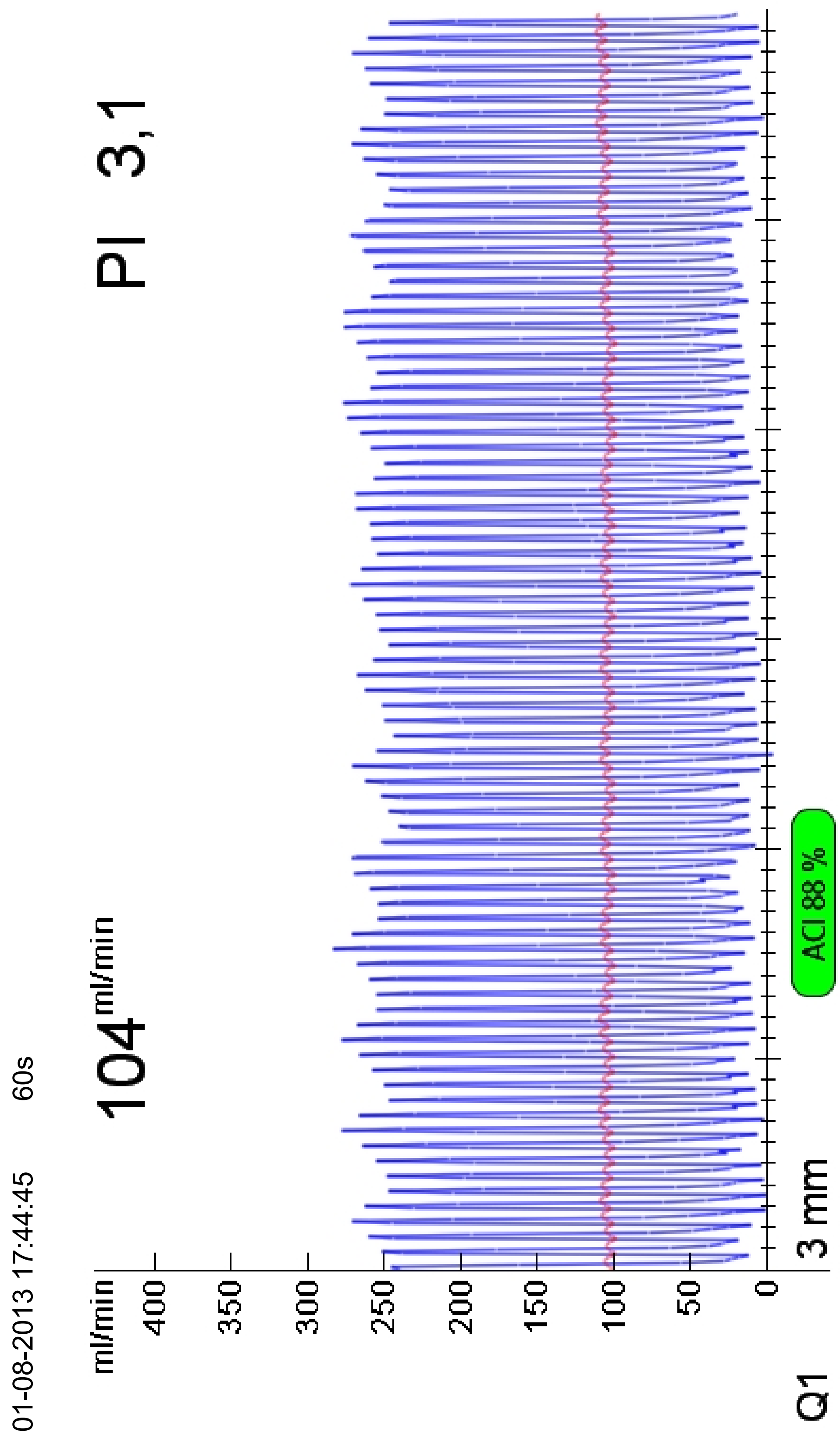

Skejby Sygehus

Urinvejskirurgisk afdeling K

MEDISTIM

Surgeon:

Operation Date: 31-07-2013 13:44:55

Patient Name: chris gris 10

Comments:

Patient ID:

Birthdate: Gender:

Height: Weight:

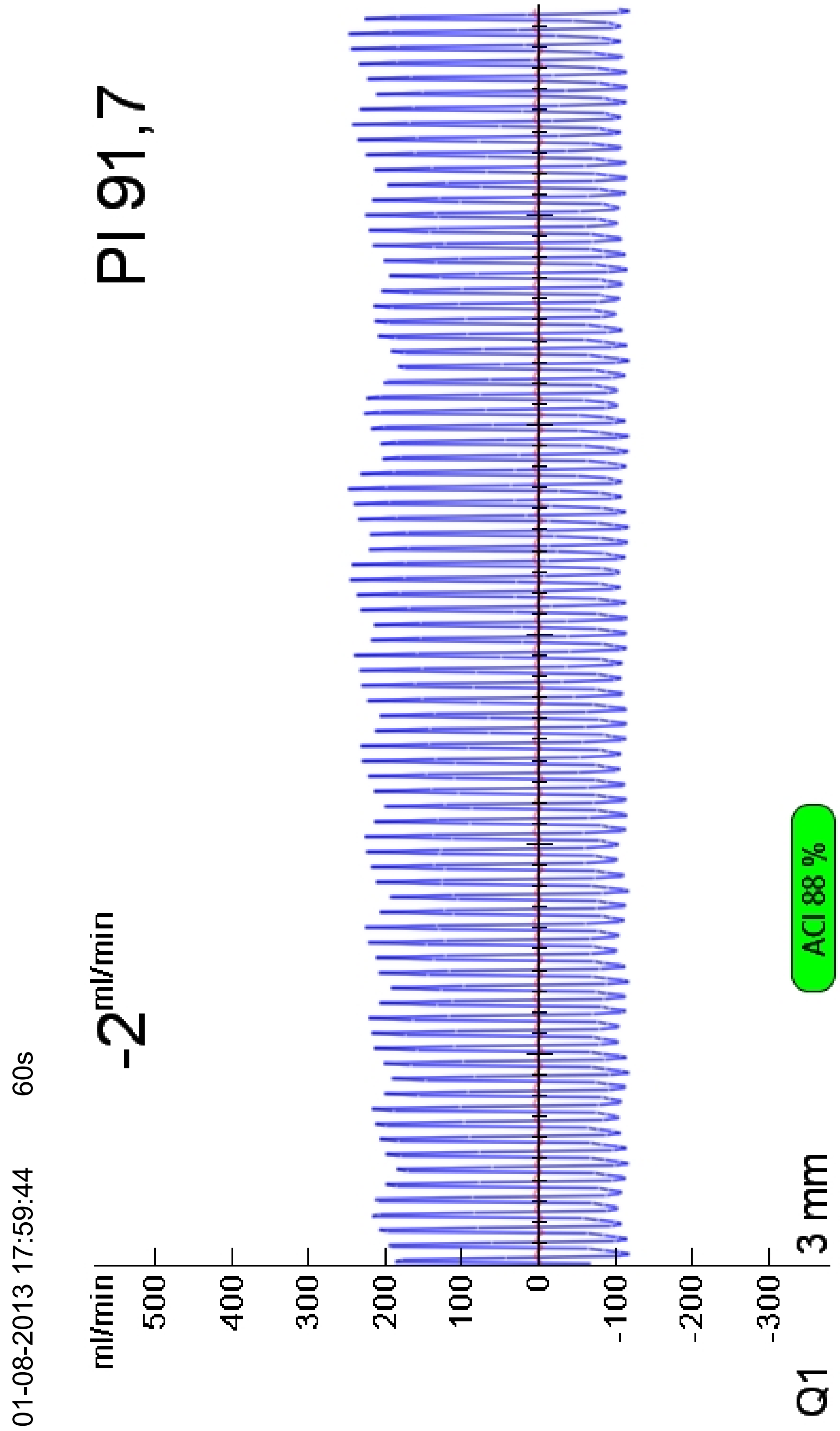

Skejby Sygehus

Urinvejskirurgisk afdeling K

Surgeon:

Operation Date: 31-07-2013 13:44:55

Patient Name: chris gris 10

Comments:

Patient ID:

Birthdate:

Gender:

Height:

Weight:

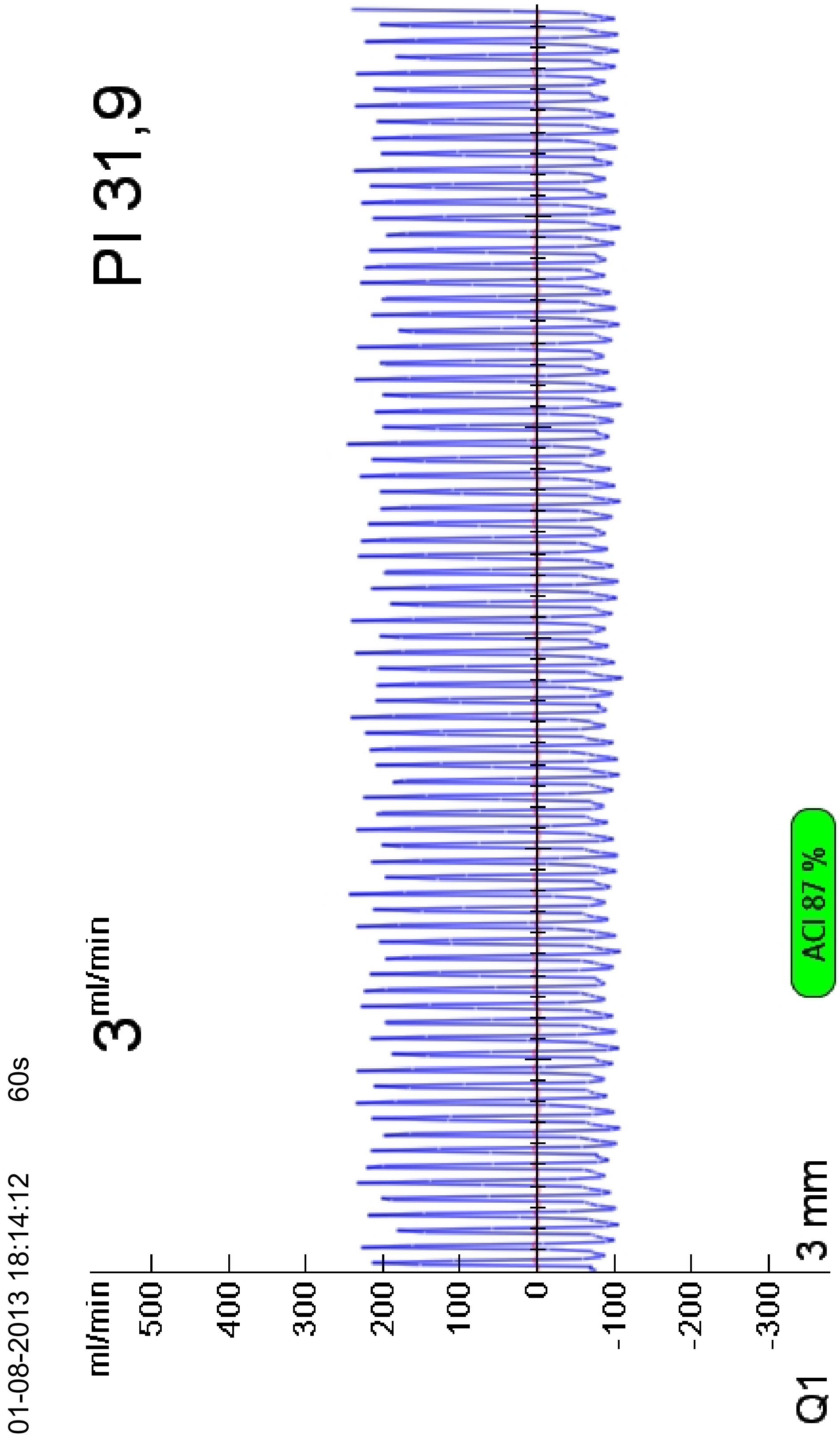

Skejby Sygehus

Urinvejskirurgisk afdeling K

MEDISTIM

Surgeon:

Operation Date: 31-07-2013 13:44:55

Patient Name: chris gris 10

Comments:

Patient ID:

Birthdate: Gender:

Height: Weight:

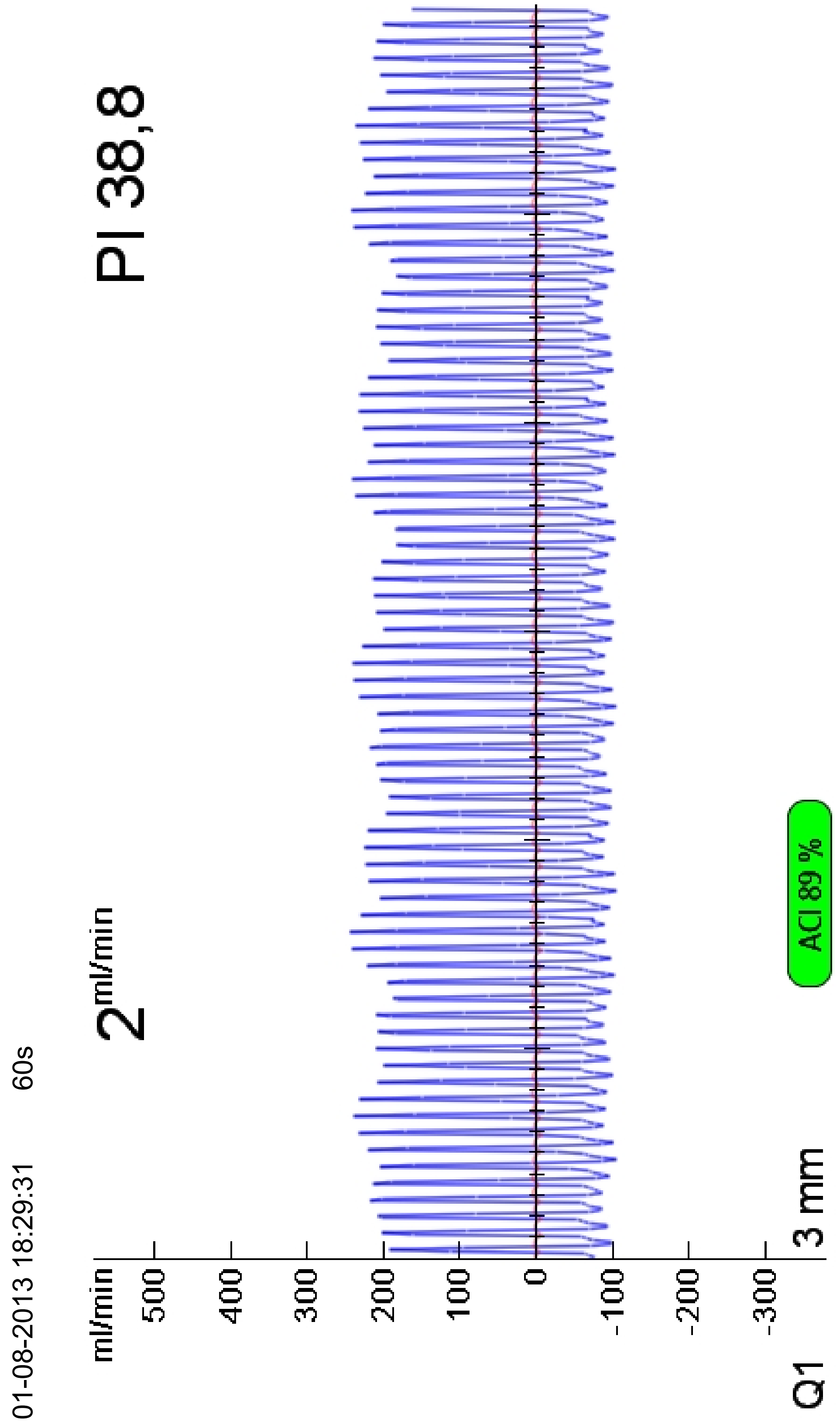

Skejby Sygehus

Urinvejskirurgisk afdeling K

Surgeon:

Operation Date: 31-07-2013 13:44:55

Patient Name: chris gris 10

Comments:

Patient ID:

Birthdate: Gender:

Height: Weight:

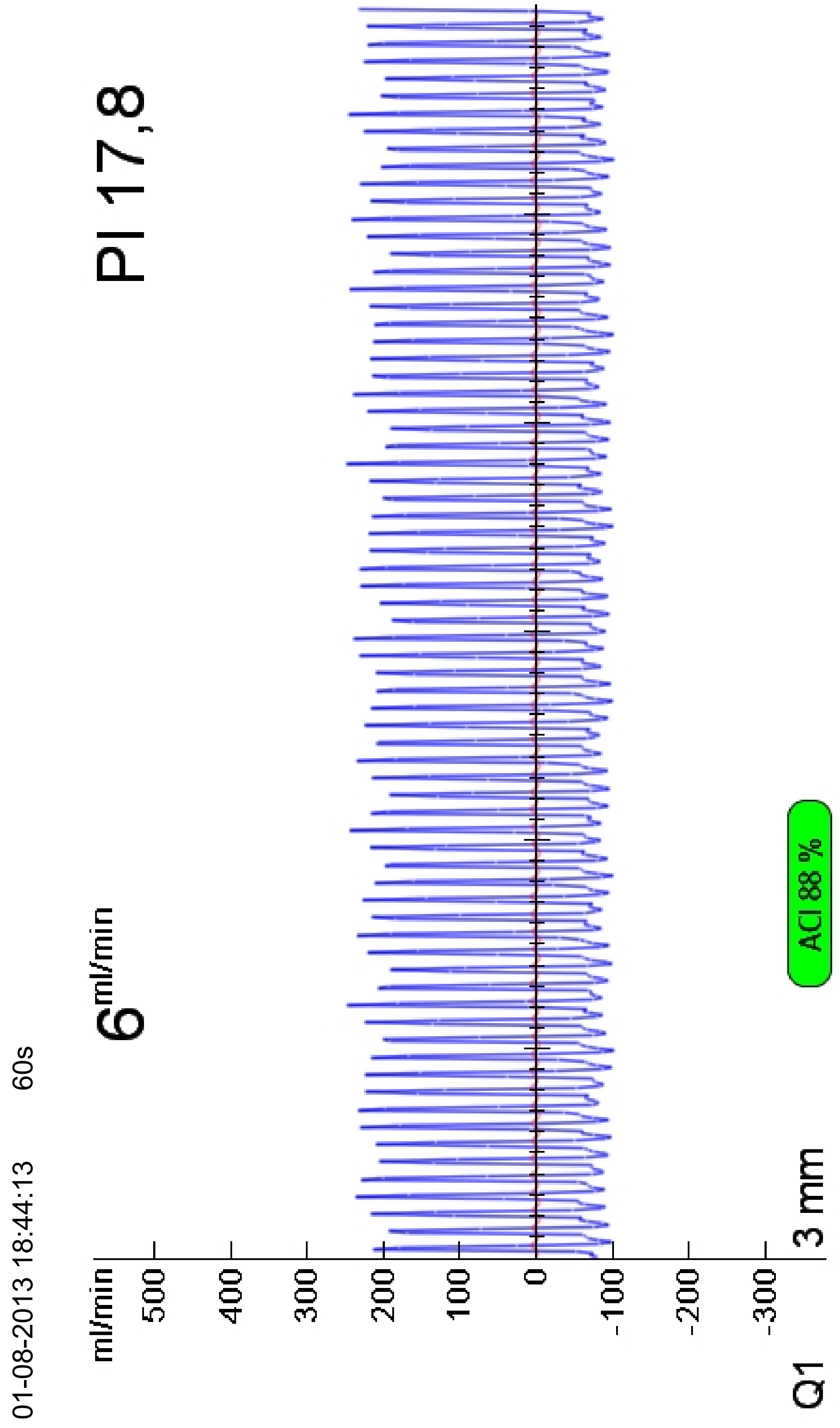

Skejby Sygehus

Urinvejskirurgisk afdeling K

MEDISTIM

Surgeon:

Operation Date: 31-07-2013 13:44:55

Patient Name: chris gris 10

Comments:

Patient ID:

Birthdate: Gender:

Height: Weight:

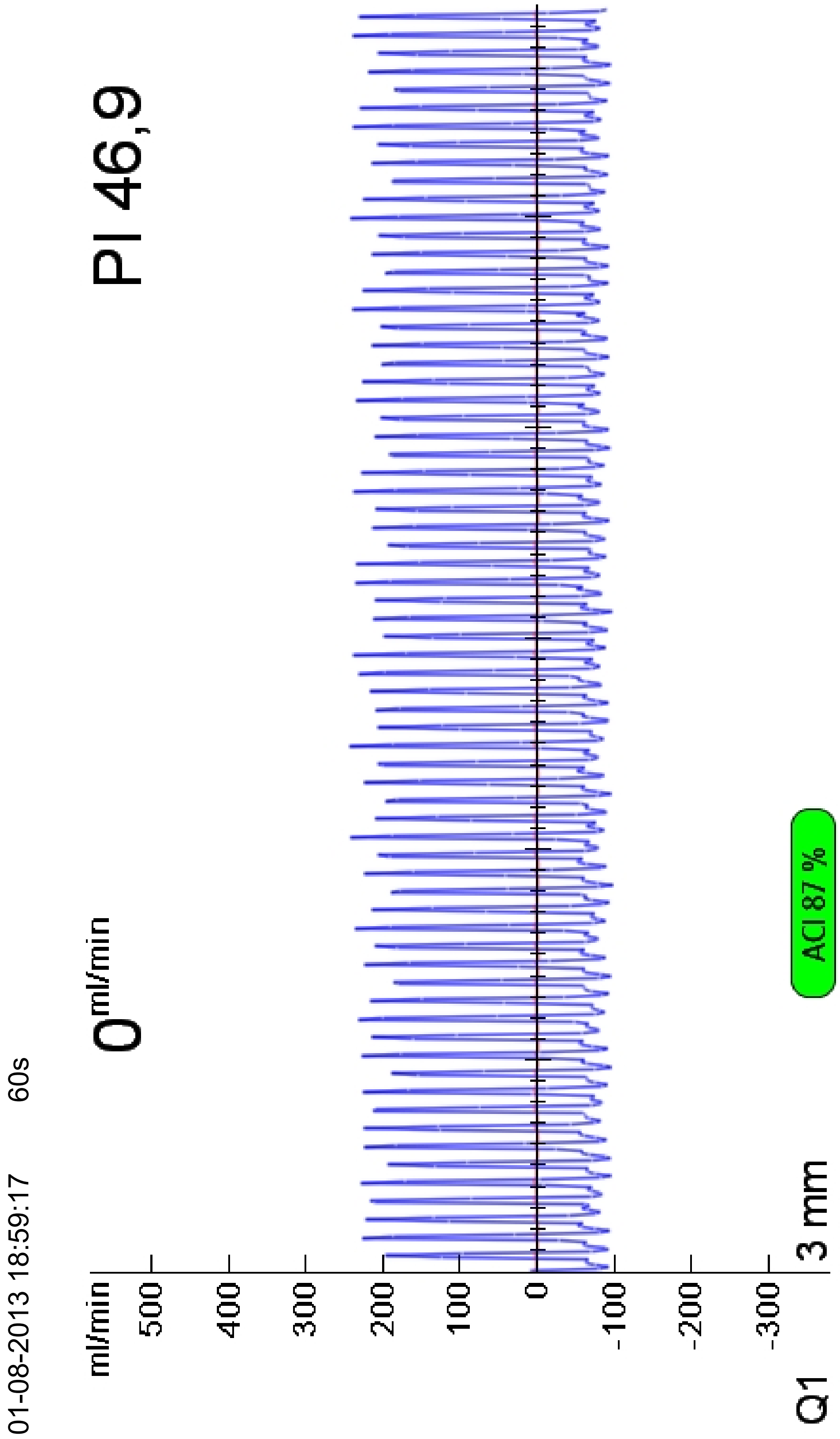

Skejby Sygehus

Urinvejskirurgisk afdeling K

MEDISTIM

Surgeon:

Operation Date: 31-07-2013 13:44:55

Patient Name: chris gris 10

Comments:

Patient ID:

Birthdate:

Gender:

Height:

Weight:

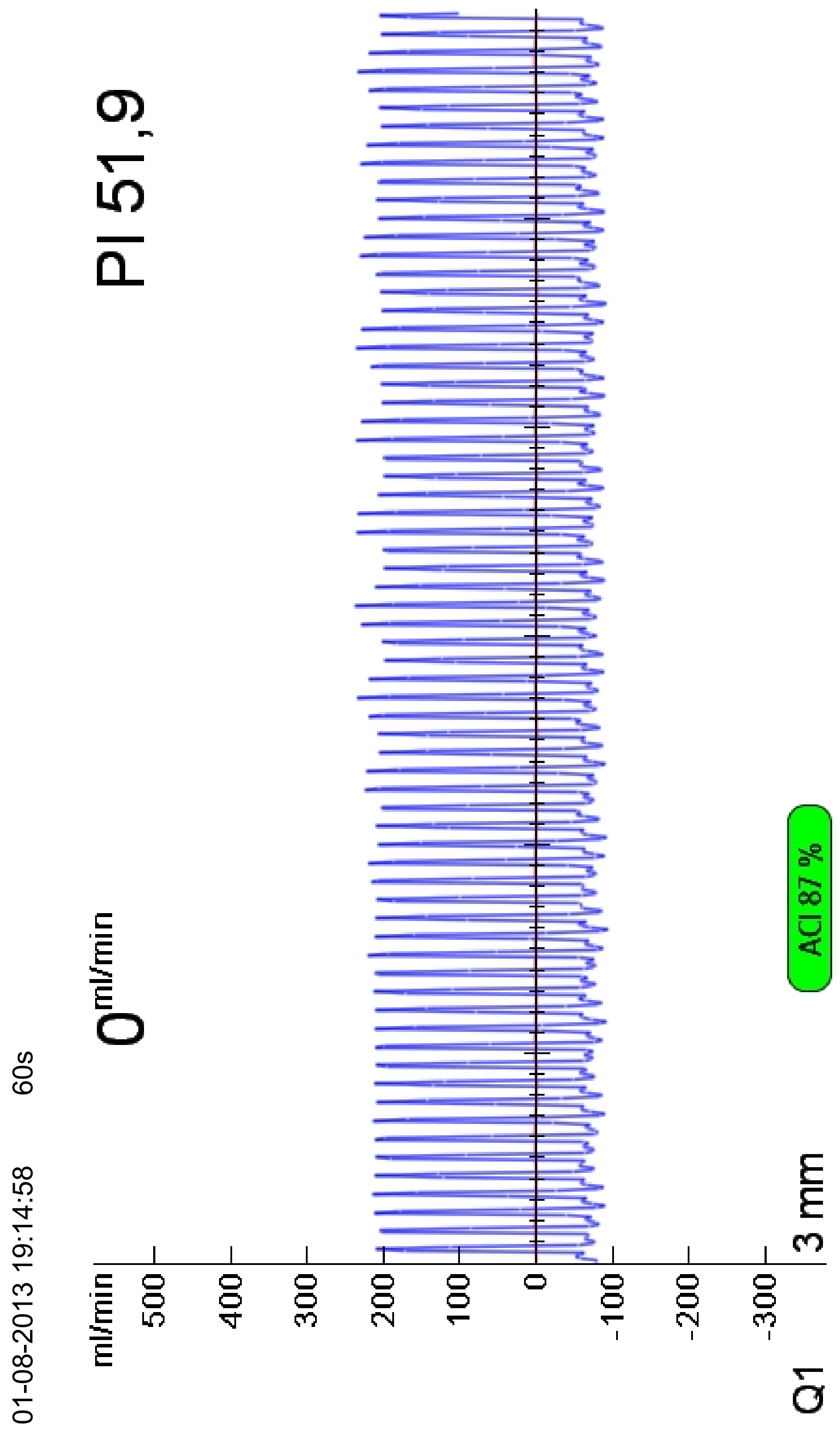

Skejby Sygehus

Urinvejskirurgisk afdeling K

Surgeon:

Operation Date: 31-07-2013 13:44:55

Patient Name: chris gris 10

Comments:

Patient ID:

Birthdate: Gender:

Height: Weight:

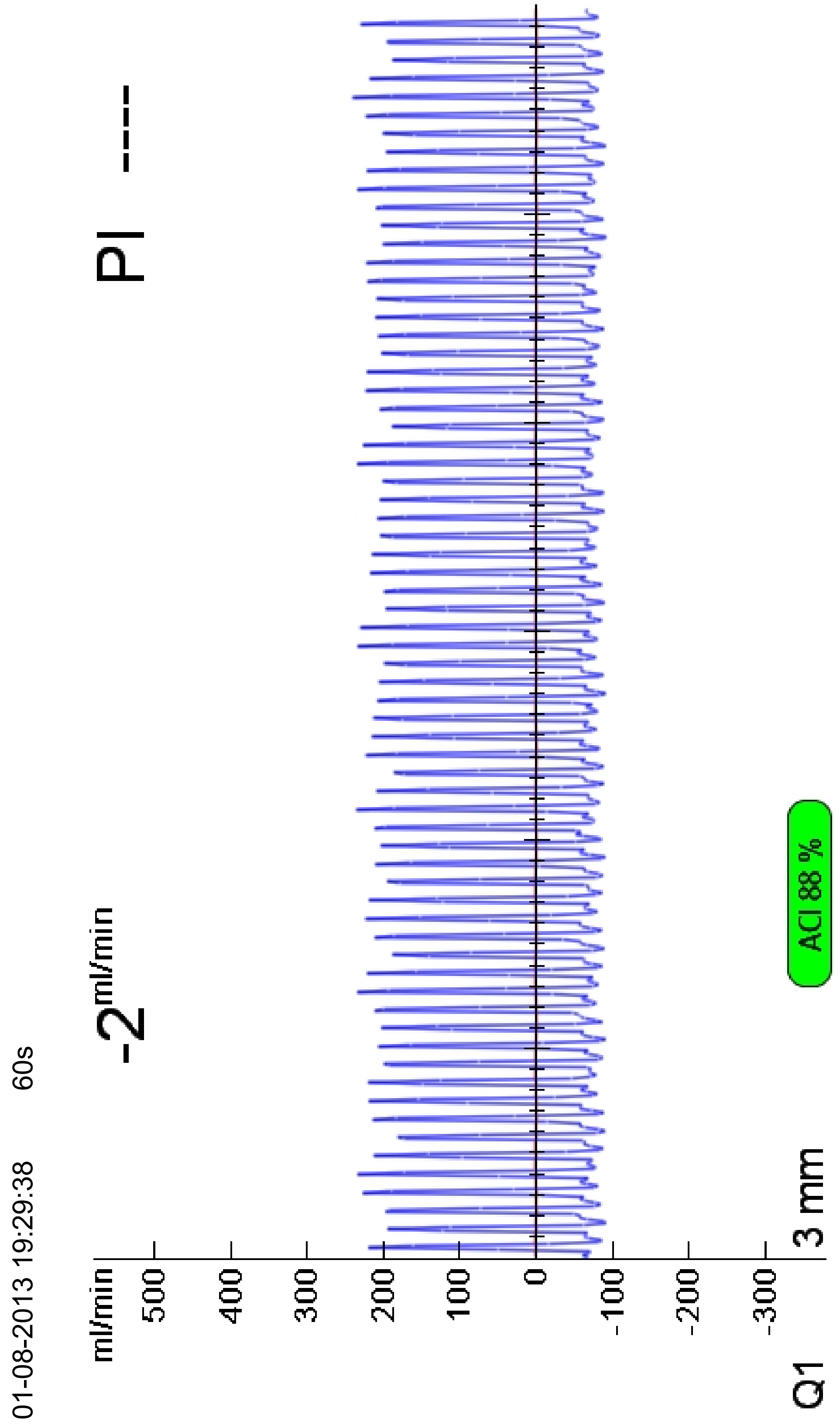

Skejby Sygehus

Urinvejskirurgisk afdeling K

MEDISTIM

Surgeon:

Operation Date: 31-07-2013 13:44:55

Patient Name: chris gris 10

Comments:

Patient ID:

Birthdate: Gender:

Height: Weight:

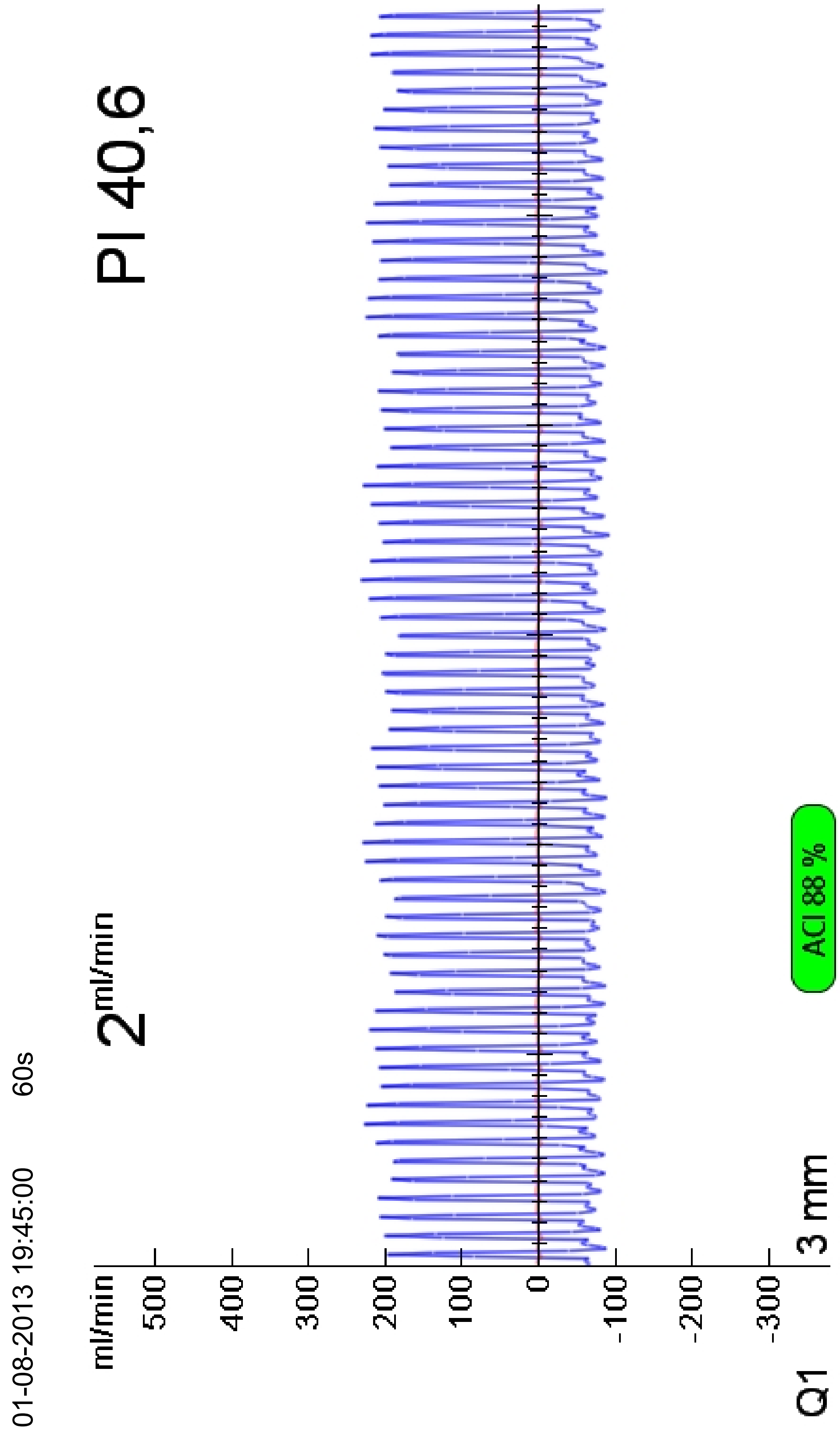

Supplement: S1 Data — (ZIP) [file pone.0178301.s001.zip › Supporting Information/Lumbal 3 d. 01.08.13/chris gris 10.pdf]
