# Supplementary material for: The unsuitability of implantable Doppler probes for the early detection of renal vascular complications – a porcine model for prevention of renal transplant loss
Source: PLoS One. 2017 May 25;12(5):e0178301. doi: 10.1371/journal.pone.0178301 (PMC5444816; doi:10.1371/journal.pone.0178301)

Skejby Sygehus

Urinvejskirurgisk afdeling K

Surgeon: Operation Date: 09-09-2013 10:38:11

Patient Name: gris 16, vk 4 Patient 09-09-2013

Comments:

Patient ID:

Birthdate: Gender:

Height: Weight:

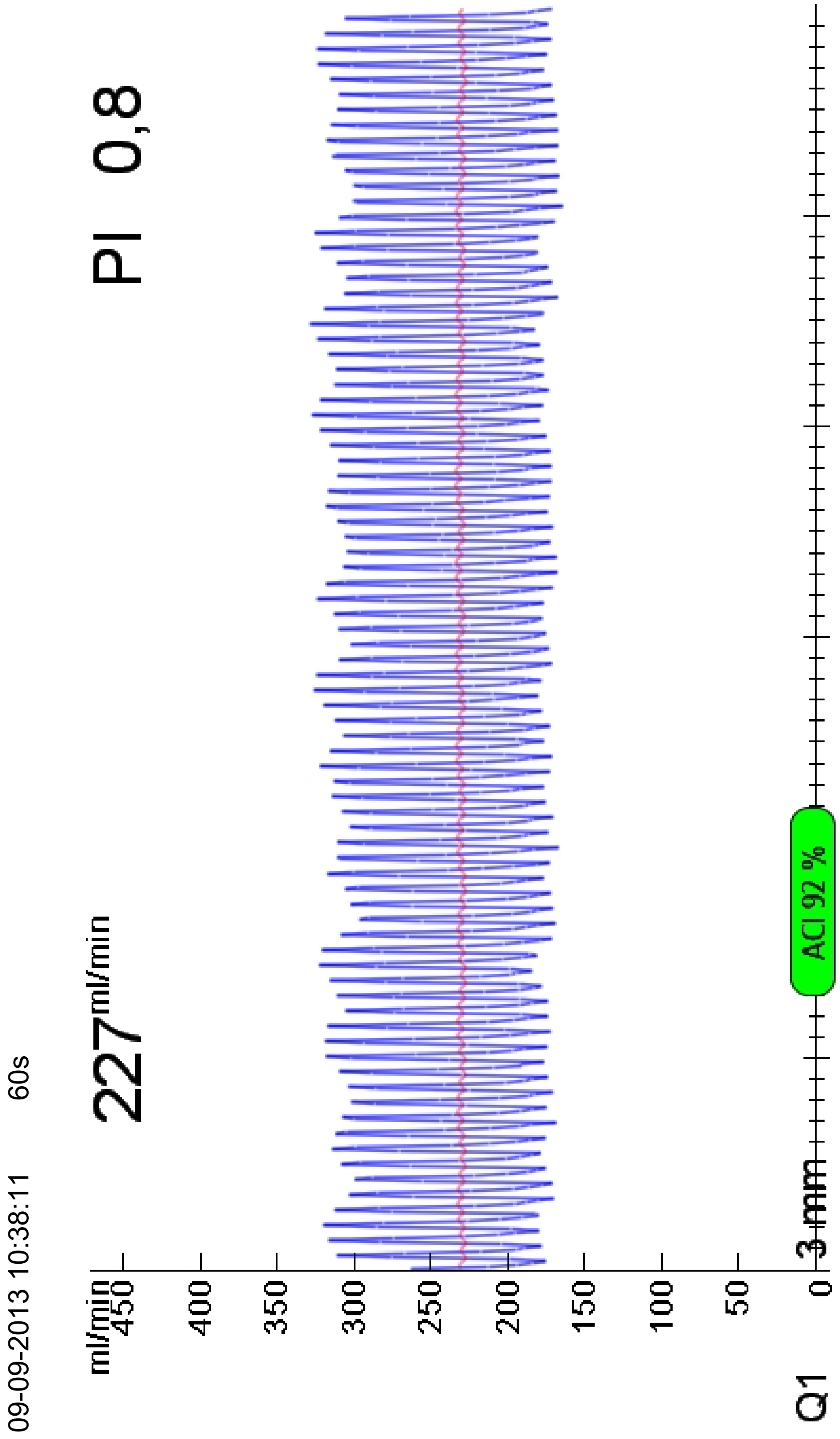

Skejby Sygehus

Urinvejskirurgisk afdeling K

Surgeon:

Operation Date: 09-09-2013 10:38:11

Patient Name: gris 16, vk 4 Patient 09-09-2013

Comments:

Patient ID:

Birthdate: Gender:

Height: Weight:

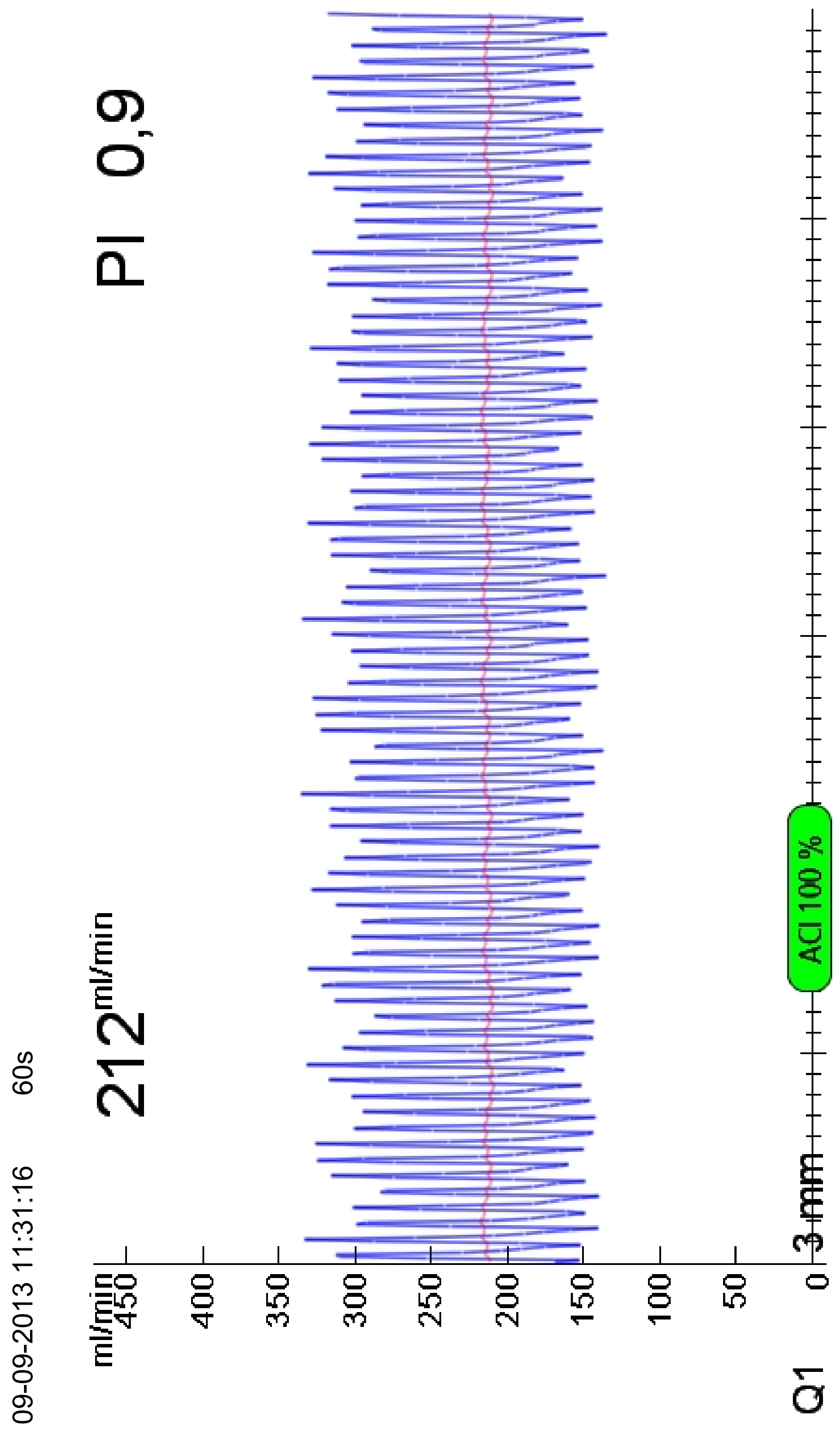

Skejby Sygehus

MEDISTIM

Urinvejskirurgisk afdeling K

Surgeon:

Operation Date: 09-09-2013 10:38:11

Patient Name: gris 16, vk 4 Patient 09-09-2013

Comments:

Patient ID:

Birthdate:

Gender:

Height:

Weight:

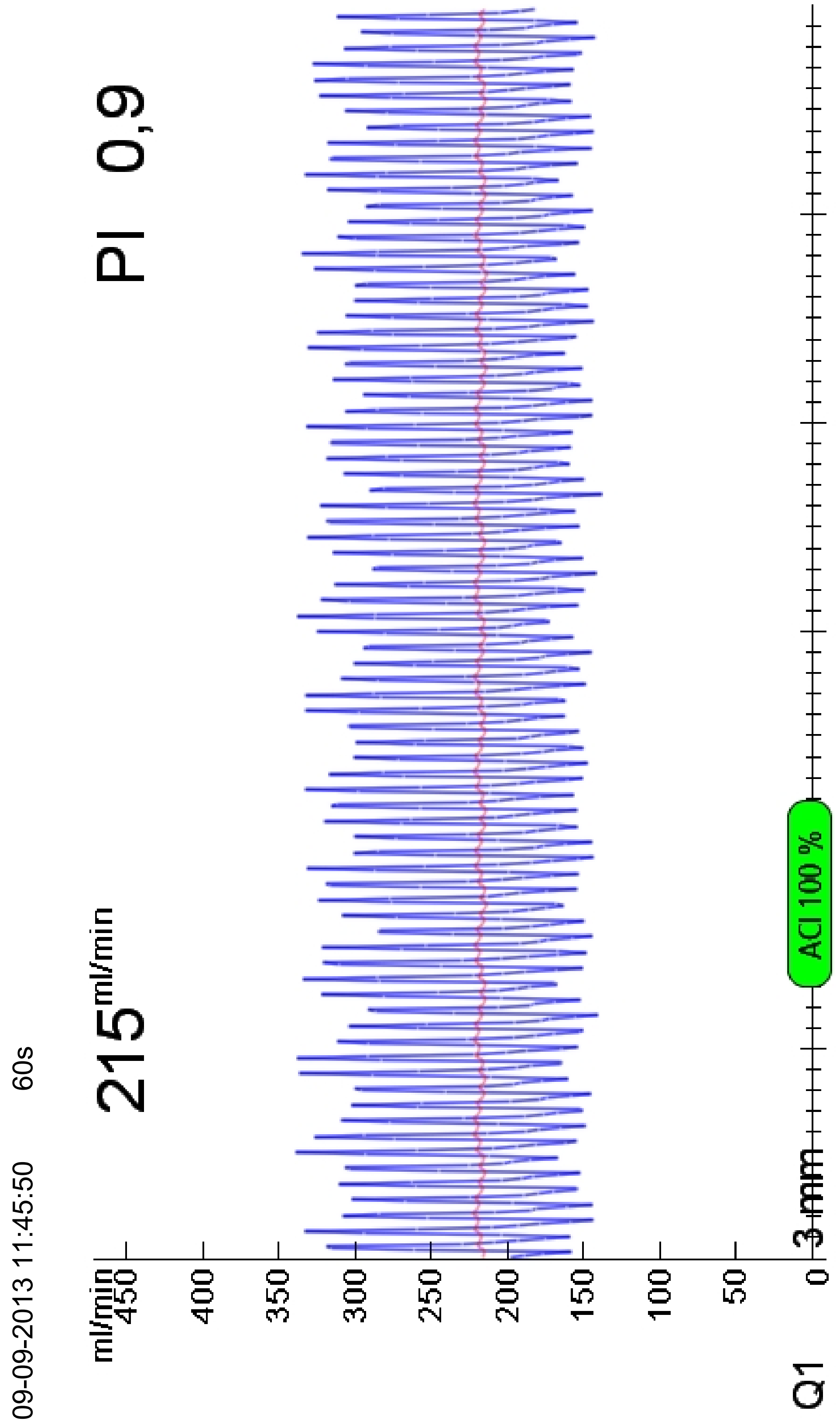

Skejby Sygehus

MEDISTIM

Urinvejskirurgisk afdeling K

Surgeon:

Operation Date: 09-09-2013 10:38:11

Patient Name: gris 16, vk 4 Patient 09-09-2013

Comments:

Patient ID:

Birthdate: Gender:

Height: Weight:

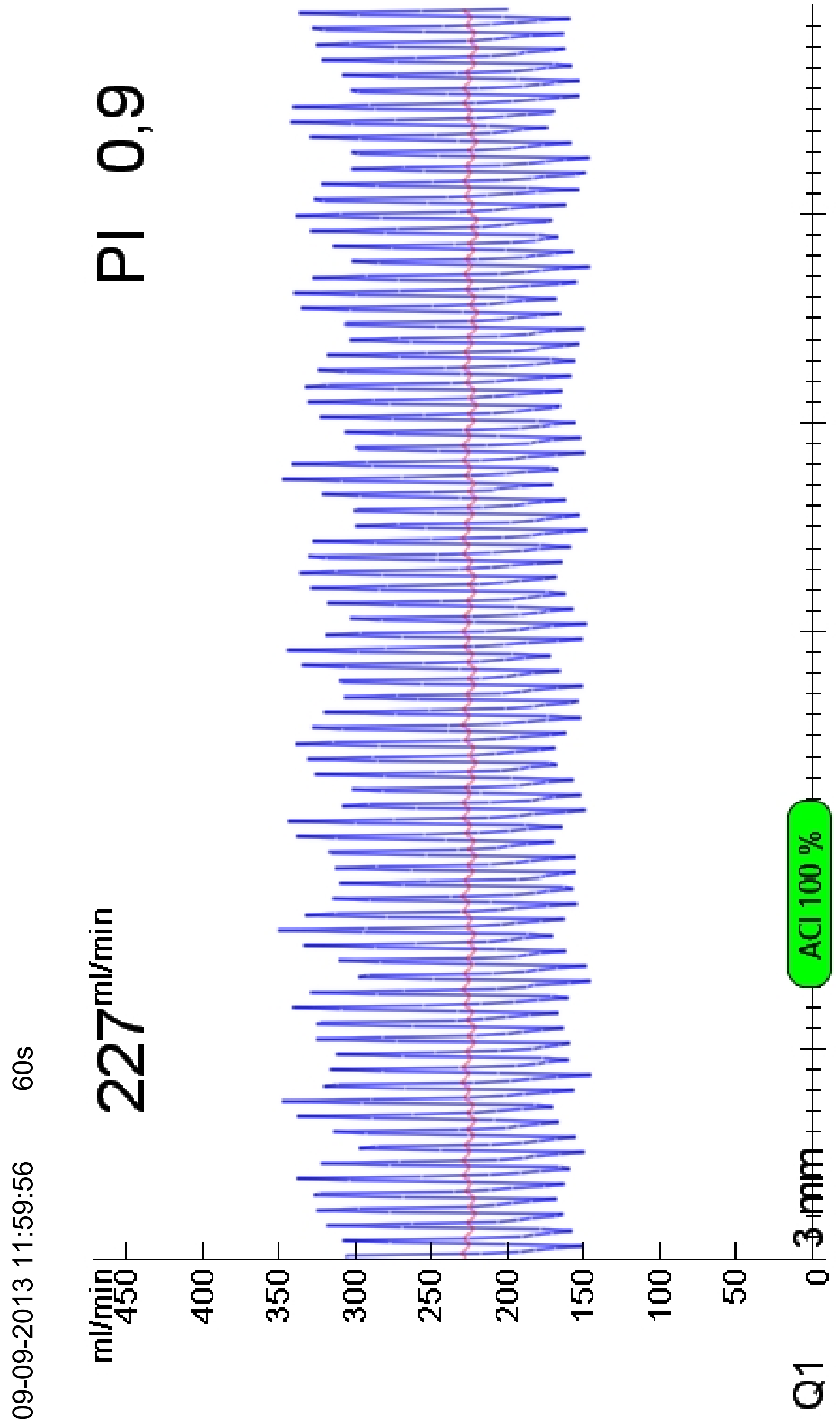

Skejby Sygehus

MEDISTIM

Urinvejskirurgisk afdeling K

Surgeon:

Operation Date: 09-09-2013 10:38:11

Patient Name: gris 16, vk 4 Patient 09-09-2013

Comments:

Patient ID:

Birthdate: Gender:

Height: Weight:

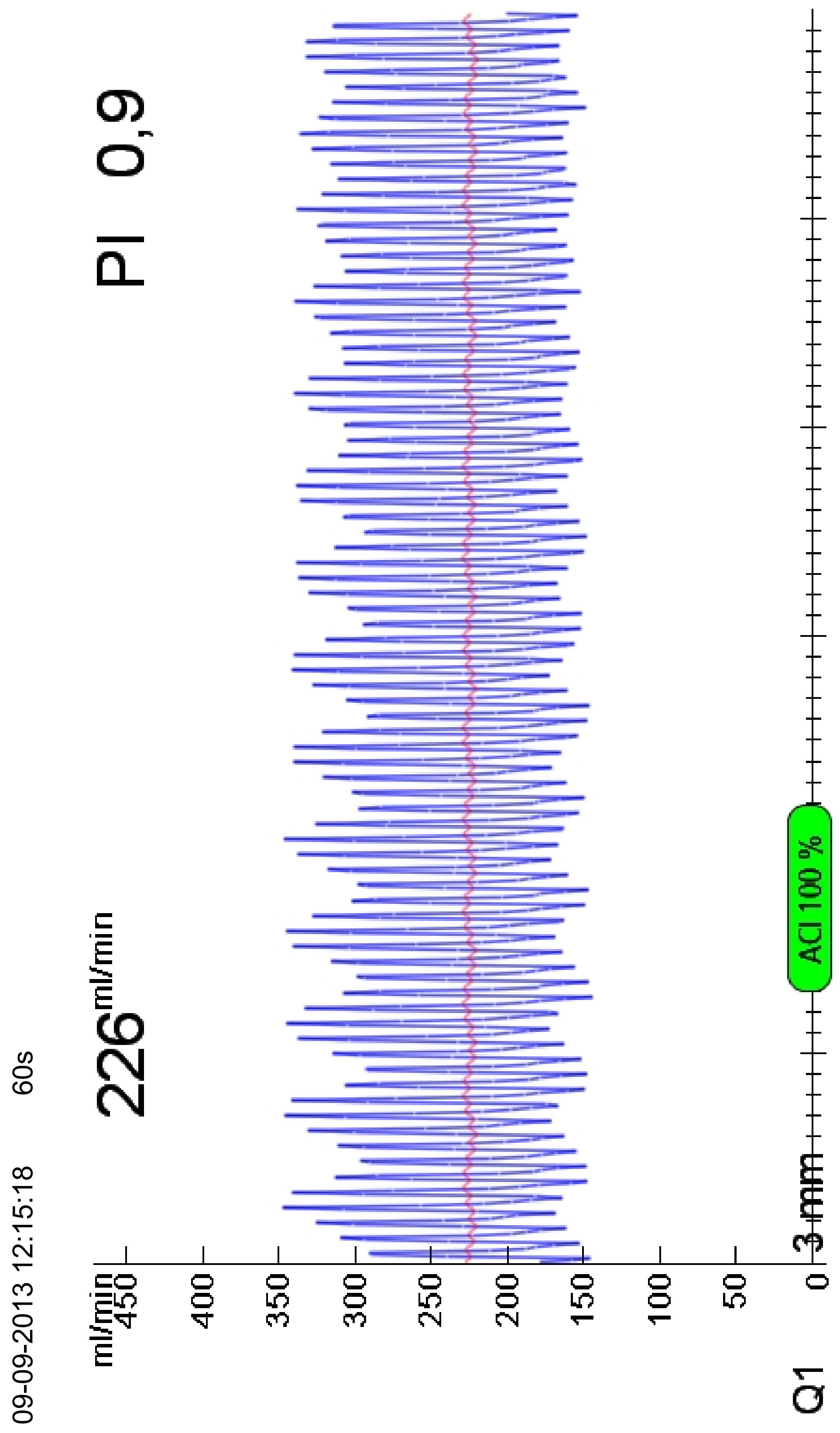

Skejby Sygehus

Urinvejskirurgisk afdeling K

Surgeon:

Operation Date: 09-09-2013 10:38:11

Patient Name: gris 16, vk 4 Patient 09-09-2013

Comments:

Patient ID:

Birthdate: Gender:

Height: Weight:

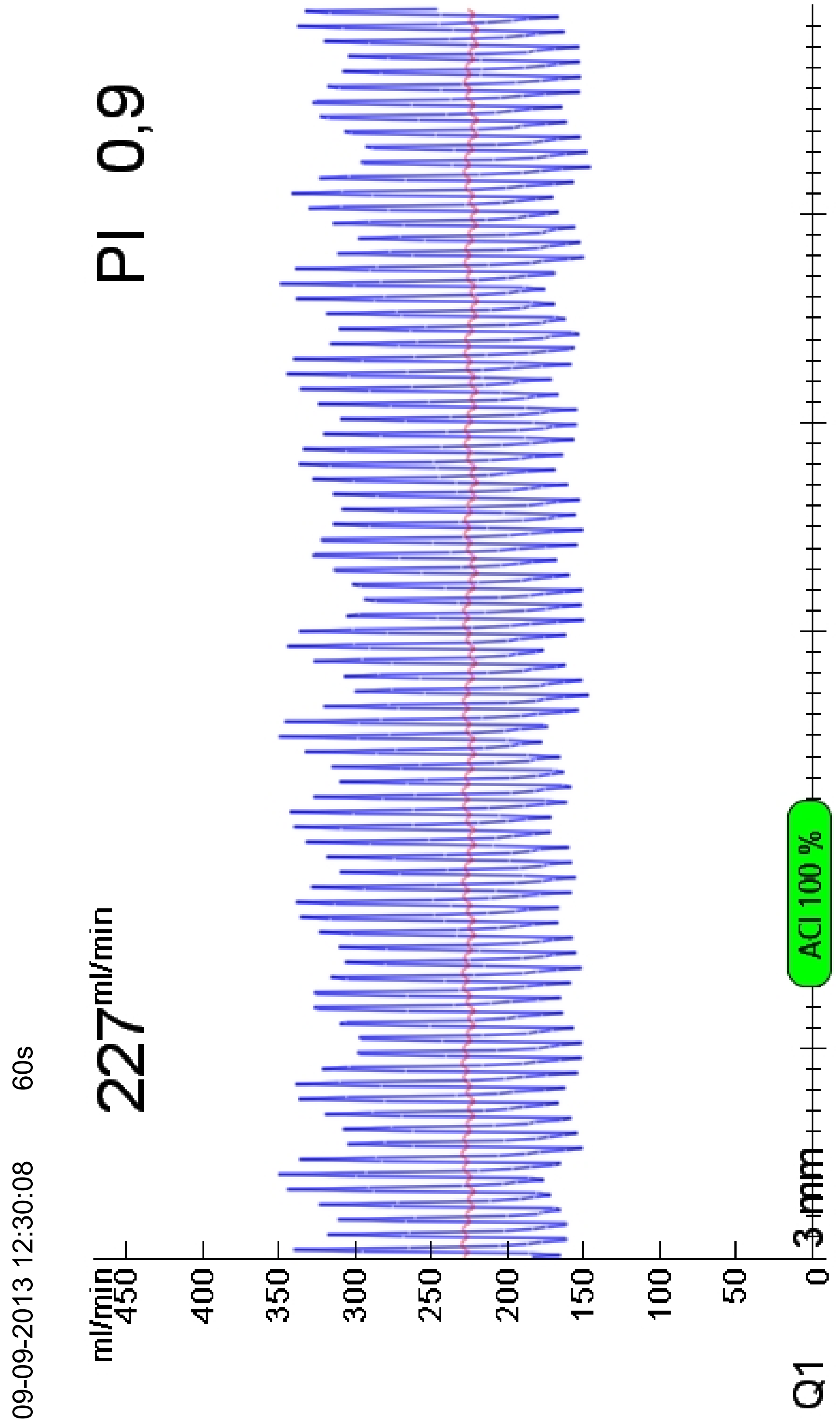

Skejby Sygehus

Urinvejskirurgisk afdeling K

Surgeon:

Operation Date: 09-09-2013 10:38:11

Patient Name: gris 16, vk 4 Patient 09-09-2013

Comments:

Patient ID:

Birthdate: Gender:

Height: Weight:

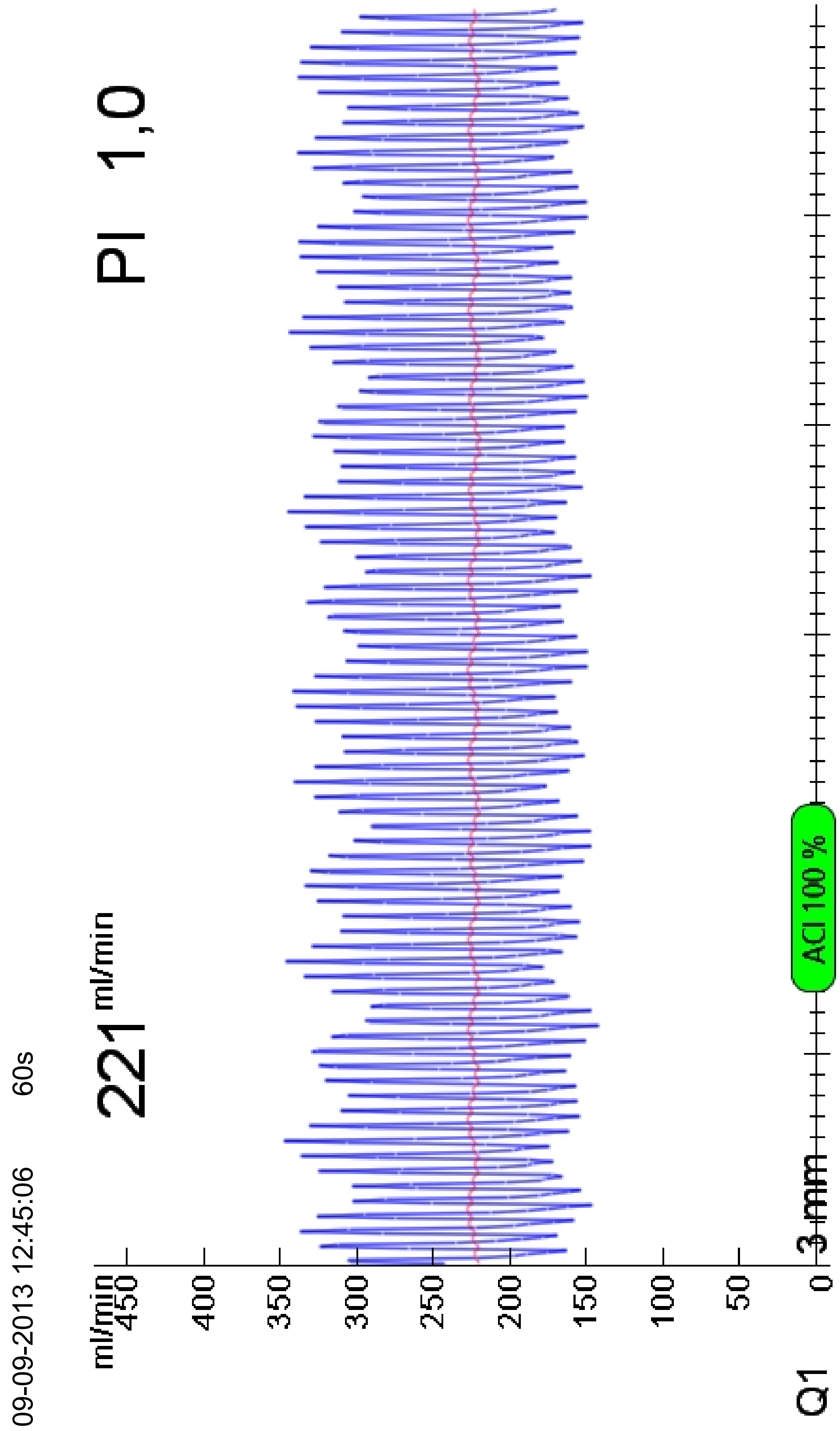

Skejby Sygehus

MEDISTIM

Urinvejskirurgisk afdeling K

Surgeon: Operation Date: 09-09-2013 10:38:11

Patient Name: gris 16, vk 4 Patient 09-09-2013

Comments:

Patient ID:

Birthdate: Gender:

Height: Weight:

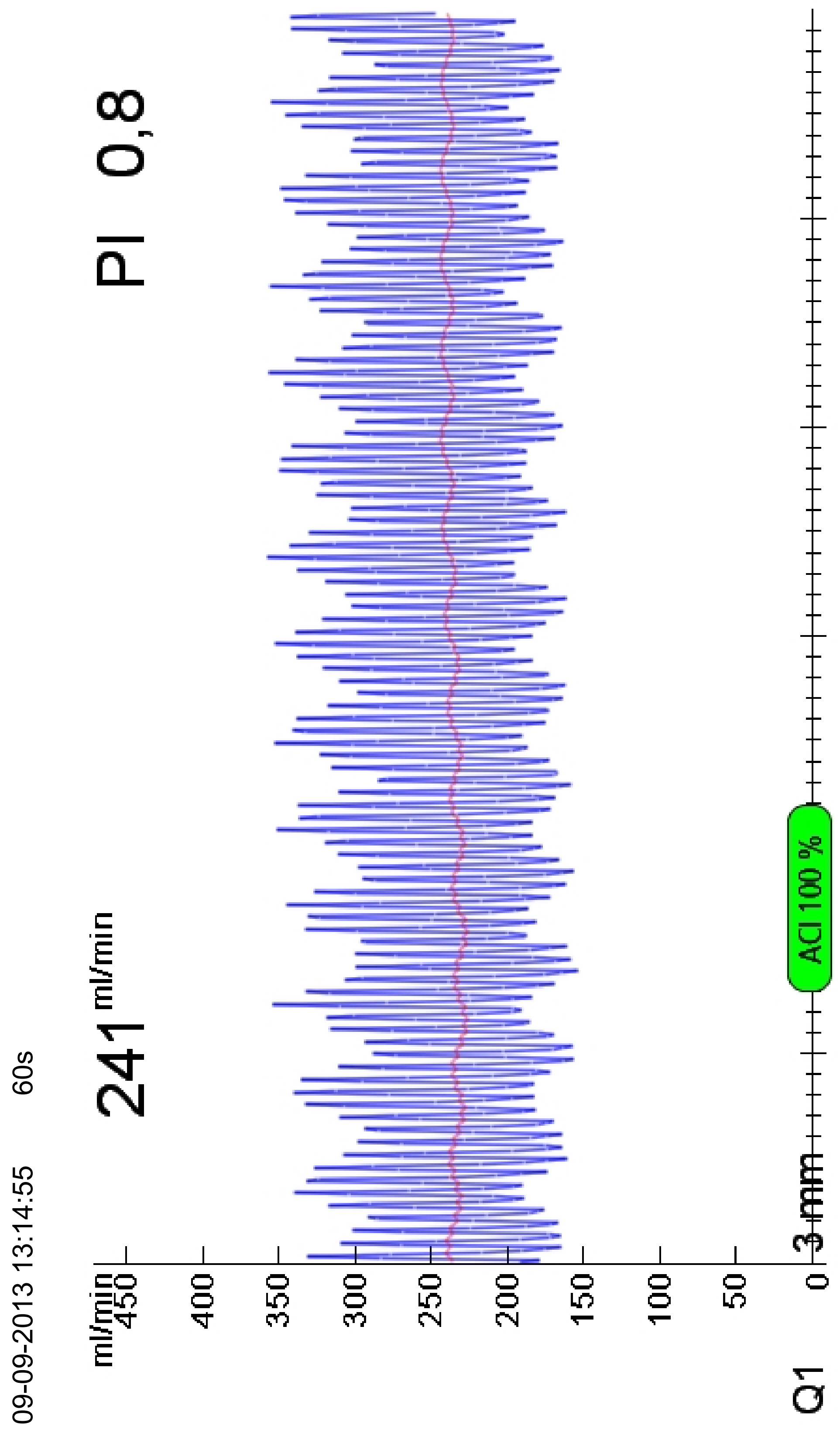

Skejby Sygehus

MEDISTIM

Urinvejskirurgisk afdeling K

Surgeon:

Operation Date: 09-09-2013 10:38:11

Patient Name: gris 16, vk 4 Patient 09-09-2013

Comments:

Patient ID:

Birthdate:

Gender:

Height:

Weight:

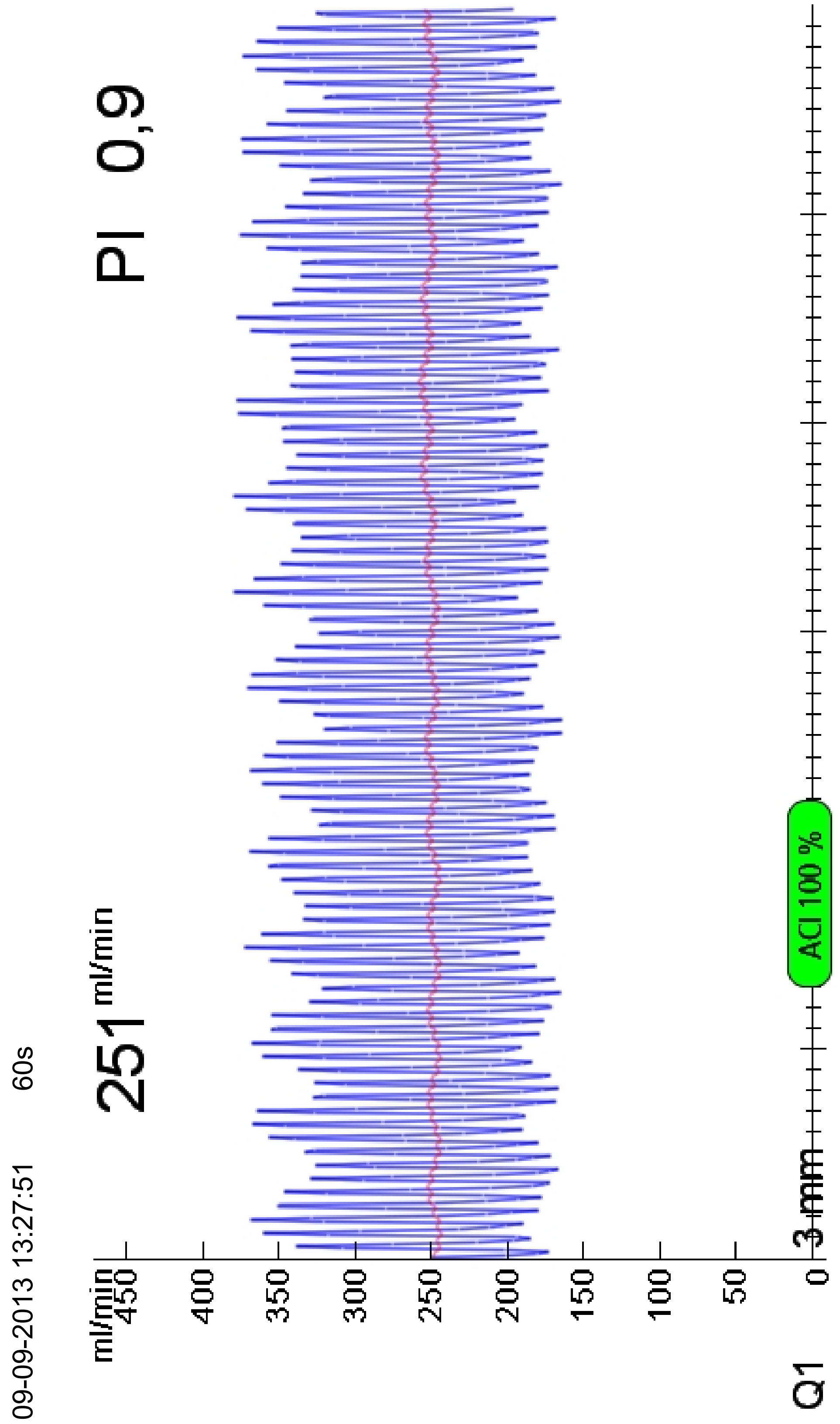

Skejby Sygehus

Urinvejskirurgisk afdeling K

Surgeon:

Operation Date: 09-09-2013 10:38:11

Patient Name: gris 16, vk 4 Patient 09-09-2013

Comments:

Patient ID:

Birthdate: Gender:

Height: Weight:

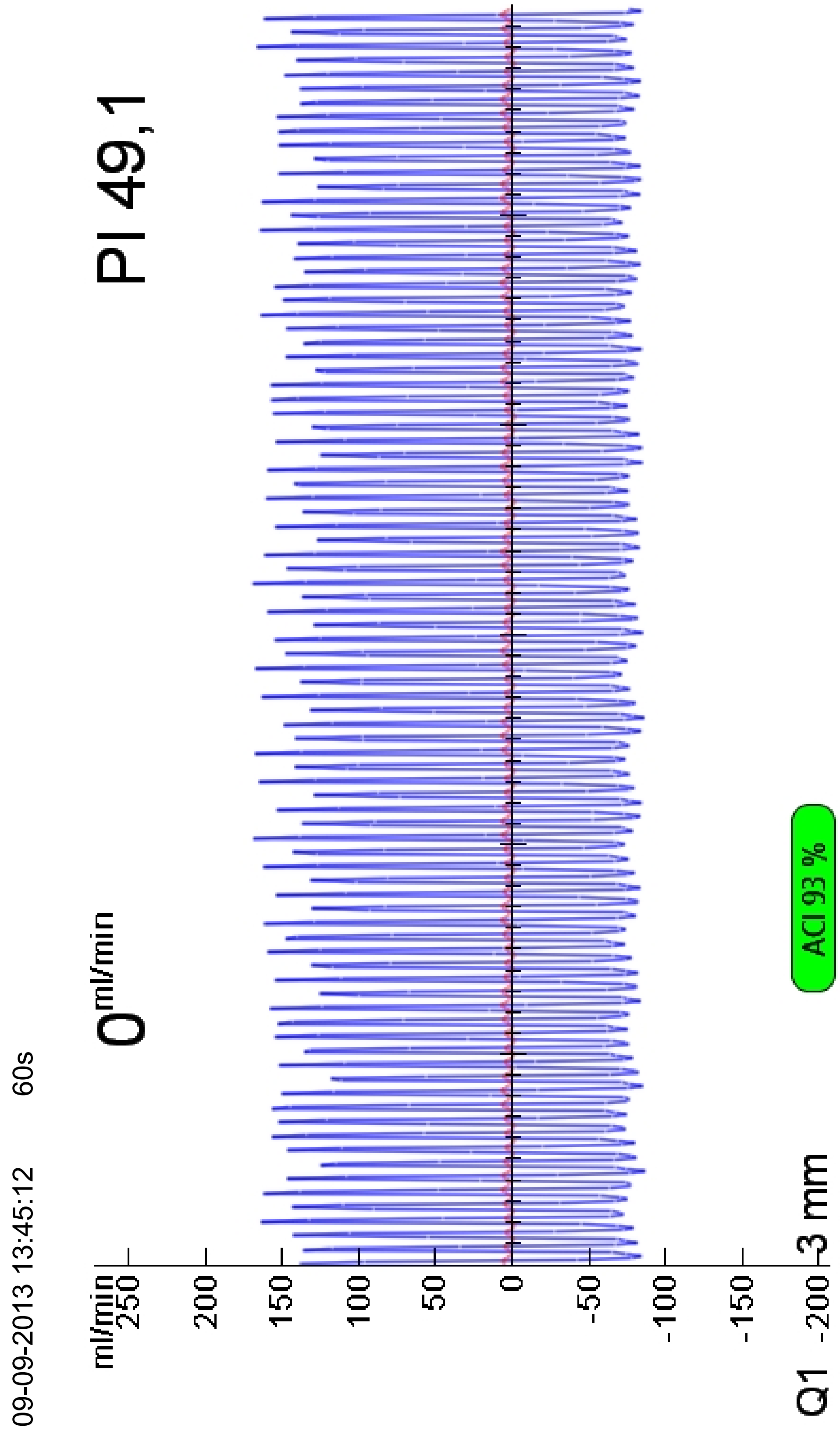

Skejby Sygehus

Urinvejskirurgisk afdeling K

MEDISTIM

Surgeon:

Operation Date: 09-09-2013 10:38:11

Patient Name: gris 16, vk 4 Patient 09-09-2013

Comments:

Patient ID:

Birthdate:

Gender:

Height:

Weight:

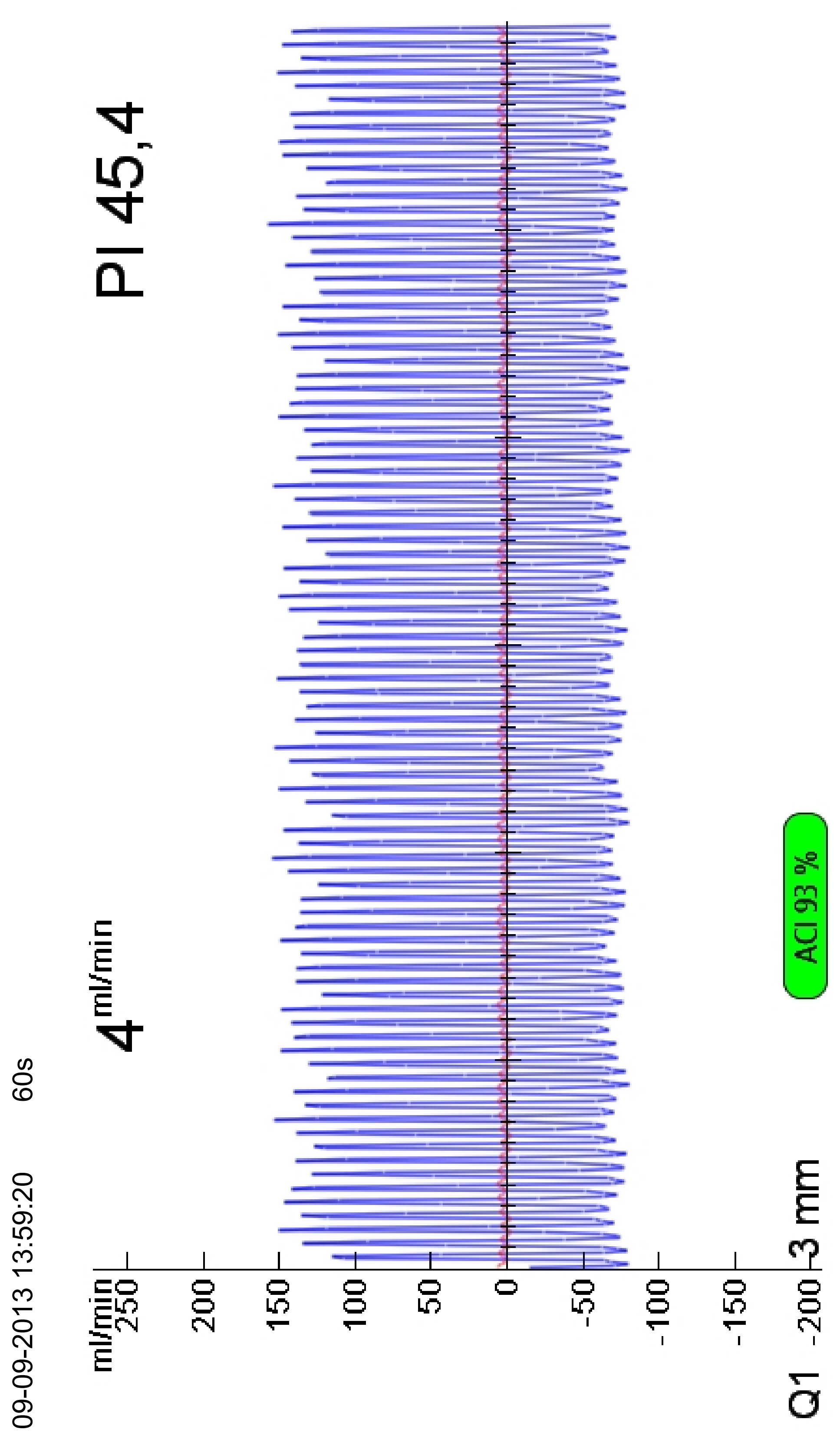

Skejby Sygehus

Urinvejskirurgisk afdeling K

Surgeon:

Operation Date: 09-09-2013 10:38:11

Patient Name: gris 16, vk 4 Patient 09-09-2013

Comments:

Patient ID:

Birthdate:

Gender:

Height:

Weight:

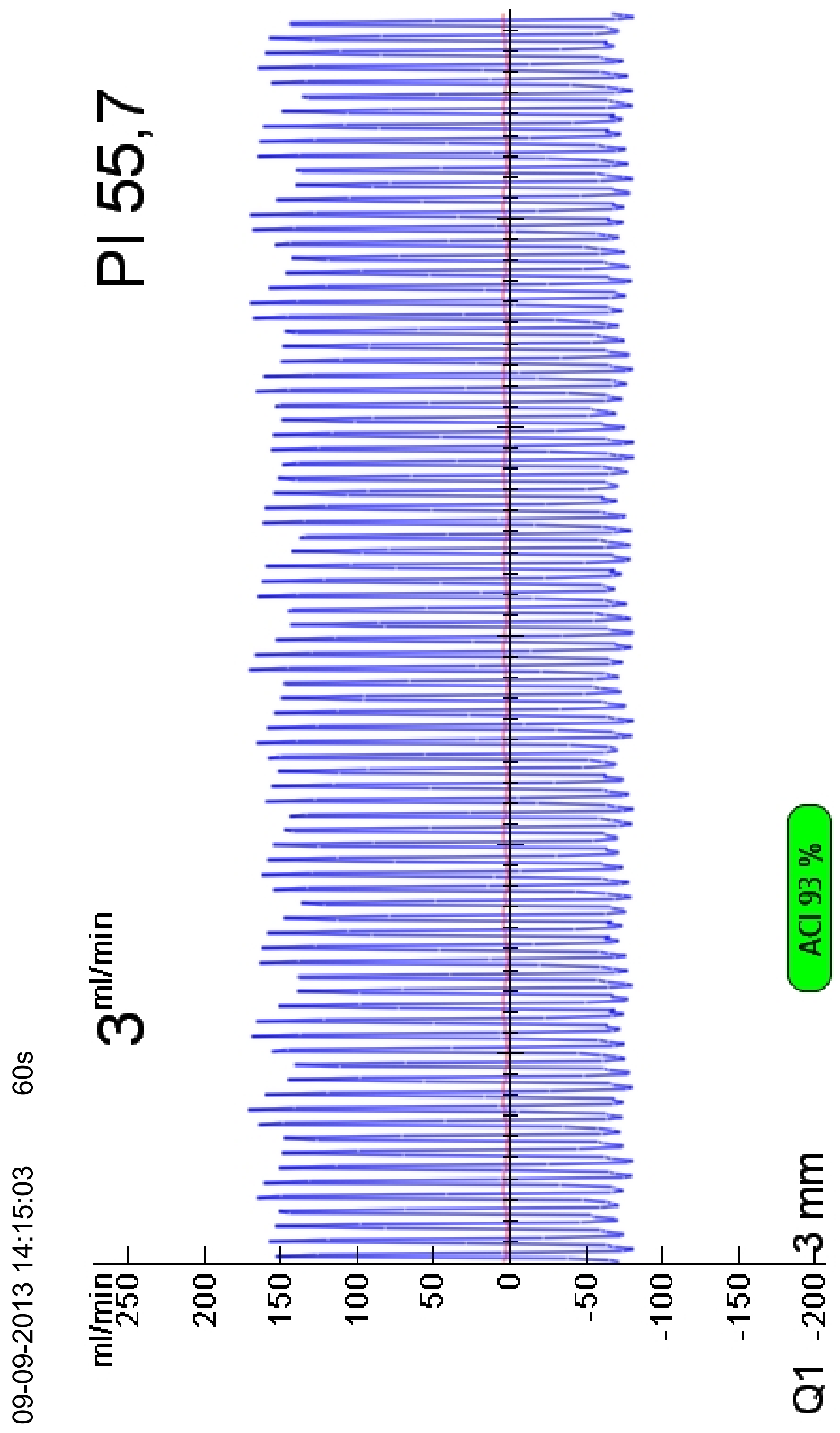

Skejby Sygehus

Urinvejskirurgisk afdeling K

MEDISTIM

Surgeon:

Operation Date: 09-09-2013 10:38:11

Patient Name: gris 16, vk 4 Patient 09-09-2013

Comments:

Patient ID:

Birthdate:

Gender:

Height:

Weight:

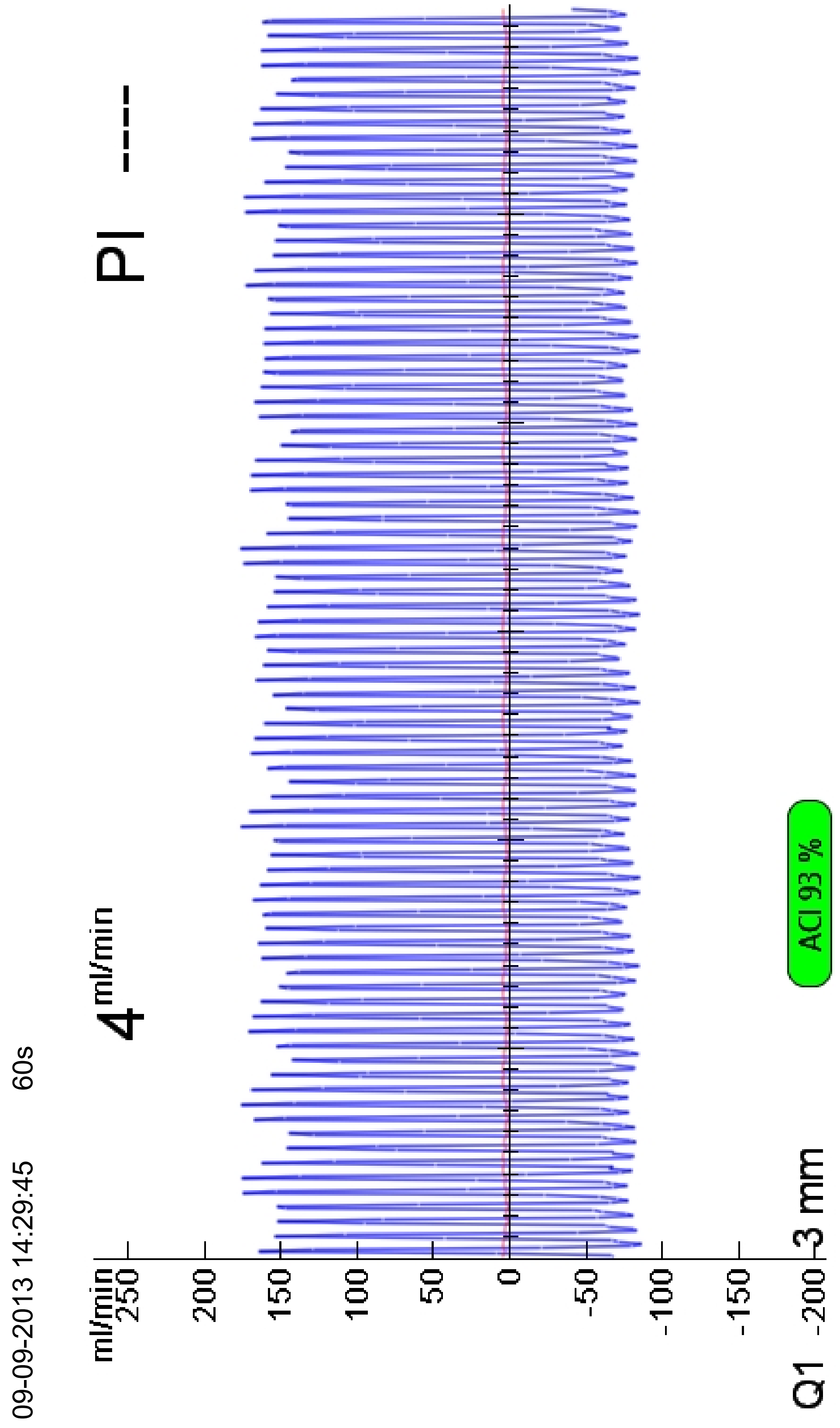

Skejby Sygehus

Urinvejskirurgisk afdeling K

Surgeon:

Operation Date: 09-09-2013 10:38:11

Patient Name: gris 16, vk 4 Patient 09-09-2013

Comments:

Patient ID:

Birthdate:

Gender:

Height:

Weight:

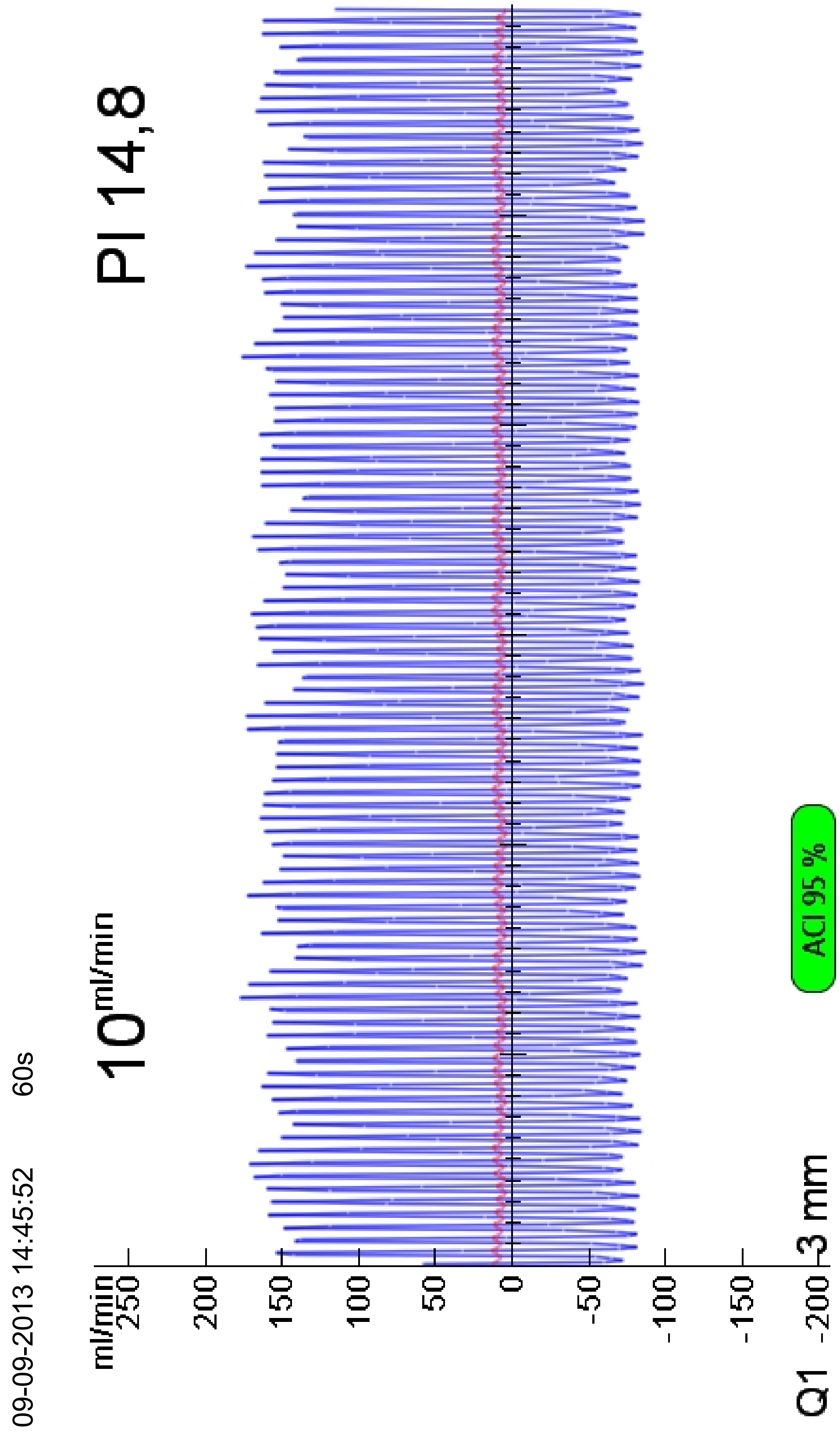

Skejby Sygehus

MEDISTIM

Urinvejskirurgisk afdeling K

Surgeon:

Operation Date: 09-09-2013 10:38:11

Patient Name: gris 16, vk 4 Patient 09-09-2013

Comments:

Patient ID:

Birthdate: Gender:

Height: Weight:

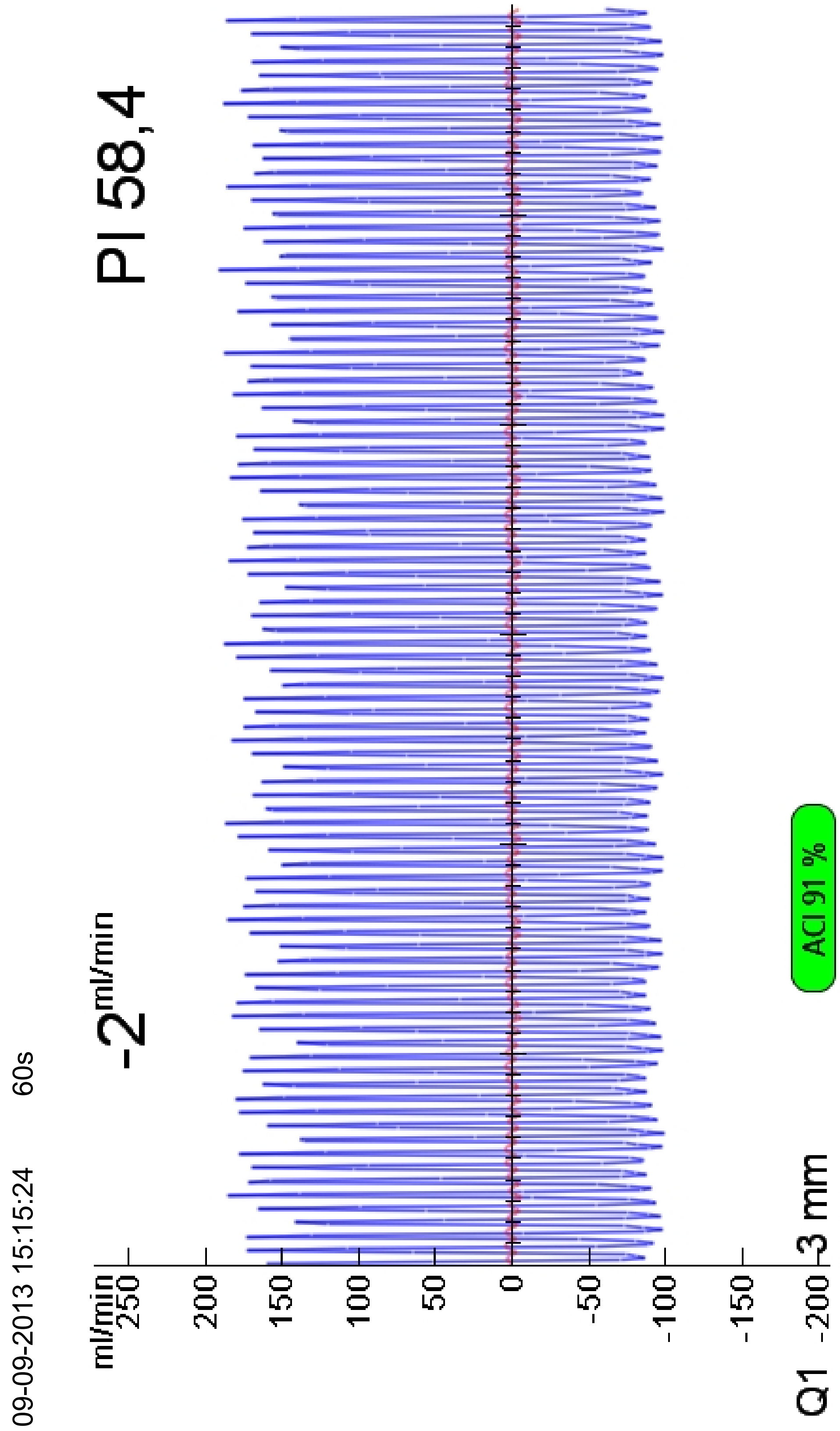

Skejby Sygehus

Urinvejskirurgisk afdeling K

Surgeon:

Operation Date: 09-09-2013 10:38:11

Patient Name: gris 16, vk 4 Patient 09-09-2013

Comments:

Patient ID:

Birthdate:

Gender:

Height:

Weight:

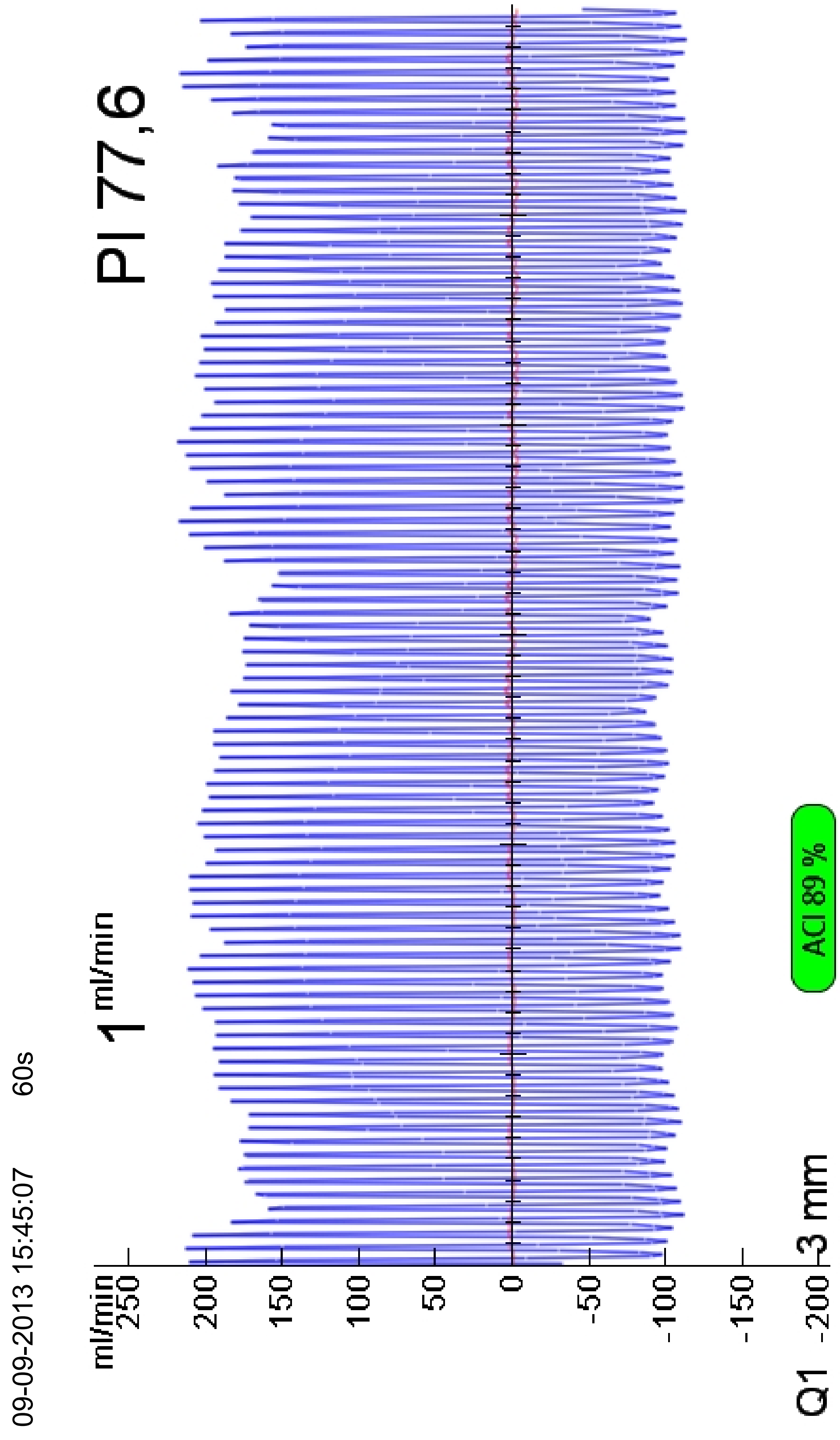

Skejby Sygehus

Urinvejskirurgisk afdeling K

MEDISTIM

Surgeon:

Operation Date: 09-09-2013 10:38:11

Patient Name: gris 16, vk 4 Patient 09-09-2013

Comments:

Patient ID:

Birthdate:

Gender:

Height:

Weight:

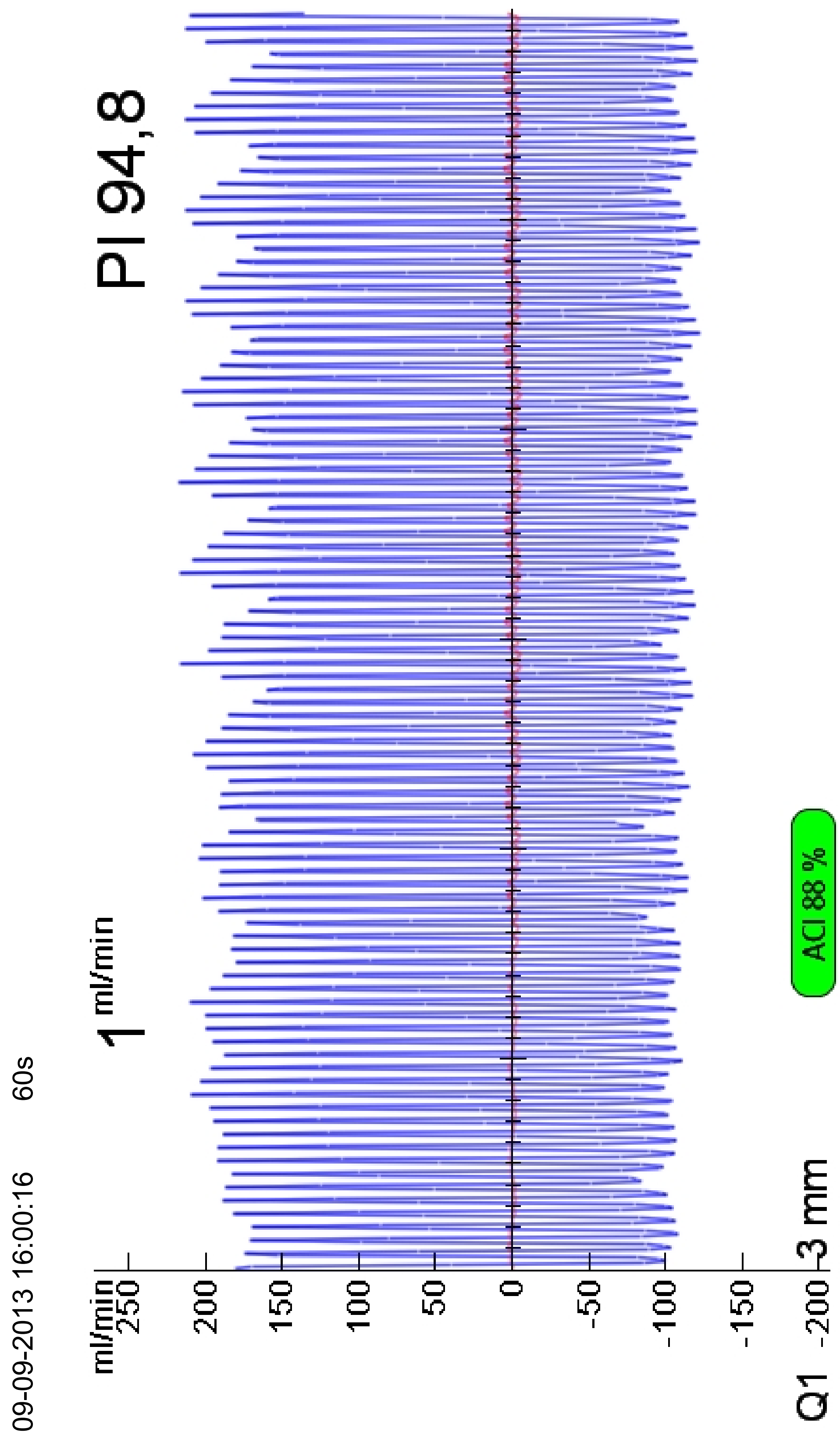

Skejby Sygehus

MEDISTIM

Urinvejskirurgisk afdeling K

Surgeon:

Operation Date: 09-09-2013 10:38:11

Patient Name: gris 16, vk 4 Patient 09-09-2013

Comments:

Patient ID:

Birthdate:

Gender:

Height:

Weight:

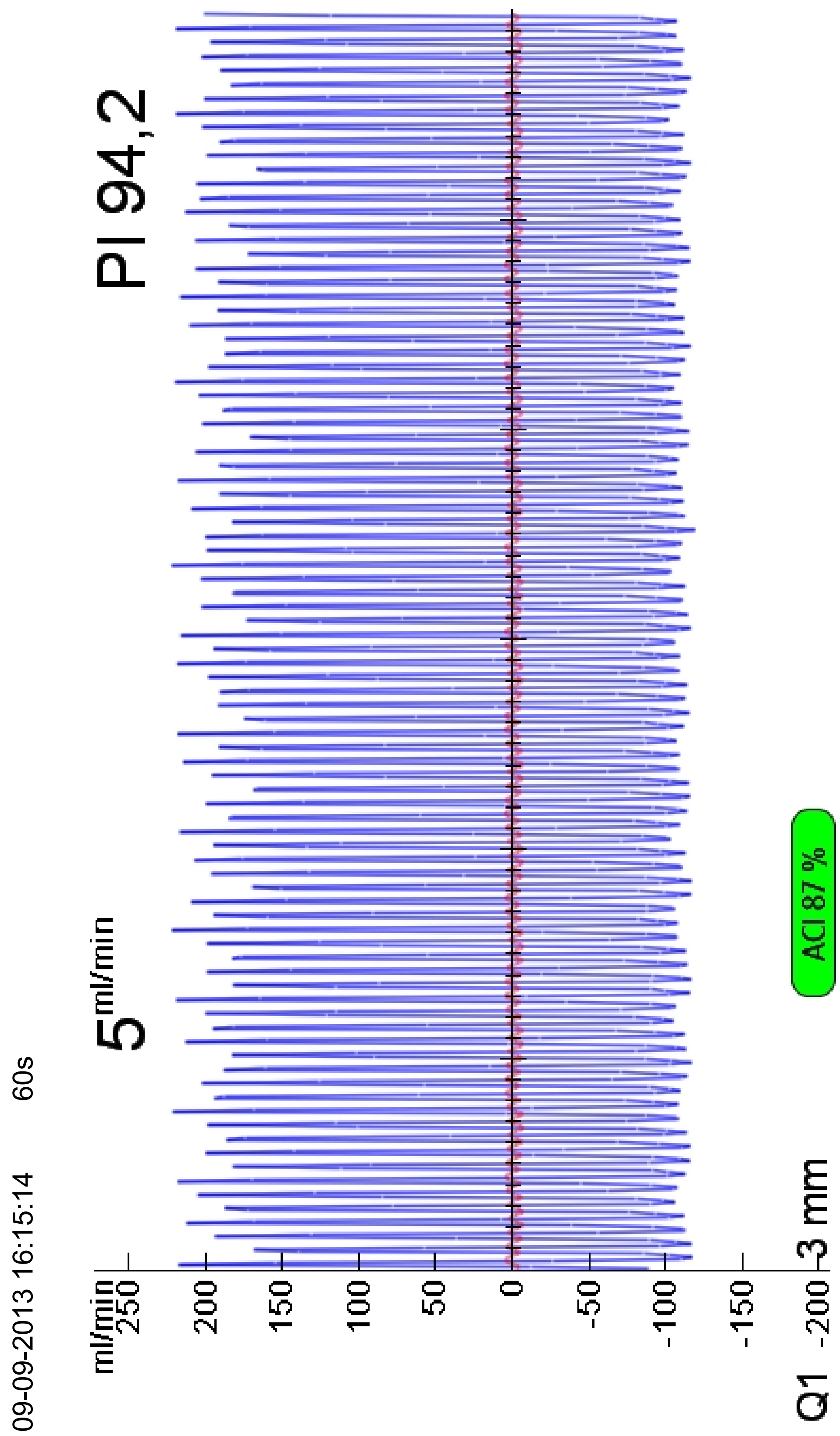

Skejby Sygehus

Urinvejskirurgisk afdeling K

MEDISTIM

Surgeon:

Operation Date: 09-09-2013 10:38:11

Patient Name: gris 16, vk 4 Patient 09-09-2013

Comments:

Patient ID:

Birthdate: Gender:

Height: Weight:

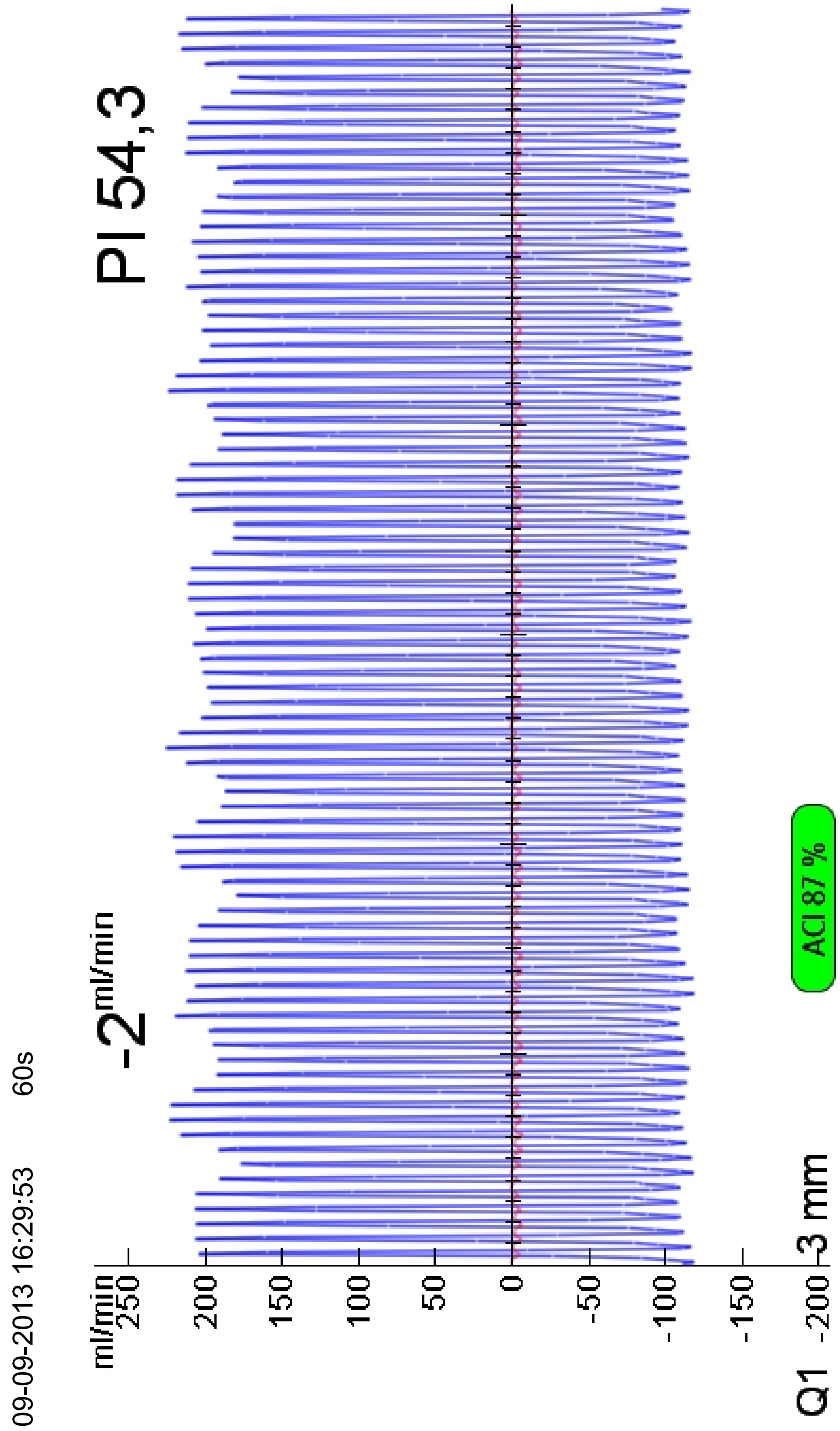

Supplement: S1 Data — (ZIP) [file pone.0178301.s001.zip › Supporting Information/Ven├╕s 4 d. 09.09.13/gris 16, vk 4 Patient 09-09-2013.pdf]
